# BUD-MI: a template that optimizes material intensity data collection and utilization

**DOI:** 10.1007/s44498-026-00044-w

**Published:** 2026-03-13

**Authors:** Maud Lanau, Charles Gillott, Will Mihkelson, Kimberlee Zamora, Peter Berrill, Niko Heeren, Georg Schiller, Ruichang Mao, Mohit Arora, Karin Gruhler, Gang Liu, Hiroki Tanikawa, Danielle Densley Tingley

**Affiliations:** 1 Division of Sustainable Built Environments, Department of Architecture and Civil Engineering, Chalmers University of Technology, Gothenburg, Sweden; 2 School of Mechanical, Aerospace and Civil Engineering, The University of Sheffield, Sheffield, SE-41296 UK; 3 WSP UK Limited, London, UK; 4 Construction Management Department, School of Engineering, Widener University, Chester, PA US; 5 Institute of Environmental Sciences (CML), Leiden University, Leiden, The Netherlands; 6 Construction Office, City of Zurich, Zurich, Switzerland; 7 Leibniz Institute of Ecological Urban and Regional Development, Dresden, Germany; 8 Faculty of Geographical Science and Engineering, Henan University, 450046 Zhengzhou, China; 9 Net Zero Centre, Department of Engineering, King’s College London, London, UK; 10 College of Urban and Environmental Sciences, Peking University, Beijing, 100871 China; 11 Graduate School of Environmental Studies, Nagoya University, Nagoya, Japan

**Keywords:** Industrial ecology, Material intensity, Data collection template, Building material stock, Cumulative research, Circular economy

## Abstract

Mapping construction material stocks is essential for understanding socioeconomic metabolism and informing the circular economy. However, spatial material stock studies often produce coarse results owing to challenges in material intensity development. This paper introduces BUD-MI (Bottom-Up Data: Material Intensity), a material intensity data collection template designed with three core objectives: streamlining the data collection process during building sampling, supporting cumulative research while ensuring project-specific relevance, and enhancing the utility of results for the construction industry. BUD-MI aims to assist researchers, students, and construction practitioners in developing material intensity data in line with these three objectives. The development process required identifying material intensity challenges, eliciting requirements, mapping relevant domain work and creating missing ones, and assembling them all into BUD-MI. The template consists of three data input tabs, two mini-tools for data input assistance, four result generation tabs, and additional tabs for background data and ancillary information. A case study in Sheffield, UK, illustrates BUD-MI’s functionalities, enabling bespoke material intensity results to be disaggregated across building elements and components. BUD-MI supports future material intensity data collection efforts, ensuring data quality, granularity, comparability, transferability, and availability, thereby advancing socioeconomic metabolism and circular economy research and practice.

**Supplementary Information:**

The online version contains supplementary material available at 10.1007/s44498-026-00044-w.

## Introduction

Building stocks account for extensive material demand, waste generation, and embodied carbon emissions throughout their life cycle (Cao et al., 2020; Pauliuk et al., 2021). The construction sector is thus a critical area for decarbonization, playing a key role in meeting several UN Sustainable Development Goals (especially SDGs 9, 11, 12, and 13) and the Paris Agreement’s 1.5 °C target. Because the composition and size of building material stocks (MS) are fundamental for analyzing interactions between built environment development and resource-related environmental impacts, it is essential to thoroughly characterize MS.

Two interconnected research fields, socioeconomic metabolism (SEM) and circular economy (CE), are concerned with achieving a granular understanding of the built environment’s MS. The goal of SEM research is to systematically survey societies’ use of resources to uncover patterns and analyze their underlying causes (Haberl et al., 2019). For example, SEM researchers successfully demonstrated interrelations between resource accumulation and socioeconomic indicators such as population, economic growth, and service levels (Fishman et al., 2015; Müller et al., 2011; Wiedenhofer et al., 2023). The goal of CE research is to explore how to transition to an economy with minimal resource use and waste (Geissdoerfer et al., 2017), which requires a granular and spatial understanding of MS to support CE strategizing (Schiller et al., 2025; Wuyts et al., 2022; Yang et al., 2023).

**Fig. 1:**
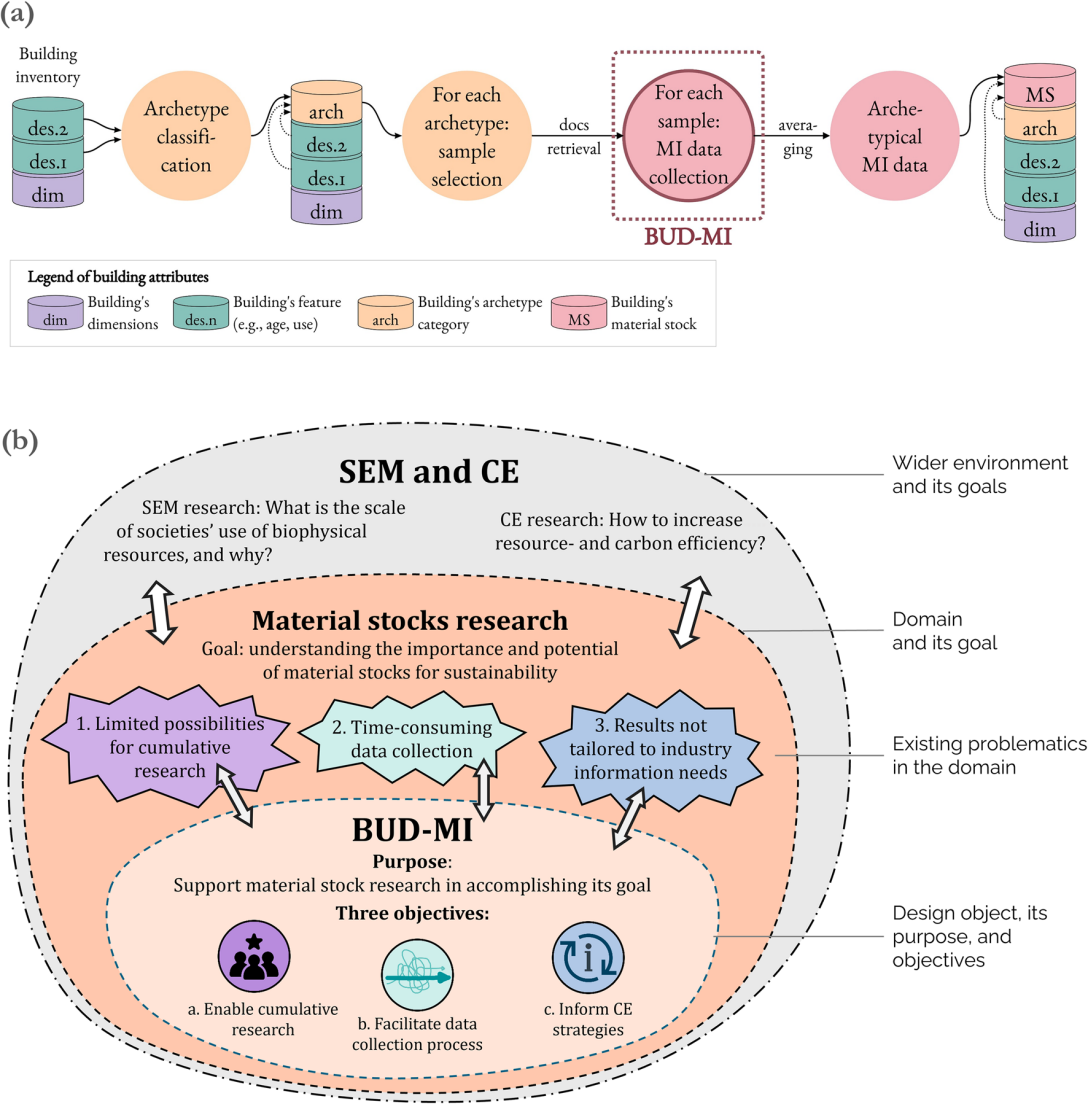
The scope of BUD-MI operation. **a** BUD-MI is designed for a specific stage of material stock modeling–the collection of material intensity (MI) data. **b** BUD-MI operates within the domain of material stock research, itself situated within the research environments of socioeconomic metabolism (SEM) and circular economy (CE). The identified problematics of MIs (1–3) are translated into the three objectives of BUD-MI (**a**–**c**). Based on the model of the design concept by Ralph & Wand (2009)

Most studies characterize MS using a bottom-up approach, multiplying building inventory data with material intensity (MI) data (Fig. 1a). A few years ago, the availability of both data was critically low (Lanau et al., 2019) and since then, many studies focused on addressing the building inventory data gap using various digital mapping techniques (see Sect. 4.2.3). Consequently, the number of spatial MS studies is rapidly growing (Aldebei & Dombi, 2021). But inventory data represents only half of the equation, and the other half, MI data, continues to face numerous challenges (see Sects. 1.1 and 3.2). MI has become a bottleneck for achieving highly detailed MS results, and enhancing its collection and usability would unlock analytical power for SEM and CE.

### Material intensities

Building MIs reflect the type and quantity of materials stocked per unit of measurement of a building; as such, they have various applications. Coupled with a building inventory, MIs enable piece-by-piece modeling of a building stock. If the inventory is spatialized, a so-called resource (or material) cadaster is generated, displaying the location, type, and quantity of materials within a building stock. In demand-driven prospective stock studies, MIs convert future demand for building floor area into material demand, and future demolition into waste generation (Heeren & Hellweg, 2016; Huang et al., 2018; Müller, 2006). MIs can also be combined with embodied carbon factors to calculate the carbon embodied in buildings (Schandl et al., 2020; Stephan & Athanassiadis, 2017). The various applications of MIs demonstrate their value for both environmental planning tasks (e.g., sustainable spatial planning, waste management planning, raw materials planning) and the development of new business ideas in circular construction (e.g., Madaster, 2024).

Given their importance, efforts to increase the availability of MIs have intensified in recent years. National datasets have been developed for e.g., Sweden (Gontia et al., 2018), China (Yang et al., 2020), Denmark (Lanau & Liu, 2020), Germany (Ortlepp et al., 2018), the United States (Berrill & Hertwich, 2021), the Netherlands (Sprecher et al., 2022), Canada (Guven et al., 2022), and Finland (Kaasalainen et al., 2023). Internationally, consolidation efforts include the open MI database of Heeren and Fishman (2019) (hereafter ‘open MI database’), the global residential MI database of Marinova et al. (2020), and the RASMI database combining existing and synthetic data (Fishman et al., 2024).

Still, MI data faces crucial challenges. Relative to the world’s one billion buildings (Microsoft, 2024), they remain scarce (Fishman et al., 2024), and residential buildings and developed countries are overrepresented (Marinova et al., 2020), calling for an *en masse* development of MI data (Augiseau and Kim, 2021; Arceo et al., 2023). Yet, their ad-hoc creation to fit project-specific requirement causes inconsistencies in terminology, metrology, and granularity (Schiller et al., 2019; Sprecher et al., 2022), constraining their use to the project only and limiting their comparability and transferability across diverse applications.

### Rationale, aim, and objectives

The rationale behind this paper is that the informational value of MIs can significantly increase if their development (i.e., creation) follows a structured, systematic, and informed process. In these regards, templates are particularly useful as well as facilitating knowledge transfer (Jensen & Szulanski, 2007). As such, this work aims to develop a template for MI data collection. This paper introduces BUD-MI (Bottom-Up Data: Material Intensity), a new template for MI data collection in buildings, with three core objectives: (1) to facilitate a structured and time-efficient data collection process during building sampling, (2) to support cumulative research while still ensuring project-specific results, and (3) to increase the usefulness of results for the construction industry with regard to circularity strategizing. Details on how these objectives were derived are provided in Sect. 3.2.

BUD-MI’s design was guided by the principle of leveraging existing MI-related knowledge and methodologies as extensively as possible. Its intended users are anyone collecting or refining MI data, primarily researchers and students in MS modeling and related fields (e.g., industrial ecology, building life cycle assessment, architecture, construction management). It may also assist construction practitioners seeking to understand their buildings’ material profile, although it is not intended for routine integration into professional workflows for which dedicated quantity take-offs tools already exist (e.g., in building information modeling (BIM) software). Rather, in line with Objective 3, BUD-MI intends to support CE strategizing by facilitating knowledge transfer from CE and MS research to practice, where a clear narrative and shared language are essential to motivate and shape innovation (Ninan et al., 2022).

### Structure of the paper

Section 2 introduces the four-step design approach used to develop BUD-MI. Section 3 focuses on the first step, involving systematic identification of key challenges around MI data collection. Section 4 presents the second and third steps, namely the elicitation of requirements along with the systematic mapping of any existing resources (e.g., standards, scientific papers) or the creation of new ones (e.g., international typologies, datasets) to support these requirements. Section 5 integrates all components into the BUD-MI tool and illustrates its functionalities through a case study. Finally, Sect. 6 reflects on the tool’s achievement of its objectives (introduced in Sect. 1.2), discusses its limitations, and identifies directions for future work.

## Method

Because the BUD-MI template is a tool, concepts from the field of design theory were used to ensure its systematic development. The purpose of BUD-MI was clarified using the conceptual design framework proposed by Ralph and Wand (2009). In this framework, the *purpose* of the design object is to offer a response to *problematics* hindering a domain (and its wider research environment) from reaching their *goals* effectively and efficiently (Ralph & Wand, 2009). Figure 1b applies this framework to BUD-MI. Read outside-in, the figure highlights the pivotal roles of domain understanding and problematic identification in formulating BUD-MI’s purpose and objectives. Read inside-out, the framework clarifies the intended contribution of BUD-MI to MS research and its wider research environments (CE and SEM).

With the purpose of BUD-MI clarified, the four iterative steps of the design process were followed: problematic identification, requirement elicitation, mapping of relevant domain constituents (and creation of missing ones), and assembly of these constituents into BUD-MI (Faste & Faste, 2012; Goldsmith, 2004).

### Identification of existing problematics

Identifying existing problematics requires exhaustive domain understanding, and the authors’ cumulative knowledge was complemented with literature on MI and MS research and related fields (e.g., building carbon accounting, building quantity surveying) and with a UK practitioner’s workshop (architects and structural engineers). The many issues surrounding MIs were then grouped into three umbrella problematics (1–3 in Fig. 1b) and translated into three core objectives for BUD-MI (a–c in Fig. 1b).

### Requirement elicitation

In this step, concerns around identified problematics were translated into requirements (i.e., properties BUD-MI should possess). Requirements ranged from specific tasks that users should be able to perform, to technical and regulatory constraints within which BUD-MI should function (e.g., guidelines, standards) (Bjørner, 2006; Sawyer & Kotonya, 2001). Several elicitation techniques were used. Online meetings among authors served as proxy to research-oriented users. An online workshop was convened in the UK to comment on the tool’s prototype, composed of contractors, architects, structural engineers, and sustainable construction consultants. A subsequent prototype was presented to Swedish practitioners (architects, waste managers, and municipal circularity strategists) to collect additional feedback on the tool’s relevance and missing functionalities. Additionally, a total of six research-oriented users (project assistants and PhD students) with varying levels of tacit knowledge provided feedback on the various prototypes (see 2.4).

### Mapping of domain constituents

Domain constituents^1^ are elements of the domain of expertise, such as existing MI databases (e.g., Heeren & Fishman, 2019), standards, tools, guidelines, and typologies. Relevant constituents were mapped from MI and MS literature, international standards on building surveying, environmental modeling (e.g., building LCA), and material and product classifications (e.g., economy-wide MFA). International standards were prioritized since they undergo review, coordination, and harmonization of national standards, making them inherently relevant to Objective 2 (enabling cumulative research). Where suitable constituents were not readily available, they were created for BUD-MI.

### Assembly of constituents

All constituents were assembled into the design object (BUD-MI) that meets all requirements. Several prototypes were generated, tested, and improved based on test users’ feedback (see 2.2). The initial prototype was developed using BIM files provided by UK practitioners. This first prototype was then used by a pilot user to collect MI data from archival building documents (see next paragraph). A subsequent prototype was used to continue collecting data from practitioners in the UK, and users systematically reported issues, unclarities, or missing functionalities. The final prototype was evaluated by four users tasked with collecting nonresidential building MI data. Feedback from all testing stages informed the development of the accompanying user guide (Supplementary Information SI1).

The key capabilities of BUD-MI are demonstrated through the case study that guided most of its iterative development, that is, the MS quantification of the ‘1970s-warehouse’ archetype in an industrial area of Sheffield, UK. Building documentation was obtained from city archives for five representative buildings, resulting in five complete BUD-MI templates. The archetype’s MI was derived as a floor-area-weighted average, and the MS calculated by coupling this MI data to the spatial inventory of the 1970s warehouses within a GIS environment. Also note the use of BUD-MI by Gillott et al. (2025) to explore archetype classification of UK nonresidential buildings and by Liu et al. (2025) to improve the dynamic MFA modeling of Swedish residential building renovations, both further discussed in Sect. 6.1.

## Material intensity: collection, use, and challenges

The knowledge base on MIs is summarized below and later used to identify existing problematics.

### Developing MI data

MIs are typically calculated by compiling the bill of materials for one or several buildings. This process involves recording the material and dimensions of each building item to calculate the total mass (or volume) of material in the building and dividing it by the building size. Common data sources include building blueprints, BIM files, and elucidations using handbooks, regulations, and expert consultations.

An MI dataset embeds four key features. Material categories (e.g., concrete, metals) and the measurement unit (i.e., the unit used to measure material quantities) are tailored to the study’s aim and scope. In contrast, the reference unit (i.e., the unit used to measure the building size) and archetype classification are tailored to the building inventory for which MIs are intended. Archetypes refer to representative buildings whose characteristics (e.g., age, use, location, construction type) are ideally equal to the average of the building stock subset they represent (Loga et al., 2016; Ortlepp et al., 2015). In practice, the bill of materials is conducted for several representative buildings within an archetype, and the MI is calculated as the total mass divided by the total building dimension (in the chosen reference unit). Using archetypes decreases data collection workload as MIs are calculated for a few buildings per archetype rather than all buildings in the stock, but their use introduces uncertainties (Lanau et al., 2019; Zhang et al., 2022).

### Challenges of MI data

MI data presents challenges at several stages, from their collection and formatting to their use and applicability.

#### Data- and labor-intensive

MI data collection is often cited as the main culprit when acknowledging the bottom-up approach’s labor-, time-, and resource-intensiveness (Lanau et al., 2019; Sprecher et al., 2022). In addition to collecting and analyzing building documents, time and effort are required to develop the knowledge base necessary to make informed assumptions about missing information. Because buildings are complicated objects made of many different components, time is also required to organize data collection into a consistent and structured process.

#### Lack of standardization

All four MI features vary throughout literature, precluding their comparison for cumulative research. A few studies provide valuable insights towards a standardized MI format, though none exists yet. Heeren and Fishman (2019) developed an MI database seed that remains, to our knowledge, the most notable effort towards community-based and cumulative research. The authors combed through literature to catalog existing MIs into an open GitHub database and devised a general MI format that accommodates all data. The same year, Schiller et al. (2019) reworked German and Japanese MIs to enable their comparison, concluding that MIs comparability requires the alignment of archetype classification, reference dimension, and material categories. Finally, Zhang et al. (2022) explored which building descriptors are crucial for archetype classification in the context of Chinese buildings and concluded that a building’s structure outweighs the significance of other descriptors (i.e., age, use, and location).

#### Lack of transferability

Transferring MI data refers to using an MI dataset with a building inventory different from the one for which it was developed. Since inventory formats vary across contexts, so do MI formats, thus limiting their transferability. In the best case, MI data are recalculated and reformatted by returning to original data sources to collect missing information. In their study of Germany stock using satellite-based inventories, Haberl et al. (2021) dove back into their previous data collection to recalculate MIs into mass per volume of buildings aboveground, and to reclassify building samples into archetypes better fitting the satellite-based inventory. Alternatively, MIs are recalculated with conversion factors between different building areas (e.g., Nemry and Uihlein, 2008; Gontia et al., 2019), unavoidably introducing a layer of uncertainty to the results. In other cases, the discrepancy between MI and inventory formats is simply mentioned qualitatively as a limitation.

#### Unclear uncertainties

The uncertainties of MI data remain a significant issue for MS research (see Streeck et al., 2025), as the accuracy with which MIs reflect the real-world remains unclear. A few studies address *statistical* uncertainties (i.e., variability within a group of MIs) (Guven et al., 2022; Marinova et al., 2020), but *epistemic* uncertainties arising from data collection are not sufficiently addressed. These pertain to errors during material and dimension takeoff (measurement uncertainties), incomplete or erroneous description of the building (description uncertainties), and density variations across similar construction materials and products (density uncertainties).

#### Inadequate format for the construction industry

Practitioner identified three barriers to using MI (and MS results) as support in circularity strategizing. First, MIs are usually presented in mass of materials per building and lack detail at the building component level (Arora et al., 2019, 2020). Mass units also hinder practitioners from effectively grasping results; dimensional units (e.g., volume of masonry) are more intelligible. Finally, MIs material categories (e.g., metals) often do not align with categories commonly used in the construction industry (e.g., reinforcement steel, structural steel).

### Formulation of BUD-MI objectives

Identifying core problematics helped formulate the three objectives of BUD-MI (Sect. 1.2 and Fig. 1b). Note that challenges of standardization, transferability, and unclear uncertainties are reflected under Objective 2 on cumulative research.

## Requirements and domain constituents

**Fig. 2:**
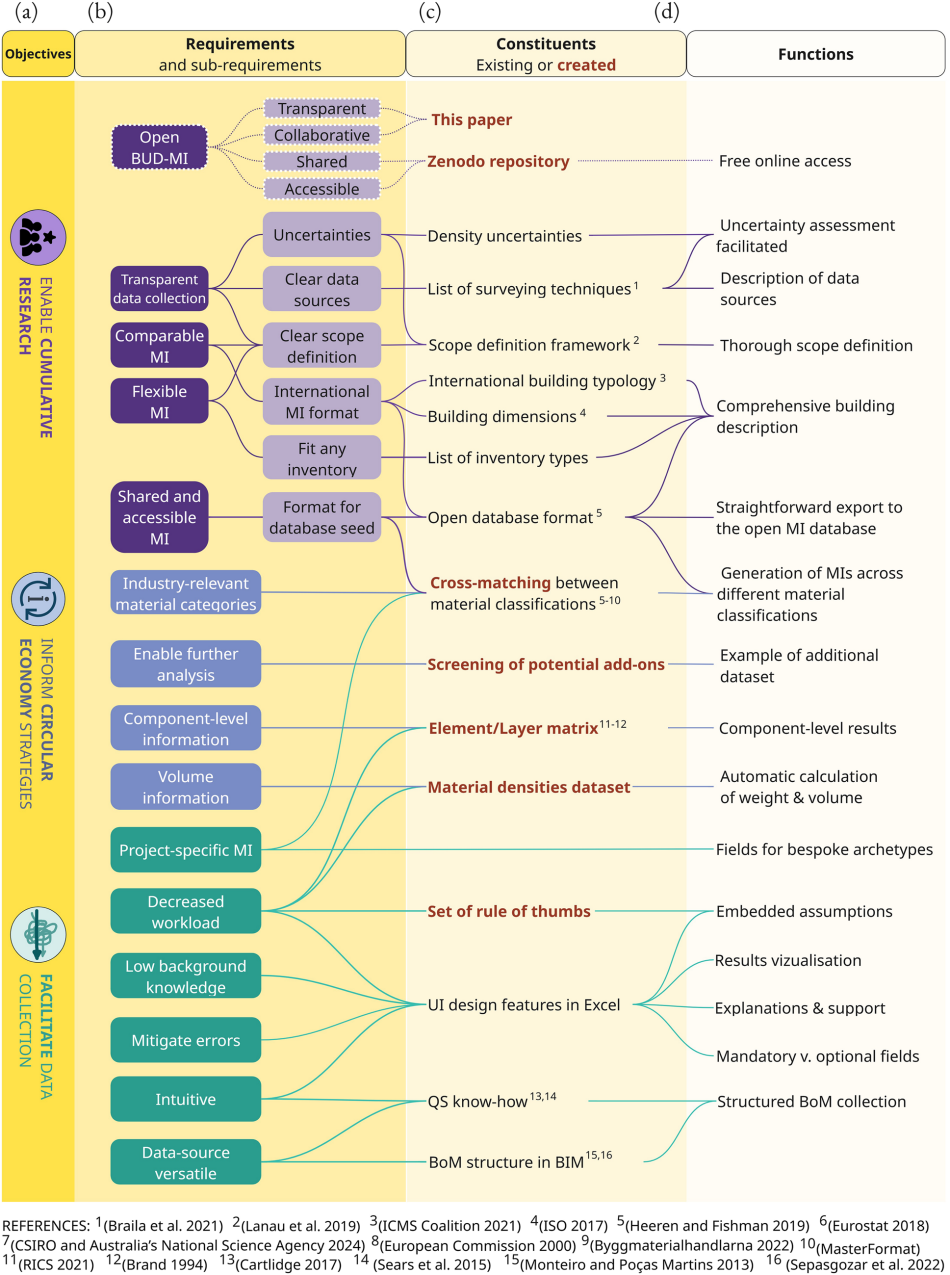
**a** Objectives, **b** elicited requirements, **c** constituents used to build BUD-MI, and **d** functions of BUD-MI that help meet its objectives. Constituents highlighted in red were created for BUD-MI, whether from scratch or as a collection of sources. BIM: Building Information Modeling, BoM: Bill of Material; MI: Material Intensity; QS: Quantity surveying

Figure 2 provides an overview of the requirements elicited for each objective, together with the domain constituents (mapped or created) used to meet these requirements.

### Facilitate the data-collection process

#### Intuitiveness and mitigation of human errors

BUD-MI was developed as a Microsoft Excel document to ensure its accessibility to a wide range of users. Its interface also allowed integrating basic user functions such as searchable drop-down lists, informational pop-ups, automatized calculations, and auto-population of cells, but also result visualizations. So that BUD-MI helps users make relevant assumptions in cases of incomplete or coarse building data, common rules of thumbs used in quantity surveying to quantify construction materials were included (Brooker, 2009; Cobb, 2020).

#### Decreased workload

Several strategies were implemented to decrease the workload of MI data collection. The structure of the bill of materials was based on the tried-and-true data structure used in quantity surveying (Cartlidge, 2017; RICS, 2021a). A density dataset of 404 construction materials (from 267 references) was collected, including linear and areal densities where relevant and available. A three-tier material categorization was also developed to help users find materials quickly. This categorization is a hybrid of function-based (used by architects) and material-based (used by industrial ecologists) classifications. For example, ‘timber boards’ are found in both ‘timber product’ and ‘coverings’ categories. Finally, to manage rising time demands from an expanding template, fields were differentiated between mandatory or optional.

### Support cumulative research

Cumulative research is enabled by open science (Hertwich et al., 2018; Pauliuk et al., 2015) and comparability and transferability of MIs (Sprecher et al., 2022; Streeck et al., 2025). Often bundled together in literature, addressing comparability and transferability require different approaches and are elicited as two separate requirements.

#### Support open science

Open science requires knowledge to be accessible, shared, transparent, and collaboratively developed (Vicente-Saez & Martinez-Fuentes, 2018). The existing open MI database provides a solution for sharing and accessibility of MI data (Heeren & Fishman, 2019). The transparency criterion elicited three requirements (Lederer et al., 2021; Sprecher et al., 2022). First was data source description, for which a list of common data sources was compiled from literature and supplemented with data sources from building reality data capture (e.g., LiDAR) and surveying techniques (e.g., on-site manual survey) (Braila et al., 2021). Second was scope definition, devised for BUD-MI by complementing the MS scope outlined by Lanau et al. (2019) with building-specific considerations (e.g., inclusion of substructure and foundation’s compact layer). Third was uncertainty assessment, focusing on parameter uncertainties from variations in material densities. Where possible, multiple density data points were collected per material to calculate a coefficient of variation; otherwise, a default (modifiable) value of 20% was assigned.

#### Comparability through international MI format

Comparability of MIs requires a consistent reporting format across the four key MI features (reference unit, material categories, unit of measurement, and building typology). To the best of our knowledge, no international MI format exists yet, but MI features were separately explored in literature. For example, reference units are often reported without explicit definition, and Schiller et al. (2019) and Heeren and Fishman (2019) advocate for gross external floor area as the default reference unit, as defined in the now-withdrawn ISO 9836:2017–09, similar to the International Property Measurement Standard (IPMS, 2023) used in the template. Regarding material categorization, Heeren and Fishman’s (2019) was selected since it was developed for MI comparison purposes. As for units of measurement, both mass (kilograms) and volume of materials are used across literature.

For an international MI format, an international buildings archetype classification is required but does not yet exist. Following recommendations of Schiller et al. (2019), a classificaton was developed using building construction method (e.g., timber frame) and building use (e.g., residential) as archetype descriptors, with story count added due to its use across literature (inter alia, Schandl et al., 2020; Wiedenhofer et al., 2024). Descriptor categories are based on the International Cost Management Standard (ICMS), with further disaggregation of the ‘residential’ category to reflect the prevalence of MIs for various types of residential buildings.

#### Transferability through flexible MI format

While an international MI format addresses comparability, it only resolves transferability if building inventories are harmonized globally. Such process would likely reduce modeling resolution. Alternatively, transferability can be achieved by ensuring MIs can be reformatted to fit the inventory at hand. Achieving such flexibility in BUD-MI requires preemptively documenting all relevant building information, including an array of dimension indicators which were compiled from international surveying guidelines (IPMS, 2023; RICS, 2021a; ISO 9836, 2017) but also details specific to new types of building inventories developed with digital techniques. These techniques comprise spectral imaging (e.g., Ye et al., 2017; Zahiri et al., 2021), photogrammetry (Sharma & Garg, 2023), optical and radar satellite data (e.g., Haberl et al., 2021), nighttime light data (e.g., Liang et al., 2023), LiDAR scanning (Schandl et al., 2020), computer vision and machine learning (Arbabi et al., 2022; Raghu et al., 2023), and web-scraping (Mao et al., 2020; Milojevic-Dupont et al., 2023). Using one or a combination of these techniques, reality data is captured about buildings as ‘seen from outside’ such as aboveground building height, or shape and material of façades and roofs, which can be incorporated into new or existing building inventories.

Transferability issues also arise when MI’s material categories do not align with those required for a given study. For example, the category ‘metals’ is too coarse for use in a study of copper stocks in buildings. Several classifications were therefore collated from literature (Table 1) and cross-matched, each with a different purpose or geographic relevance, to facilitate the generation of MIs across various material classifications. The correspondence between building products and materials was not always unequivocal, and the products’ dominant material (in mass) was used as proxy.

**Table 1:** Material classifications used across BUD-MI, including their number of tiers, purpose, and geographic relevance

Material classifications	Tiers	Purpose	Geographic relevance	Source
BUD-MI (input)	3	Facilitate material selection during data collection	Global	*This article*
BUD-MI (result summary)	1	Summarize MI results for quick assessment		*This article*
Open MI database	4	Share and compare MI data		Heeren and Fishman (2019)
Economy-wide MFA	3	Enable the use of MIs with existing MFA frameworks		Eurostat (2018)
Global MFA	2			International Resource Panel (2024)
Industry-oriented	3	Facilitate sharing of results with construction actors	Europe/Sweden	Byggmaterialhandlarna (2022)
MasterFormat, simplified	2		North America	RSMeans data (2024); MasterFormat (n.d.)
European List of Waste (European Waste Codes)	2	Support waste management considerations	Europe	European Commission (2000)

*MFA*: Material flow analysis, *MI*: Material intensity

### Informing circular economy strategies

As mentioned in Sect. 3.2, practitioners outlined several criteria for MIs to better assist them in strategizing a CE. They advocated for better alignment between their information needs and MI’s material categories, leading to the inclusion of several industry-tailored material classifications in BUD-MI (Table 1).

Stakeholders also corroborated their need for a component-level understanding of buildings to inform reuse operations (Arora et al., 2020), and a differentiation between super- and sub-structure—the latter being less accessible for recovery operations. To achieve this, using a building classification system in BUD-MI would be relevant but none is systematically used internationally (BuildingSMART, 2018). Their elevated level of detail (typically five-tier) is also challenging, as such detailed classification systems would increase data collection time, thus defeating Objective 1. Additionally, construction documents of older buildings (main targets of circularity strategies) rarely contain such detailed specifications.

**Fig. 3:**
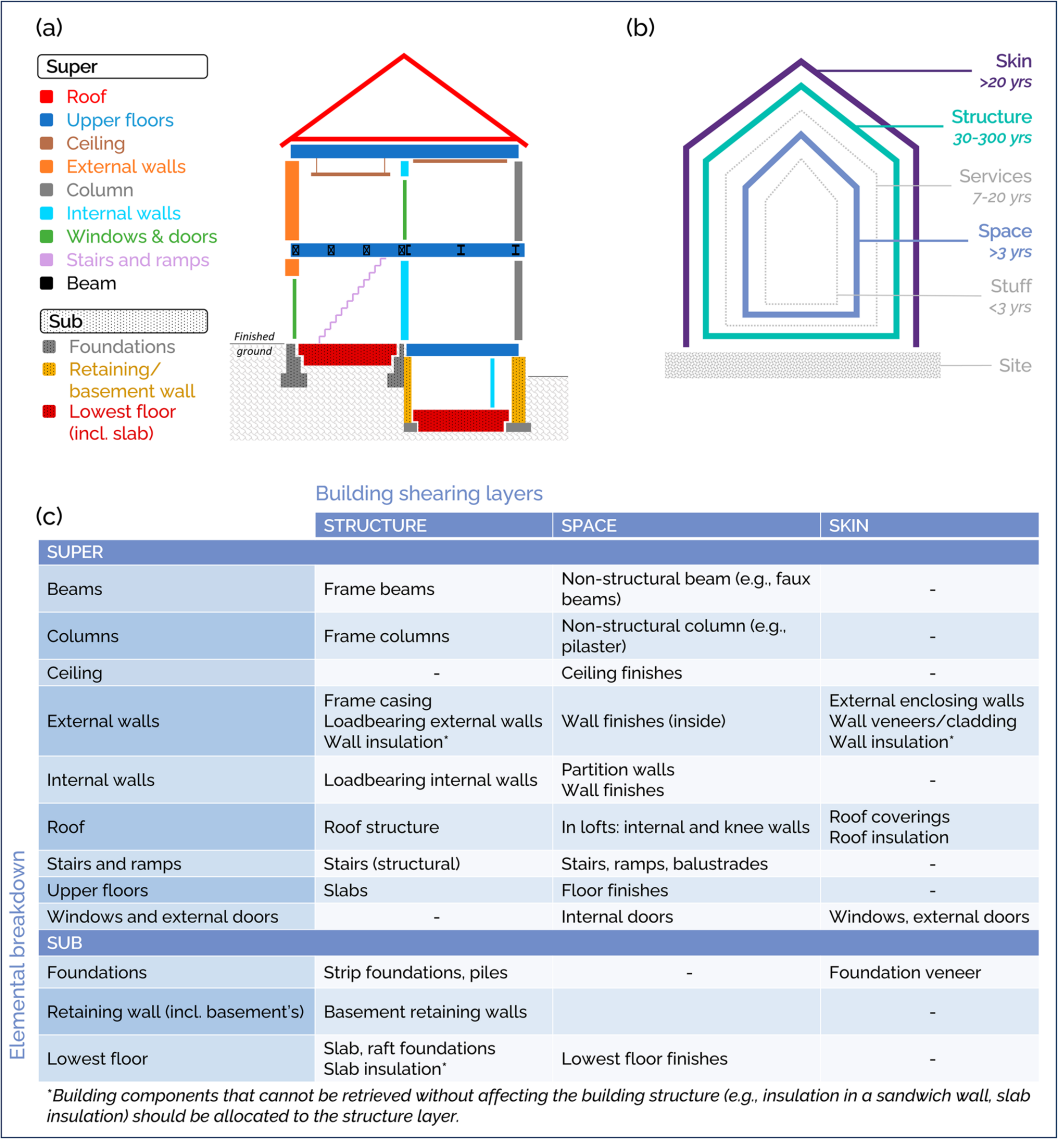
**a** elemental classification of building components used in BUD-MI, adapted from the New Rules of Measurement 1 (RICS, 2021b), **b** Building shearing layers (adapted from Brand, 1994), and **c** how crossing this information achieves a near-component-level understanding of a building

To balance all requirements, two common building breakdowns—elemental breakdown (Fig. 3a) and building shearing layers (Fig. 3b)—were combined to reach a near component-level understanding of a building (Fig. 3c).

#### Elemental classification

Commonly used in quantity surveying, the elemental classification decomposes a building into its elements (e.g., foundations, external walls). Integrating this classification in BUD-MI ensures that MIs can be generated for each building element (i.e., elemental MIs, such as kilograms of bricks per square meter of wall). These elemental MIs can be used to parametrize MS modeling and better capture the influence of building geometry on material quantities (Stephan & Athanassiadis, 2017). Many different elemental breakdowns are used across the world (CEEC, 2014; Smith, 2016) and the global ICMS standard is too aggregated for BUD-MI’s purpose (ICMS Coalition, 2021).

Therefore, the elemental breakdown of the New Rules of Measurements 1 (RICS, 2021a), developed for worldwide application, was adapted. This breakdown achieves sufficient level of details (Objective 3) while not overburdening data collection (Objective 1). The terminology was adjusted by differentiating vertical location between ‘super’ and ‘sub’, reflecting the common differentiation between aboveground and underground MS in research while remaining consistent with construction practice terminology.

#### Building shearing layers

The concept of building shearing layers decomposes a building into six layers based on their function (Brand, 1994; Duffy, 1992). Shearing layers are used in circularity assessment tools such as GPR Gebouw (Netherlands) and Regenerate (UK) (Gillott et al., 2023; GPR, 2023). Indeed, as each layer is tied to an average lifetime, the concept is particularly relevant to circularity considerations. For example, materials in the ‘structure’ layer remain in use for as long as the building, while materials in the ‘space’ layer are replaced more frequently.

#### Towards component-level material intensity data

Crossing information from the two building breakdowns enables reaching a near-component level understanding of the building (Fig. 3c) with crucial circularity considerations. For example, an item tagged as ‘internal wall’ (element) and ‘space’ (shearing layer) refers to a partition wall, while an item tagged as ‘internal wall’ and ‘structure’ refers to a load-bearing internal wall. The latter is likely to stay in use much longer than the former.

## Architecture and functionalities of BUD-MI

**Fig. 4:**
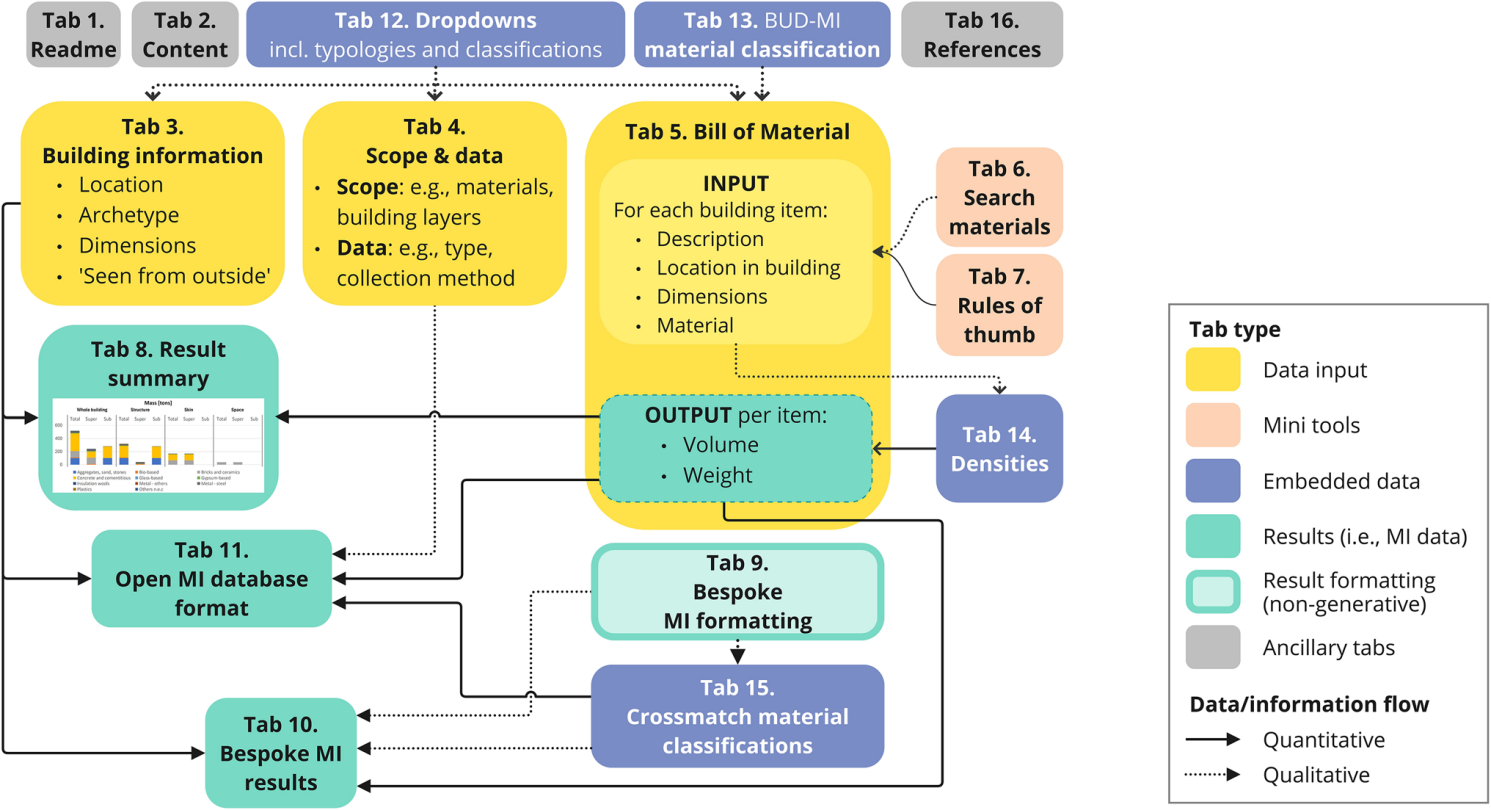
Architecture of BUD-MI, visualizing the information flow across the different tabs. Note the lighter color of tab 9, signaling that the tab pertains to results (users specify their preferred MI format) but does not generate any

The architecture of BUD-MI, depicted in Fig. 4, comprises three data input tabs, two mini-tools tabs, four result-generation tabs (each with a different purpose), and additional tabs for background data and ancillary information. BUD-MI (version 1.0.0) is available on GitHub (github.com/ML-IE/BUD-MI), including a readme document, a change log document, a fully filled BUD-MI template as example, and the user guide.

### Data input tabs

**Fig. 5:**
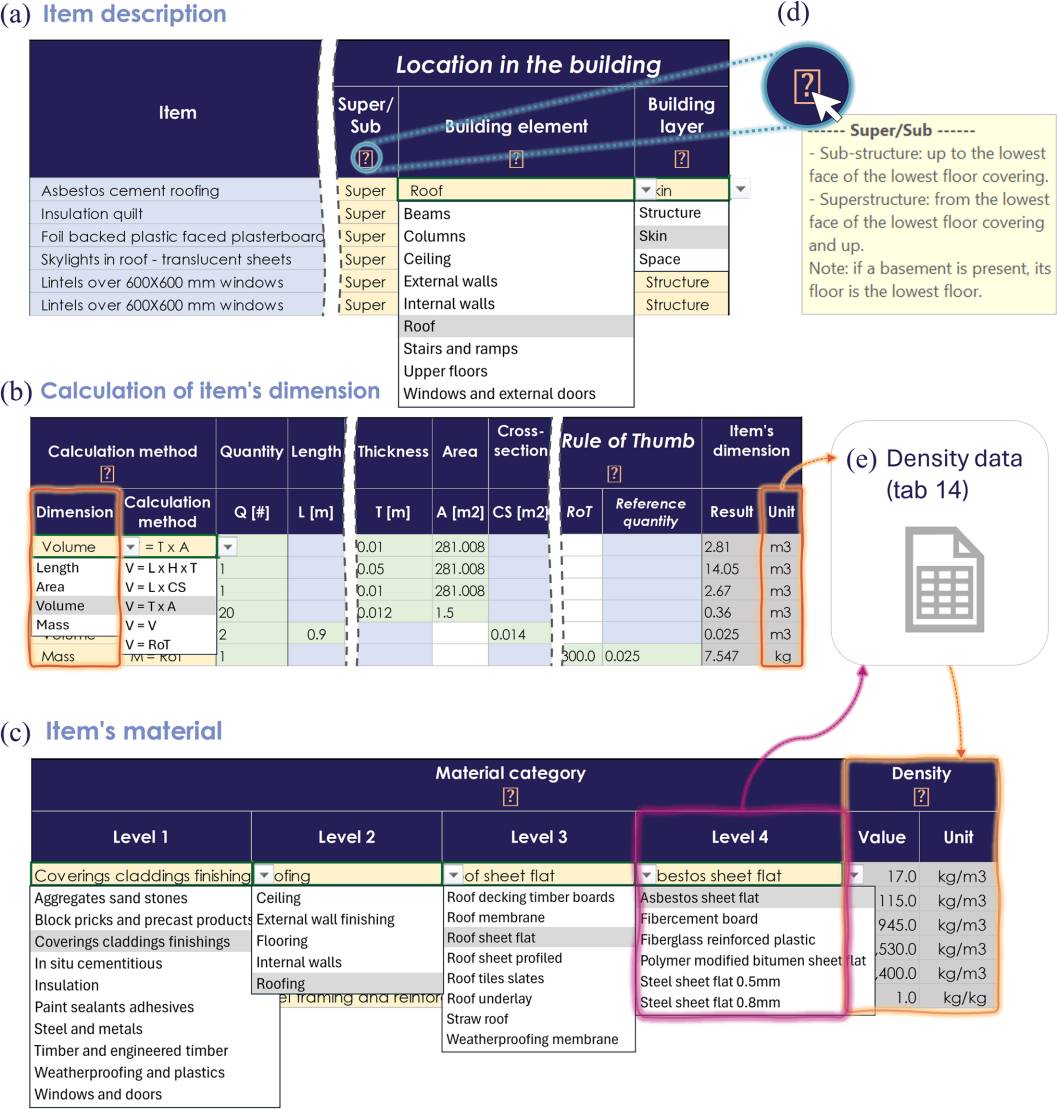
In the tab ‘Bill of Material’ (Tab 5), users **a** describe each building’s item and specify its location in the building using dropdowns, **b** choose how to, and calculate the item’s dimension, and **c** specify its material. **d** The informative pop ups support users during data collection. **e** The item’s dimension (**b**) and material (**c**) are used to locate the relevant density in the ‘Density data’ tab (tab 14)

Basic user interface features are included across all data input tabs, including searchable dropdowns and informational pop-ups (Fig. 5d). Color coding differentiates manual input cells, dropdown selection, and auto-populated fields, as well as mandatory and optional fields.

#### Building description

In tab 3, users enter the building’s location, archetypical features, main dimensions, and information on the building ‘seen from outside’. An additional section captures information required for the open MI database.

#### Scope and data

In tab 4, users define the scope of data collection, specifying the materials and buildings parts under analysis. In the example study, all materials and shearing layers are included, along with both super- and sub-portions of the building, but the foundation’s compact layer is excluded. Users also state the type of data sources (e.g., construction documents, BIM data) and their scope (e.g., architectural, structural). Finally, users record their name and data collection progress.

#### Bill of materials

The bill of materials (tab 5) is the core data input of the template, where users specify the dimensions and material of each item in the building across four distinct segments. In ‘Item description,’ users qualitatively describe each item and locate it across shearing layer and building element (Fig. 5a). Users then select the targeted ‘Item’s dimension’ and enter the item’s measurements in highlighted fields (Fig. 5b). ‘Item’s material’ is selected through nested dropdowns reflecting BUD-MI's material classification (Fig. 5c); the ‘Search material’ mini-tool (see 5.2) is useful at this stage. Should the material not be listed, users can add it and its density manually (as described in the user guide). The item’s mass and volume are then automatically calculated based on the entered dimension and material, with the density retrieved from tab 13) (Fig. 5e).

### Mini-tools and embedded datasets

Two mini-tools help users during data collection. ‘Search material’ (tab 6) displays the material classification used in BUD-MI (tab 5), and users can locate a specific material’s category using the filter or search functions. Common material synonyms are included to address nomenclature variations across countries. In ‘Rules of Thumb,’ (tab 7) users can approximate quantities based on available data (e.g., share of mortar in brick and block masonries, or length of studs in a drywall based on its area).

Embedded datasets include all lists (e.g., countries) and typologies (e.g., building use) used for dropdown lists (tab 12), BUD-MI’s material classification (tab 13), all material densities and their uncertainty (tab 14), and crossmatches between material classification (tab 15) to support the generation of MIs in various formats.

### Resulting MIs

**Fig. 6:**
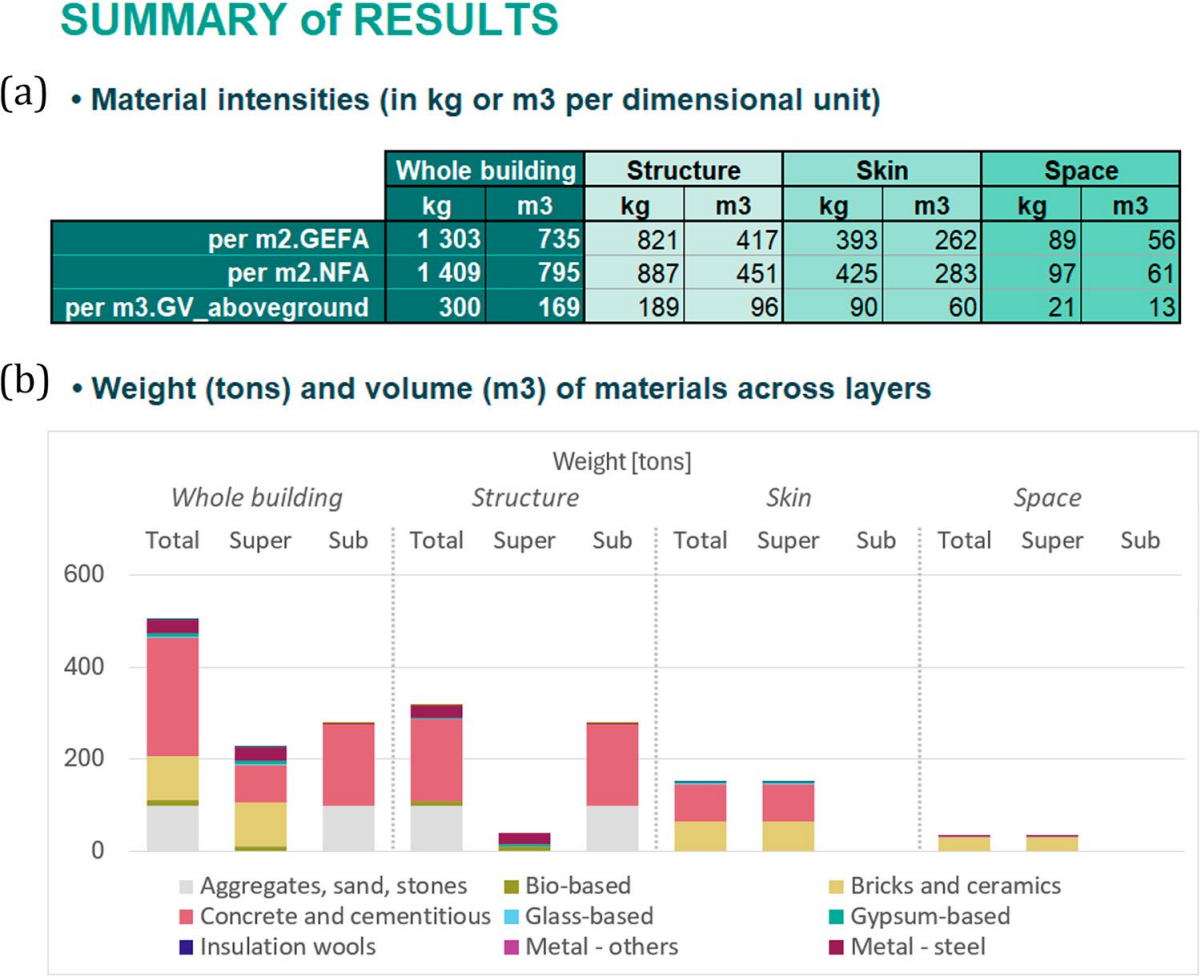
Excerpt of the tab ‘Summary of results’ (tab 8). **a** Total MIs are displayed in various units. **b** Total mass and volume of materials are displayed as graphs and tabular data, disaggregated across shearing layers and material categories. Note that tabular data and volume results are not displayed here but indeed available in BUD-MI. For underlying data, see the Data Availability statement

In tab 8, ‘Result Summary’, a result overview is displayed in mass and volume across shearing layers and main material categories (Fig. 6a). The bar chart visualization allows users to sense-check results and spot aberrant values pointing to data collection errors. In tab 11, ‘Open MI database format’, results are generated in a format suitable for export and upload to the open MI database.

**Fig. 7:**
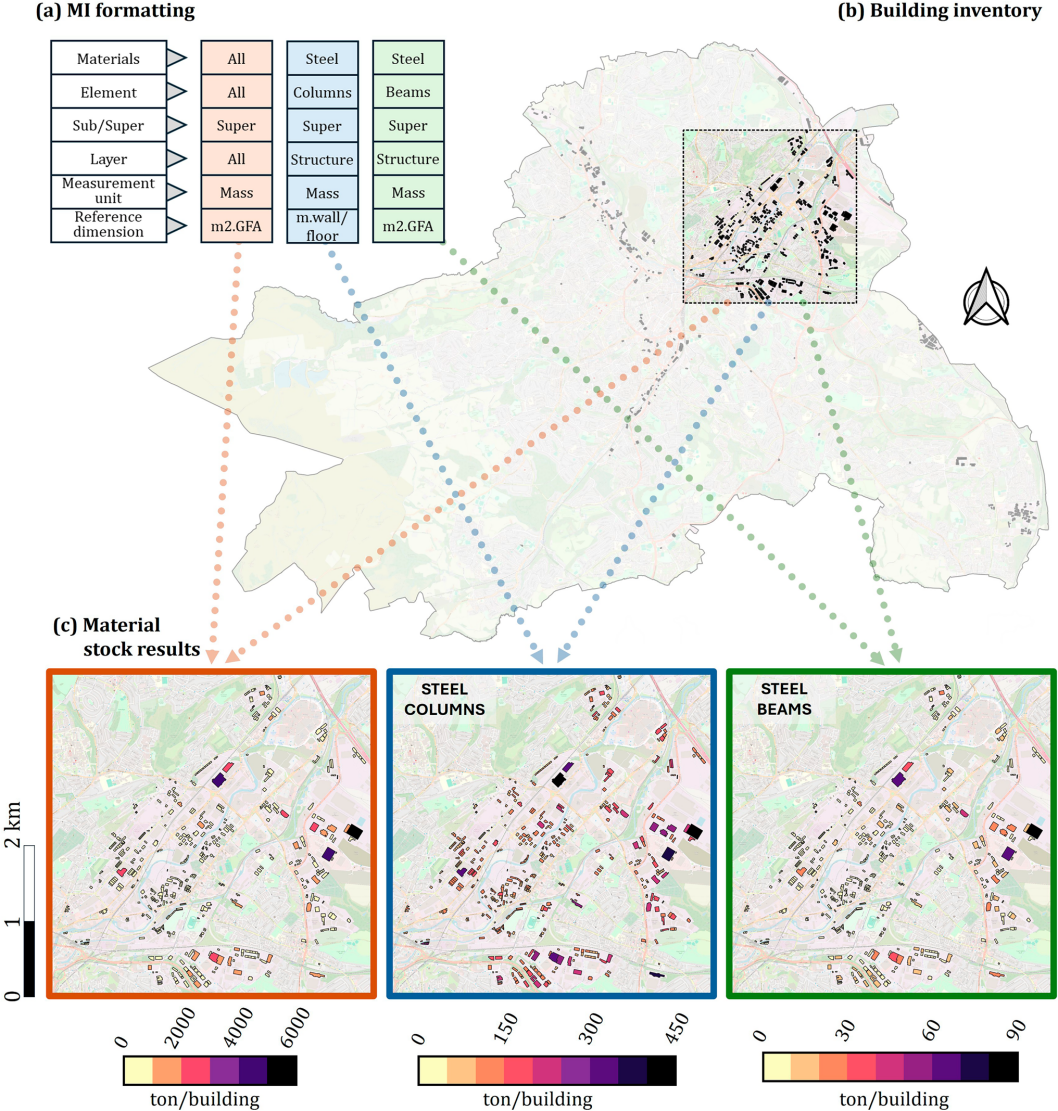
The ‘bespoke formatting’ functionality of BUD-MI and the modeling capabilities it offers. **a** MIs can be generated in different formats and units, by selecting specific materials, building elements, vertical delineation, shearing layer, and the desired measurement unit and reference dimension. **b** Combining these MIs with the area’s building inventory (here, a neighborhood in Sheffield, UK) enables modeling of **c** different material stock results with above ground total material stock (left), steel beam stock (middle), and steel column stock (right); note the difference in scale. For underlying data, see the Data Availability statement

In tab 9, ‘Settings for bespoke MI format’, users select preferences for MIs formatting, including reference dimension, unit of measurement, material classification, and which shearing layers and elements to include. The formatted MIs are then generated accordingly in tab 10, ‘Bespoke MI results’. In the example study, three sets of MIs were generated (Fig. 7b). The first reflects the total mass of construction materials in the ‘super’ portion of industrial buildings. A structural-steel-beam MI and a structural-steel-column MI were also generated, using gross floor area and building perimeter as reference dimensions, respectively. These three sets of MIs were each coupled to the area’s spatial inventory in a GIS environment (Fig. 7b), resulting in the total MS of construction material, structural steel columns stock, and structural steel beams stock (Fig. 7c).

## Discussion and conclusion

This paper presents the development of the BUD-MI data collection template towards three objectives: to streamline MI data collection (Objective 1) and generate MIs that support cumulative research (Objective 2) and that better support construction practitioners in strategizing for CE (Objective 3). The novelty lies in synthesizing and operationalizing existing approaches by integrating disparate standards and concepts into a streamlined workflow. The work also contributes four new resources to the field: a density dataset of over 400 building materials (including standard deviations where relevant), an international building archetype format, a crossmatch of seven material classifications, and a framework for efficiently achieving near-component-level understanding of buildings.

Read inside out, Fig. 1 illustrates BUD-MI’s intended contribution to the MS research domain and its wider environments (CE and SEM research), and the discussion below follows this widening logic. First, the extent to which BUD-MI addresses the identified challenges is discussed, differentiating between direct contribution (Sect. 6.1) and indirect contribution that requires the resolution of additional factors and thus reflect limitations of this work (Sect. 6.2). Next, Sect. 6.3 positions BUD-MI within the broader research context through comparison and complementarity with existing tools and framework of MS research. Finally, Sect. 6.4 outlines future work and provides an outlook that concludes the paper.

### Direct contributions

BUD-MI directly addresses several domain challenges. Under Objective 1, as reported by users, considerable time is saved during data collection and learning in MI data collection and MS research was accelerated by informational pop-ups and the user guide.

Under Objective 2, BUD-MI is available on GitHub to allow for transparency, reproducibility, and community-based development. Its value for cumulative research was demonstrated in several studies. It facilitated post-collection research inquiries on MIs: using data from 30 UK buildings of diverse structures and uses, Gillott et al. (2025) could examine whether building use or construction type (both captured in Tab 3) better predicts a building’s material content and embodied carbon. In the CREATE project, the ‘bespoke formatting’ function (tab 9) harmonized two disparate MI datasets (Gontia et al., 2018; Lederer et al., 2021) into a unified format for integration into a multi-city digital material cadaster (CREATE, 2024). In another study, decomposing MIs across shearing layers (tab 5) allowed refined dynamic MFA modeling of renovation activities by integrating lifetime convolutions between structure, skin, and space layers (Liu et al., 2025).

Under Objective 3, the near-component granularity of MIs supported subsequent MS modeling at the same level of detail. Construction stakeholders (including municipal circularity strategists, waste management companies, and circularity architects) confirmed that MS results with such granularity could bolster strategizing around circularity concepts like ‘design with blanks’ and ‘form follows availability’, in which built assets are designed based on available secondary resources available (Gang, 2010; Josefsson & Thuvander, 2020; Lanau et al., 2024).

### Indirect contributions and limitations

Three key decisions made during the development of BUD-MI also reflect its limitations. First, despite ensuring accessibility, the use of Excel spreadsheets introduces issues of data integrity and stability. Consequently, test users tended to overly rely on the template’s embedded data even when adding material and density information would have improved results, due to concerns about affecting the template’s functionalities.

Second, BUD-MI was designed to be country-agnostic to ensure broad accessibility. This choice limits the inclusion of a myriad of functionalities that rely on context-specific data. For example, each building material could be supplemented with end-of-life management options, embodied carbon values, or cost factors. Additional industry-relevant and country-specific material categorizations (e.g., Uniformat in North America) could be added to provide even greater value to stakeholders. Additionally, users should verify that the rules of thumb incorporated in BUD-MI are relevant to their case and if not, should adapt them to local practices.

Third, BUD-MI facilitates*—*but does not fully address—the quantification of density and measurement uncertainties. The coefficient of variation for each material density enables Monte Carlo propagation of density uncertainties, though it must be performed externally as it is unsupported by the spreadsheet format. Measurement uncertainties may be inferred from the data sources declared in tab 4 ‘Scope and Data’. For example, measurements from scanned hand-drawn plans likely carry greater uncertainty than those automatically extracted from BIM files. Description uncertainties are not covered by BUD-MI, but the pedigree matrix developed by Laner et al. (2014) for MFA uncertainties and partly adapted to MIs by Guven et al. (2022) is promising. Still, overall, an integrated framework accounting for all MI uncertainties remains lacking.

Additional limitations arise from the scope of this work, as several challenges extend beyond MI-specific concerns and require further efforts. For example, the scope definition (tab 3) and building information (tab 4) frameworks support cumulative research but full realization of open-science practices and data transferability depends on infrastructure for storing and sharing of BUD-MI results. The open MI database offers one avenue—supported by BUD-MI’s automatic formatting into the database format (tab 11). However, its typologies and fields are too coarse to accommodate the various existing MIs, as only parts of BUD-MI’s collected information can be uploaded and key information for MI transferability are missing. Alternatively, a new database could be created; the planned development of BUD-MI into a web-based interface (Sect. 6.4) provides this opportunity since a thoroughly structured backend database will be required.

Finally, in the bottom-up MS modeling workflow, BUD-MI operates at the MI data collection stage (Fig. 1a), but preceding and subsequent steps also affect MS results. During archetype classification, variability within archetypes should be minimized but the number of archetypes must remain manageable and averaging introduces unavoidable uncertainties (Ortlepp et al., 2015). Sample selection also directly affects statistical reliability, emphasizing the need for representative samples and error quantification (see Gy’s (1992) theory of sampling). Building sample documents are then analyzed with BUD-MI to produce sample-MIs that are averaged into archetypical MIs outside the tool. These archetypical MIs are finally integrated into spatially explicit inventories within external modeling environments (e.g., geographical information system) to generate MS models, with the application scale depending on archetype representativeness. In summary, BUD-MI provides a structured approach for MI data collection that supports the overall bottom-up MS modeling workflow, but the quality of final MS results depends on careful implementation of all stages.

### Positioning within the research landscape

Situating BUD-MI within the expanding landscape of MS and CE research methods and tools clarifies its role and highlights potential synergies.

To start with, the increasing number of sources of building data (e.g., BIM data, digital material passports) can be capitalized on by developing automatic data exports, for example with visual programming platforms like Dynamo BIM (Autodesk Inc., 2021). BUD-MI’s functionalities could also be expanded to support additional industrial ecology methods. For instance, generating MIs in terms of raw materials requirement (i.e., how much of which raw material is required for one kilogram of concrete) would benefit MFA modeling and support resource management research. Much of the data needed for such ‘raw-MIs’ is already available from IOER-ISBE (2024) in Germany.

Additionally, circularity potential assessment tools such as Regenerate (Gillott et al., 2023) can be coupled to BUD-MI results to evaluate the circularity potential of building stocks. Another framework is Nested Phoenix, which models and improves the life cycle environmental performance of built stocks across scales (Stephan et al., 2022). The framework acts as a container whose results depend on input data quality, and aligning BUD-MI outputs with its input requirements would enhance synergies. For example, Slavkovic et al. (2022) developed a classification of ‘built asset types’ for Nested Phoenix which could be specified in the ‘Archetype – Bespoke’ field of BUD-MI. At minimum, such alignment would allow validation of Nested Phoenix material data.

New digital techniques developed to create or complement building inventories with new building information also offer potential synergies. As additional attributes become available, BUD-MI files can be used to explore the robustness of various archetype classifications and increase modeling reliability. Indeed, BUD-MI’s wide range of recorded building attributes (Tab. 3 ’Seen from outside’) enables exploration of the relevance of various archetype descriptors. For example, computer vision can identify external wall materials (e.g., stucco). In practice, all BUD-MI files with ‘stucco’ as external wall material could be aggregated into a ‘stucco-wall MI’ for which MI variations could be assessed.

### Future work and outlook

The main planned development pertains to developing BUD-MI into a web interface with the same data entry forms, and complemented with automated validation (through e.g., allowable value ranges) and statements of data confidence levels. Careful consideration of ethical and data governance aspects will be required during the interface development, especially with regards to the MI database that the interface would be coupled with. This MI database will not only require secure storage, but also clear statements from data collectors and owners with the inclusion of opt-out functions for any users not willing to share their data. Until such interface is developed, sharing detailed MI results remains highly beneficial to the research community and the authors encourage users to include their MI results in the supporting information of their publications.

This article set out to develop an MI data collection template that maximizes the utility of MIs given the time and resources allocated to their collection. The result of this work is the BUD-MI template, which fulfilled its purpose in the few research projects in which it is used. BUD-MI is available on GitHub and prospective users are encouraged to download and assess its potential usefulness firsthand, and to suggest additions and improvements. Used in its simplest form, BUD-MI is valuable for users to familiarize themselves with MI data collection and the array of considerations to bear in mind to maximize the yield of their data collection efforts. It also makes for an accessible engagement tool for practitioners. Users are welcome to tailor it to their specific analytical needs by adding functionalities; however, complementing rather than replacing existing features should be prioritized to maintain the internationalization and harmonization efforts central to BUD-MI’s design. The authors envision BUD-MI supporting current and future efforts in MI data collection, while ensuring MI data quality, granularity, comparability, transferability, and availability. The resulting MIs will support SEM and CE research and practices in unlocking new analytical and practical possibilities to better understand and manage building material stocks.

## Supplementary Information

Below is the link to the electronic supplementary material.

Supplementary file1 (PDF 9759 KB)

## Data Availability

The data and outputs that support the findings of this study are openly available on GitHub at https://github.com/ML-IE/BUD-MI. This includes data for Fig. 6, which can be found in the file “BUD-MI_example.xlsx”, under the tab “Results—Summary”. Data for Fig. 7 cannot be shared due to sharing restrictions on the underlying spatial inventory.

## Abbreviations

BIM: Building information modeling
CE: Circular economy
MFA: Material flow analysis
MI: Material intensity
MS: Material stock(s)
SEM: Socio-economic metabolism

## Footnotes

1 The standard design terminology is domain “components.” It was changed to domain “constituents” to avoid confusion with building “components” throughout this manuscript.
