# Supplementary material for: BUD-MI: a template that optimizes material intensity data collection and utilization
Source: J Ind Ecol. 2026 Mar 13;30(2):631–49. doi: 10.1007/s44498-026-00044-w (PMC13226353; doi:10.1007/s44498-026-00044-w)
Supplement: Supplementary file 1 — Supplementary file1 (PDF 9759 KB) [file 44498_2026_44_MOESM1_ESM.pdf]

BUD-MI

(Bottom-Up Data: Material Intensity)

User Guide

CHALMERS

The University Of Sheffield.

Maud Lanau. (2025). BUD-MI User guide (v0). Available online at https://github.com/ML-IE/BUD-MI. Last updated on the 3rd of November 2025.

Unless stated otherwise, all illustrations were drawn by Maud Lanau.

About this user guide

This user guide is intended to help users of BUD-MI.

The latest version (and earlier ones) of BUD-MI can be found at: https://github.com/ML-IE/BUD-MI

The scientific article on the background and development of BUD-MI here: https://doi.org/10.5281/zenodo.12722009

Planned developments include tutorial videos about using BUD-MI, a data repository for users to upload their results on a voluntary basis, and – in the longer run – an online interface of the tool.

Contact information.

Maud Lanau

Department of Architecture and Civil Engineering
Chalmers University of Technology, Gothenburg, Sweden

maud.lanau@chalmers.se

Acknowledgements

Maud Lanau (ML) thanks all those who contributed to the development of BUD-MI. These include the co-authors of the scientific publication: Dr Charles Gillott C, Dr Will Mikhelsen, Dr Kimberlee Zamora, Dr Peter Berrill, Dr Niko Heeren, Dr Georg Schiller, Dr Ruichang Mao, Dr Mohit Arora, Dr Karin Gruhler, Prof. Gang Liu, Prof. Hiroki Tanikawa, and Prof. Danielle Densley Tingley. ML thanks all colleagues and students who tested various versions of BUD-MI and provided feedback: Martin Christiansson, Pak Yin Lee, Danielle Abbey, Helena Krantz, and Qiyu Liu. ML also thanks Taz Lodder for advice and guidance on open data sharing and storage. Finally, ML thanks all practitioners for attending the workshops and answering questions, as well as Sheffield City archives and Gothenburg City archives for providing the building documents used for testing and improving BUD-MI.

Table of contents

INTRODUCTION 1

1. **Terminology and acronyms** 2
2. **Architecture of BUD-MI** 4
3. **General information and tips** 5
   3.1. Units 5
   3.2. User interaction features 5
   3.3. Modifying & complementing BUD-MI 6

CHAPTER I. DATA INPUT 7

1. **Building Information** 8
   1.1. Building Location 9
   1.2. Archetypical information 9
   1.3. Project information 11
   1.4. Dimensional information 12
   1.5. 'Seen from outside' 15
   1.6. Building foundations 28
   1.7. Database seed information 29
2. **Scope of study & data description** 30
   2.1. Scope of study 31
   2.2. Data collection 31
   2.3. Status of analysis 34
3. **Bill of Material** 35
   3.1. Item's description 36
   3.2. Calculation of item's dimension 40
   3.3. Item's material 45
   3.4. Item's weight and volume 46

CHAPTER II. MINI TOOLS 47

1. **Rules of Thumb** 49
2. **Search for Materials** 51

CHAPTER III. UNDERSTANDING & GENERATING RESULTS 53

1. **Results - Summary** 54
2. **Bespoke MI Format - Settings & Results** 56
   2.1. Bespoke MI format - settings 56
   2.2. Bespoke MI format – results 58
3. **Results - Open MI DB** 59

CHAPTER IV. SUPPORTING DATA 61

1. **Quantitative Data** 62
   1.1. Densities 62

2. Qualitative Data **62**
2.1. BUD-MI core material classification 62
2.2. Crossmatch material classifications 62
2.3. Dropdowns 62

CHAPTER V. MODIFYING & COMPLEMENTING BUD-MI **65**

1. Adding Materials to BUD-MI **66**
1.1. Adding materials to background data 66
1.2. On-the-fly 69

2. Adding a Material Classification **71**

REFERENCES **73**

ANNEX **i**

1. BUD-MI material classification **ii**

2. Result summary **xvii**

3. Economy-wide Material Flow Analysis **xviii**

4. Global Material Flow Analysis **xx**

5. Open Material Intensity Database Seed **xxi**

6. European Union List of Waste **xxii**

7. Industry-oriented Material Classification **xxiv**

INTRODUCTION

This user guide aims to assist users in understanding and using BUD-MI. It also explains how to modify BUD-MI. Additionally, and while all efforts were made for BUD-MI to be intuitive, we include a non-exhaustive knowledge base as a resource for BUD-MI users to consult.

Below, the terminology and acronyms used in BUD-MI are described, together with the tool's architecture and list of tabs. Some general information and tips are given as well.

1. Terminology and acronyms

Table 1 Terminology used in this user guide. Note that some terms are contextualized to material stock and circular economy research.

Term	Explanation
Apparent density	Mass of a material per unit dimension, including any void spaces or pores within the material. Also called "bulk density".
Archetype	Archetypes are representative buildings. They represent a group of buildings with similar parameters (i.e., archetype descriptor), such as construction period, geographical area, building typology, building structure, and more.
Bespoke	Tailored for a specific purpose.
Bottom-up approach	Bottom-up approach for material stock modelling, using the general equation "MS = INV x MI", where MS is material stock, INV is the inventory of items under study, and MI is the material intensity of each type of item under study.
Bulk density	See "Apparent density".
Building	Construction with a cover and enclosure to house people, equipment, or goods. (ICMS Coalition, 2021)
Database seed	The open database of Material Intensities started by (Heeren & Fishman, 2019) https://github.com/nheeren/material_intensity_db
Inventory	Inventory of buildings in a specific geographical boundary.
Material stock (MS)	Total amount of construction material accumulated in the form of buildings, infrastructure, and long-lived products, for a defined system (e.g., a region, a country) and at a specific point in time.
Quantification unit	The unit used to express the amount of material being measured, expressed in mass or volume.
Quantity surveying (QS)	Discipline within the construction industry which focuses on managing project costs and budgets. It involves the measurement, estimation, and management of materials, labour, and other resources required to complete a construction project.
Rule of thumb (RoT)	A practical procedure, approach, or principle to measuring, calculating, or doing something approximately, based on experience or practice.

Sample building	The building for which the data collection is performed.
Unit of measurement	The dimensional metric that describes the size of the building. Typically, the unit of measurement is an area (e.g., net floor area, gross floor external areas) or a volume (e.g., cubic meters aboveground).
"The template"	BUD-MI
Vertical scope	The extent of the study regarding the vertical dimension of the building. The vertical scope specifies whether the focus includes the sub-structure, the super-structure, or both.

Table 2 Acronyms used in this user guide and in BUD-MI.

Acronym	Full form
A	Area
BoM	Bill of Material
BUD-MI	Bottom-up Data: Material Intensity data collection template
CE	Circular economy
CS	Cross Section
H	Height
ICMS coalition	International Cost Management Standard Coalition
IE	Industrial ecology
INV	Inventory
kg	Kilograms
L	Length
m, m^2, m^3	Meter, square meter, cubic meter
M	Mass
MS	Material stock
Q	Quantity
QS	Quantity surveying
RoT	Rule of thumb.
SEM	Socioeconomic metabolism
t	Metric ton (=1000 kg)
T	Thickness
V	Volume

2. ARCHITECTURE OF BUD-MI

BUD-MI contains 16 tabs, organized in five groups as shown in Table 3 below.

Table 3 Tabs of BUD-MI.

Tab type	Tab #	Tab name	Description
Ancillary	1	About	Background information
	2	Content & Good-to-know	Table of content & tips
Input tabs	3	Building information	Where the building is described.
	4	Scope & Data	Where the scope and data sources are defined.
	5	Bill of Material	Where the bulk of the data collection happens.
Mini tools	6	Search materials	Find various materials and how they are categorized in Tab 5.
	7	Rules of thumb	Various assumptions that may help in case of missing data.
Result tabs	8	Result summary	An overview of results – helpful to spot any blatant error from data collection.
	9	Bespoke MI formatting	Where the format of the MI can be changed (in terms of units, and which building part to include).
	10	Bespoke MI results	Where MI results are displayed following the formatting chosen in Tab 9.
	11	'Open MI database' format	MI results in the format of the 'Open MI database' (as of 2023).
Background data	12	Dropdown lists	All dropdown lists used in BUD-MI.
	13	BUD-MI Material classification	The material classification used in Tab 4.
	14	Materials densities	Density of various construction materials.
	15	Crossmatch across material classifications	Crossmatch between BUD-MI material classification (Tab 13) and other common classification used in industrial ecology, material flow analysis, and in the construction industry,
Ancillary	16	References	References

Figure 1 below shows the interaction between the 16 tabs of BUD-MI.

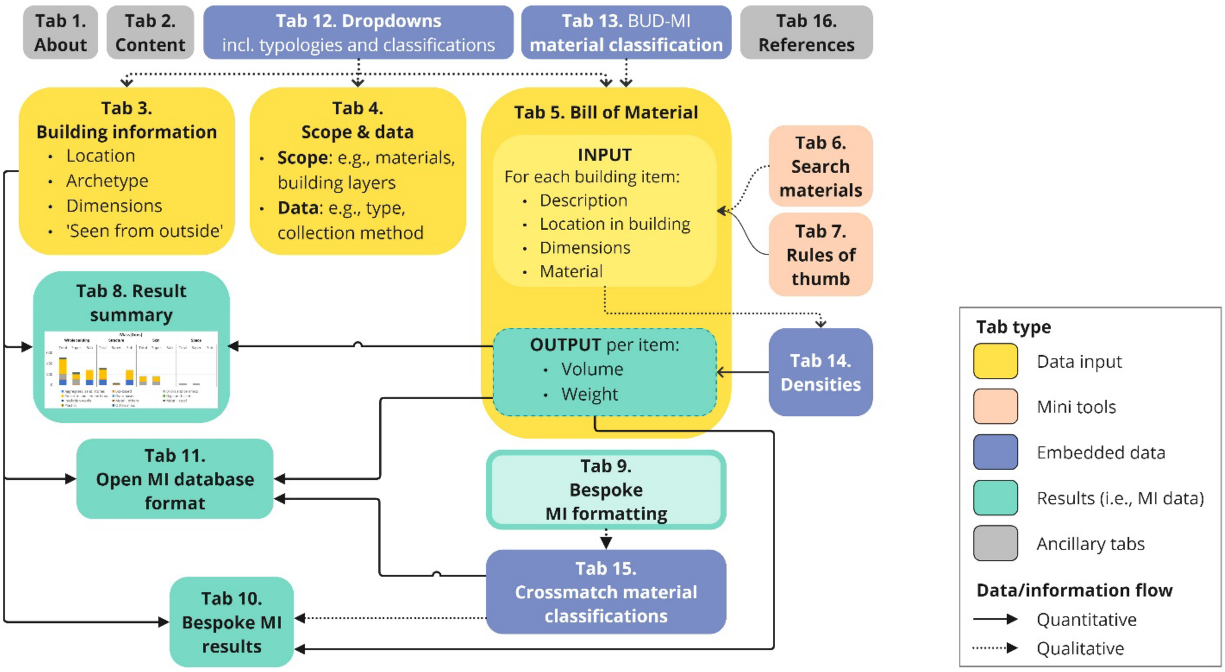

Figure 1 Architecture of BUD-MI.

3. General information and tips

3.1. Units

BUD-MI uses the metric system. As such, mass units are expressed in kilogram (kg) or metric tons (t), and lengths, areas, and volumes are expressed in meter (m), square meter (m^2), and cubic meter (m^3), respectively.

3.2. User interaction features

Several interaction features were implemented into BUD-MI. Their aim is to increase useability and intuitiveness but also to ensure a harmonized and systematic approach to data collection.

- *Protection of the spreadsheets*

The spreadsheet is protected to avoid mistakenly changing formulas. However, there is **no password**, and the protection can easily be removed. To make modification to BUD-MI, simply remove the protection by going to Review > Unprotect Sheet.

- *Color coding*

Cells follow the following color-coding throughout the template:

Yellow	**Searchable dropdown lists**<br>Search and select the most relevant option.
Grey	**Automatic calculation**<br>Overriding the formula may impact on the functionalities of BUD-MI.
Light Blue or White	**Manual entry field**<br>Manually insert relevant values or comment.

- *Pop ups and info*

Help and hint texts are available throughout BUD-MI. They are marked with the question mark symbol ⍰ and consist of definitions and/or refer to the relevant section of this user guide.

- *Dropdowns*

BUD-MI relies on searchable dropdowns so users may find options effortlessly. All dropdown lists can be found in the tab “Dropdowns”.

- *Mini tools*

Two mini tools are integrated in BUD-MI: “Rules of thumb” and “Find material”. They are described in Chapter II. Mini Tools.

3.3. Modifying & complementing BUD-MI

Although extensive efforts have been made to encompass a wide range of construction materials (along with their corresponding densities), it is likely that some materials may be missing – especially those highly location-specific, unconventional, or associated with older construction techniques. Consequently, users may need to supplement the material and density data tabs with such materials. While this task is not complex, it should be approached methodically and step-by-step. Detailed instructions for this process are provided in Chapter V Modifying & Complementing BUD-MI.

Chapter I.

DATA INPUT

This chapter contains explanations around the data that should be inputted in BUD-MI.

1. BUILDING INFORMATION

The tab “Building information” pertains to various characteristics of the building, from its use to its dimensions. A differentiation is made between “mandatory” and “optional” data to reflect two levels of data completeness: basic MI and elevated MI data. Given the analytical benefits brought by elevated MI data, *users are urged to spend a few extra minutes to fill in a maximum of information fields*. In the next pages of this User Guide, mandatory fields are signaled with the following symbol: ***Mandatory***

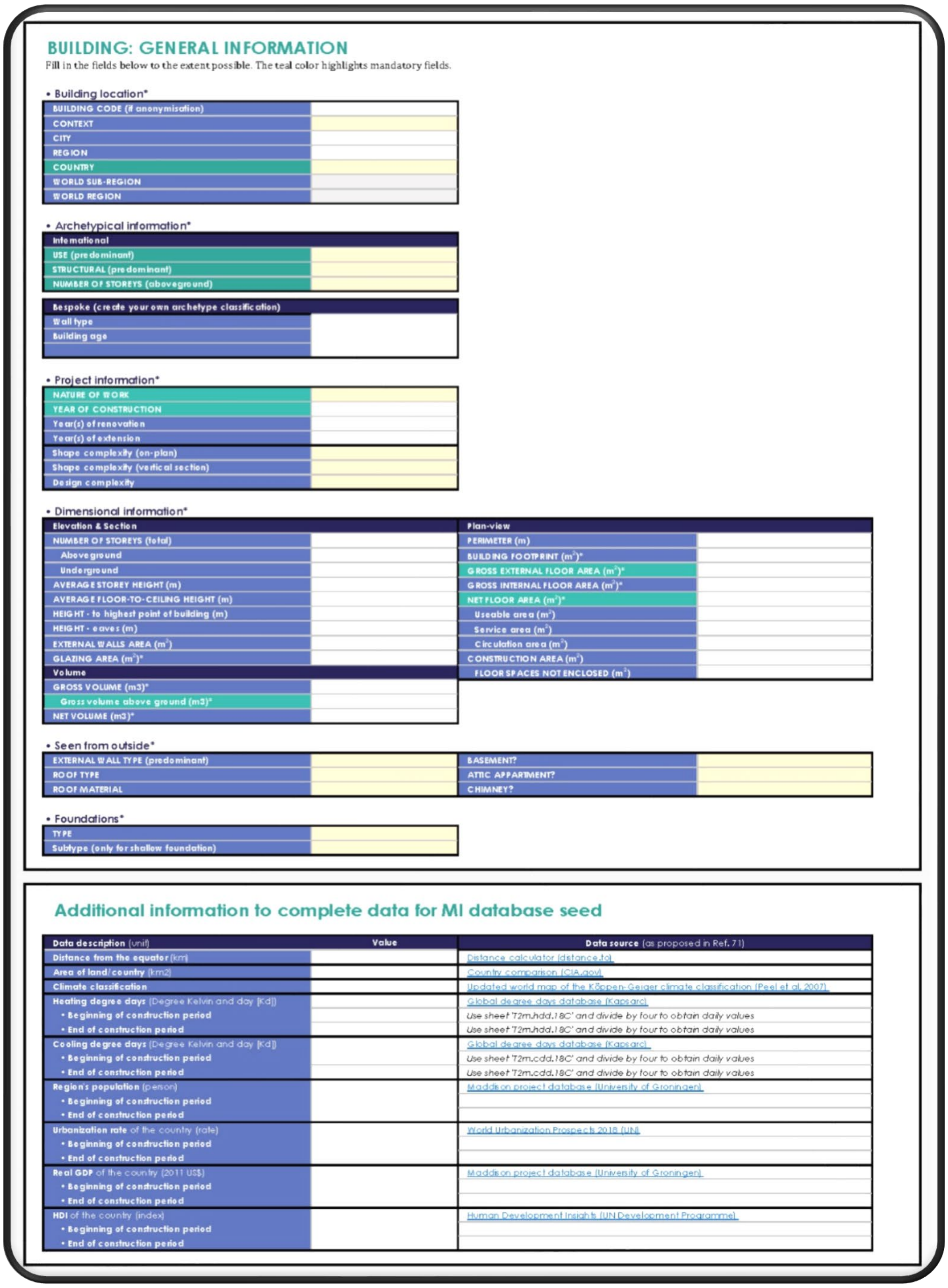

BUILDING: GENERAL INFORMATION

Fill in the fields below to the extent possible. The teal color highlights mandatory fields.

• Building location*

BUILDING CODE (if anonymisation)	
CONTEXT	
CITY	
REGION	
COUNTRY	
WORLD SUB-REGION	
WORLD REGION	

• Archetypical information*

International	
USE (predominant)	
STRUCTURAL (predominant)	
NUMBER OF STOREYS (aboveground)	

Bespoke (create your own archetype classification)	
Wall type	
Building age	

• Project information*

NATURE OF WORK	
YEAR OF CONSTRUCTION	
Year(s) of renovation	
Year(s) of extension	
Shape complexity (on-plan)	
Shape complexity (vertical section)	
Design complexity	

• Dimensional information*

Elevation & Section		Plan-view	
NUMBER OF STOREYS (total)		PERIMETER (m)	
Aboveground		BUILDING FOOTPRINT (m^2)*	
Underground		GROSS EXTERNAL FLOOR AREA (m^2)*	
AVERAGE STOREY HEIGHT (m)		GROSS INTERNAL FLOOR AREA (m^2)*	
AVERAGE FLOOR-TO-CEILING HEIGHT (m)		NET FLOOR AREA (m^2)*	
HEIGHT - to highest point of building (m)		Useable area (m^2)	
HEIGHT - eaves (m)		Service area (m^2)	
EXTERNAL WALLS AREA (m^2)		Circulation area (m^2)	
GLAZING AREA (m^2)*		CONSTRUCTION AREA (m^2)	
Volume		FLOOR SPACES NOT ENCLOSED (m^2)	
GROSS VOLUME (m3)*			
Gross volume above ground (m3)*			
NET VOLUME (m3)*			

• Seen from outside*

EXTERNAL WALL TYPE (predominant)		BASEMENT?	
ROOF TYPE		ATTIC APPARTMENT?	
ROOF MATERIAL		CHIMNEY?	

• Foundations*

TYPE	
Subtype (only for shallow foundation)	

Additional information to complete data for MI database seed

Data description (unit)	Value	Data source (as proposed in Ref. 71)
Distance from the equator (km)		Distance calculator (distance.to)
Area of land/country (km2)		Country comparison (CIA.gov)
Climate classification		Updated world map of the Köppen-Geiger climate classification (Peel et al. 2007)
Heating degree days (Degree Kelvin and day [Kd])		Global degree days database (Kapsarc)
• Beginning of construction period		Use sheet 'T2m.hdd.18C' and divide by four to obtain daily values
• End of construction period		Use sheet 'T2m.hdd.18C' and divide by four to obtain daily values
Cooling degree days (Degree Kelvin and day [Kd])		Global degree days database (Kapsarc)
• Beginning of construction period		Use sheet 'T2m.cdd.18C' and divide by four to obtain daily values
• End of construction period		Use sheet 'T2m.cdd.18C' and divide by four to obtain daily values
Region's population (person)		Maddison project database (University of Groningen)
• Beginning of construction period		
• End of construction period		
Urbanization rate of the country (rate)		World Urbanization Prospects 2018 (UN)
• Beginning of construction period		
• End of construction period		
Real GDP of the country (2011 US$)		Maddison project database (University of Groningen)
• Beginning of construction period		
• End of construction period		
HDI of the country (index)		Human Development Insights (UN Development Programme)
• Beginning of construction period		
• End of construction period		

Figure 2 Overview of the tab "**Building information**”.

1.1. Building Location

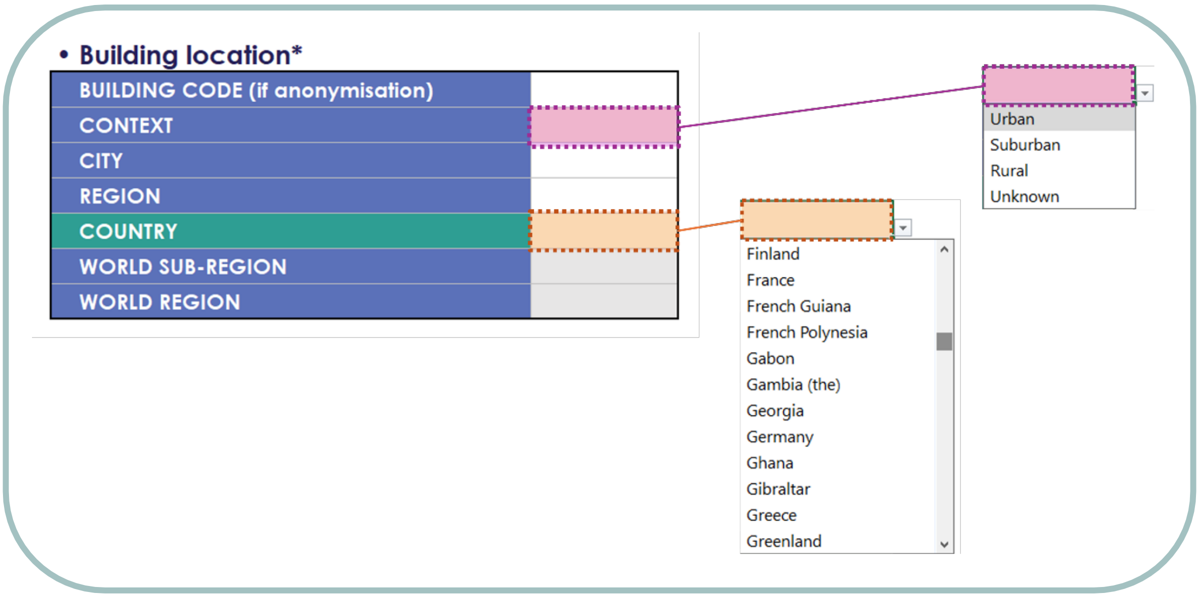

Figure 3 "**Building location**" data fields in BUD-MI.

1.1.1. Building code (if anonymization)

If the building needs anonymization, enter its anonymous code in this field, to be able to find it easily.

1.1.2. Context

Choose between urban, suburban, and rural, depending on the context in which the building is located. If unknown, select "unknown".

1.1.3. City and Region

The city and region in which the building is located can be entered manually.

1.1.4. Country ***Mandatory***, World sub-region and region

The city, region, and country in which the building is located. By selecting the country, the region and subregion fields are automatically filled.

Note that the list of countries and corresponding world sub-regions and world regions follows the classification of the United Nations Statistics Division, (United Nations Statistics Division, 1999) on which the International Organization for Standardization (ISO) aligns to define country codes (ISO 3166).

1.2. Archetypical information

Archetype categories are developed to fit the format of the inventory data for which the MI is being developed. Building archetypes are usually created by classifying a building according to its function (e.g., residential, commercial, office), its main structure (e.g., concrete frame, load bearing), its building's construction period (e.g., 1850-1900), its height (e.g. 0-3 stories, 4-7 stories), or – often – a combination of those.

In BUD-MI, an international archetype classification is embedded, with the aim of homogenizing results to improve their comparability across studies. Additionally, a "Bespoke archetype" section is intentionally left open-ended, allowing users to fill in the blank using project-specific archetype categorization.

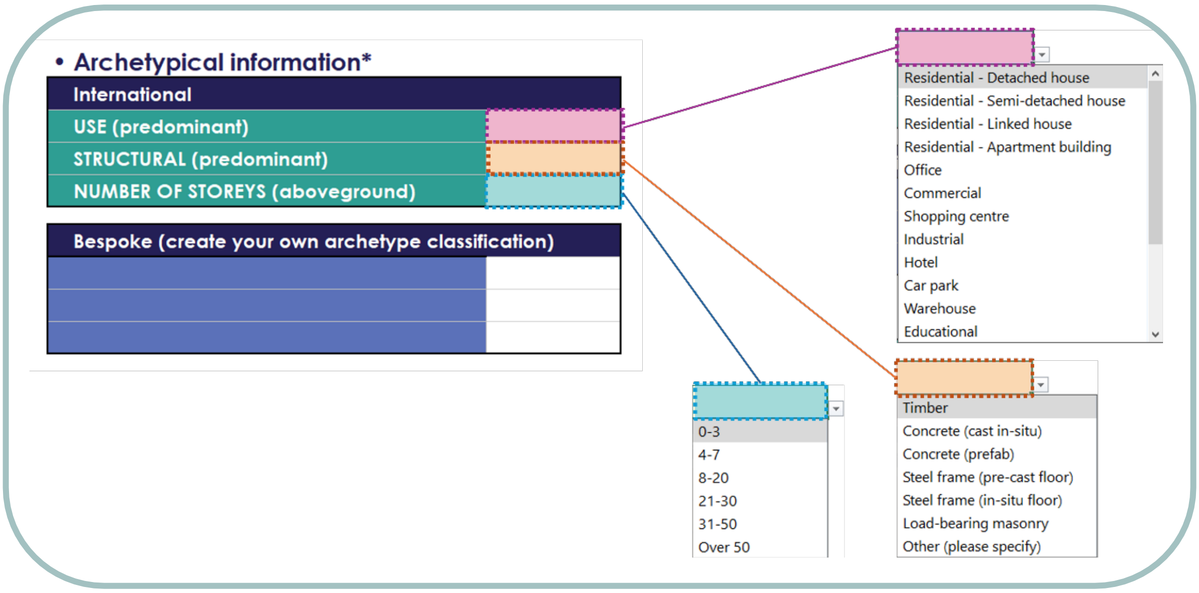

Figure 4 "**Archetypical information**" data fields in BUD-MI.

1.2.1. International archetype classification

The international archetype classification of BUD-MI consists of "building use x number of story x structural type".

- *Building use* **Mandatory**

Also called "functional type". Select the predominant building use. For example, in the case of an apartment building with a shop on the ground floor, users should select "apartment building".

The list of building use (aka functional type) is presented in Table 4. This list is based on the extensive work performed by the International Cost Management Standard Coalition (ICMS), who worked on global consistency for presenting construction lifecycle costs and carbon emissions. (ICMS Coalition, 2021) Only one addition was made to the building use categories, namely the disaggregation of residential buildings into further sub-types.

Table 4 List of building use, categorized between residential and nonresidential buildings, used in the international archetype classification in BUD-MI.

Level 1	Level 2
Residential	Detached house
	Semi-detached house
	Linked house
	Apartment building
Nonresidential	Office
	Commercial
	Shopping centre
	Industrial
	Hotel
	Car park
	Warehouse
	Educational
	Hospital
	Airport terminal
	Railway station
	Ferry terminal
	Plant facility
	Other

- *Height: number of stories aboveground* **Mandatory**

Select the story grouping that is most relevant to the building under analysis.

The groupings of story counts are based on ICMS. (ICMS Coalition, 2021) They are listed in Table 5.

Table 5 Grouping of story counts used in the international archetype classification in BUD-MI.

Grouping of story counts
0-3 stories
4-7 stories
8-20 stories
21-30 stories
31-50 stories
Over 50 stories

- *Structural type* **Mandatory**

Select the structural type most relevant to the building under analysis. If none of the option fit, users may specify the structural type manually.

The categorization of structural types is based on the ICMS and was further adapted to better integrate MI and CE considerations. (ICMS Coalition, 2021) The structural types are listed in Table 6.

Table 6 Building structural types used in the international archetype classification in BUD-MI.

Structural types
Timber
Concrete (cast in-situ)
Concrete (prefab)
Steel frame (pre-cast floor)
Steel frame (in-situ floor)
Load-bearing masonry
Other, to be specified by users

1.2.2. Bespoke archetype

These fields are intentionally left open-ended. Users are free to use any archetype classification they like; this is entirely dependent on the project for which the MI is being developed. Worth noting is that if the MIs are calculated for use in a project where a building inventory is available, the bespoke archetype classification should match the inventory data in terms of e.g., use type, construction year, and/or any other archetypical attribute available in the inventory.

1.3. Project information

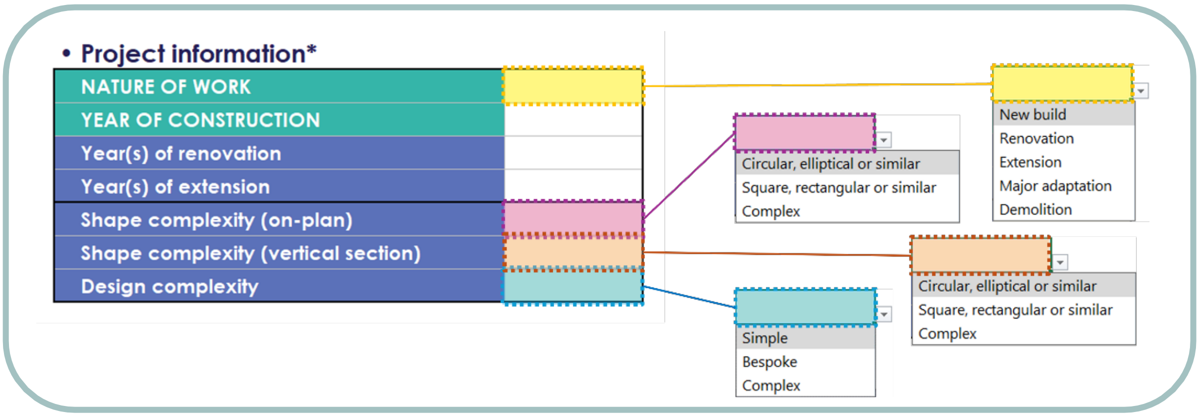

Figure 5 "**Project information**" data fields in BUD-MI.

1.3.1. Nature of work **Mandatory**

Select the nature of the work (Table 7) presented in the building documents used for MI data collection, regardless of the status in which the building currently is.

Table 7 Definition of terminology used in BUD-MI to describe the nature of the works undergone by the sampled building.

Nature of work	Definition	Reference
**New build**	The building was recently built or is in the process of being built (aka new construction).	Cambridge dictionary. (2023) New build.
**Major Adaptation**	A substantial modification / adaptation / extension of, or improvement was made to the main parts of the building.<br>Note that retrofitting, rehabilitation, and renovation fall under this term.	(ICMS Coalition, 2021)<br>(Shahi et al., 2020)
**- refurbished**	The ***original use*** of the building was kept.	(Shahi et al., 2020)
**- converted**	The use/function of the building was ***changed***.	(Shahi et al., 2020)
**Demolished**	The building was physically removed and disposed of.	(ICMS Coalition, 2021)

1.3.2. Year of construction, renovation, and extension *Mandatory*

Should the building have been renovated or extended, enter the year of such a process.

1.3.3. Shape complexity

The term complexity pertains to the relative intricacy of the shape of a building – on plan and along its vertical section. (ICMS Coalition, 2021)

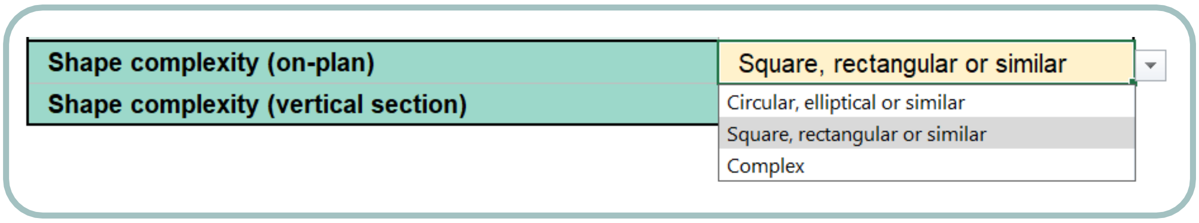

Figure 6 Available dropdown list to describe the shape complexity of a building in BUD-MI. Adapted from ICMS Coalition (ICMS Coalition, 2021).

1.4. Dimensional information

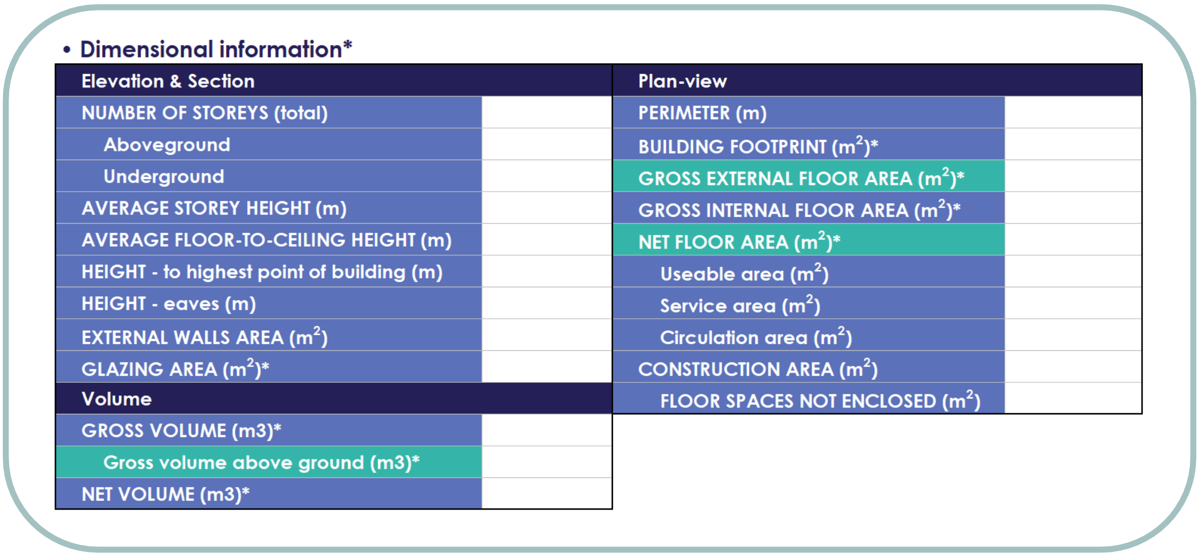

Figure 7 "**Dimensional information**" data fields in BUD-MI.

Many dimensional information can be registered from a building plan. In the context of transferability across countries and across building inventories, it is of outmost importance that

as many of these quantities are recorded when documenting the sample building. (Schiller et al., 2019) Dimensional information is divided into three sections: vertical dimensions that are relevant to the building's elevation and section, plan-view dimensions, and volume dimensions.

The three highlighted fields are mandatory: gross external floor area, net floor area, and gross volume above ground. Recording them is good practice that supports transferability and comparability of MIs.

Note that areas should be recorded at a maximum of two decimal places. (ISO 9836:2017, 2017)

1.4.1. Elevation & Section

See the definitions of different volumes in Table 8 below.

Table 8 Definitions of dimensions related to elevations and cross-sections used in BUD-MI.

Dimensions	Unit	Definition	Reference
Number of stories (total)	-	Total number of stories in the building.	-
Aboveground	-	Number of stories aboveground.	-
Underground		Number of stories underground.	-
Average story height	m	Average height between the floors' surfaces of two consecutive stories.	-
Average floor-to-ceiling height	m	Average height between the floor's surface and the ceiling's underside.	Adapted from ISO 9836:2017
Height – to highest point of building	m	Height between the finished -ground and the highest point of the roof.	-
Height – eaves	m	Height between the finished ground and the eaves of the roof.	-
External walls area	m^2	Area of external wall above the finished ground. If parts of the foundations appear aboveground, include those in the calculation.	Adapted from ISO 9836:2017
Glazing area	m^2	Area of transparent material. Frames (e.g., window frame) are not included.	(UK Government, 2021)

1.4.2. Plan view

See the definitions of different plan-view dimensions in Table 9 below.

- *Perimeter*

Building perimeters are used by quantity surveyors to find out the total lengths of external walls and their finishes but also strip foundations. (Cunningham, 2015) In the context of MI and MS calculation, the perimeter measurement of the building can be used to generate the elemental MI of external walls and strip foundations. These MIs should then be stated in "kilograms per running meter of perimeter". Such MIs can, in turn, be used with GIS-polygon perimeters to approximate the MS of external walls in a geospatial building inventory.

It should be noted that GIS polygons. often generated from satellite or aerial images, often include covered spaces – but not enclosed ones (e.g., terraces). Additionally, depending on the inventory quality, GIS polygons may encompass attachments like annexes, garages, and sheds. Thus, using running-meter elemental MI introduces some layer of uncertainty.

- *Areas*

BUD-MI uses the list of dimensional information of ISO 9836:2017, in line with (Schiller et al., 2019) and (Heeren & Fishman, 2019). All areas are to be measured in square meters (m^2).

Table 9 Definitions of types of area related to plan view used in BUD-MI. References: (BREEAM, 2016; IPMSC, 2023; ISO 9836:2017, 2017)

Plan-view	Unit	Definition	Reference
Perimeter	m	· External perimeter of the building.	-
Building footprint	m^2	· Also called 'Covered Area'.<br>· Calculation: area of vertical projection of external dimensions of the building onto the ground.<br>· Exclude secondary components (e.g., staircases, roof overhangs) and outdoor facilities (e.g., greenhouse, outhouse).	(ISO 9836:2017, 2017), (IPMSC, 2023)
Gross external floor area<br>**Mandatory**	m^2	· Acronym: GEFA<br>· Also called 'Total Floor Area'.<br>· It is the area of all floor space covered and enclosed to full height, for all levels (incl. attics, basements, etc.).<br>· Calculation: measured to the outside face of outside walls.<br>· Exclude floor area not fully enclosed.<br>· Equivalent of "Floor Area" in IPMSC.	(ISO 9836:2017, 2017), (IPMSC, 2023)
Gross internal floor area	m^2	· Acronym: GIFA<br>· Also called 'Intra-muros area'.<br>· It is the Gross External Floor Area less the floor area taken up by the external walls.<br>· Calculation: Gross External Floor Area - Floor Area of External Walls<br>· Equivalent of [Floor Area] minus [Component Areas A1+A2] in IPMSC.	(ISO 9836:2017, 2017), (IPMSC, 2023)
Net floor area<br>**Mandatory**	m^2	· Acronym: NFA<br>· Area between enclosing elements<br>· Calculation: Gross Floor Area - Construction Area = Gross Floor Area - [Floor Areas of external walls + internal walls + columns + partitions]<br>· Divided into Usable Area, Services Area, and Circulation Area.<br>· Equivalent of [Floor Area] minus [Component Area A] in IPMSC.	(ISO 9836:2017, 2017), (IPMSC, 2023)
· *Useable area*	m^2	· Acronym: UA<br>· Part of NFA corresponding to the purpose and use of the building.<br>· Classified according to the purpose of the building and the use to which they are put.<br>· Equivalent of Component Area F (aka "Primary Area) in IPMSC.	(ISO 9836:2017, 2017), (IPMSC, 2023)
· *Service area*	m^2	· Part of NFA with technical installations that service (parts of) the building, e.g., installation, pipe, shaft, and duct for (waste)water, heating, cooling, gas, ventilation, AC, electricity supply. Also lift, conveyor, service escalators-<br>· Equivalent of "Component Areas of B2+C" in IPMSC.	(ISO 9836:2017, 2017), (IPMSC, 2023)
· *Circulation area*	m^2	· Part of NFA used for circulation within the building.<br>· For example, area of stairwells, corridors, internal ramps, waiting areas, escape balconies, lift shafts, escalators and the like.<br>· Equivalent of "Component Areas E+B1" in IPMSC.	(ISO 9836:2017, 2017), (IPMSC, 2023)
Construction area	m^2	· Also called "Floor Area of Structural Elements".<br>· Calculation: floor areas of external walls + internal walls + columns + partition walls<br>· Equivalent of "Component Area A" in IPMSC.	(ISO 9836:2017, 2017),

			(IPMSC, 2023)
Floor spaces not enclosed	m^2	Areas that are not enclosed, such as open floors, covered ways and balconies.	(BREEAM, 2016)

1.4.3. Volume

See the definitions of different volumes in Table 10 below.

Table 10 Definitions of types of volumes used in BUD-MI.

Volume (m^3)	Definition	Reference
Gross volume	Volume of building, including roof volume and basement volume. Not included: foundations, layers of hardcore, and the like.	ISO 9836:2017
Gross volume above ground *Mandatory*	Volume of building aboveground, including roof volume. Not included: basement, foundations, layers of hardcore, and the like.	Adapted from ISO 9836:2017
Net volume	Obtained from the inner limiting faces. Net volume is calculated as the product of the net floor area by the height between the floor's surface and the ceiling's underside. Not included: volume of the roof.	ISO 9836:2017

1.5. 'Seen from outside'

With the rising amount of research on characterizing MS with the help of computer vision, satellite data, aerial imaging, hyperspectral data, and LiDAR data, recording information on building as if "seen from outside" is particularly relevant. (Lanau et al., 2024) Such information may be used to create MIs that inventories compiled through with such techniques. Therefore, users are welcome to record the type and material of walls and roof – the two key entities captured through remote sensing.

Noteworthy is that the term "seen from outside" used in BUD-MI was derived from terminology used in computer vision, namely "seen from the street-level" and "seen from above". (Ibrahim et al., 2020)

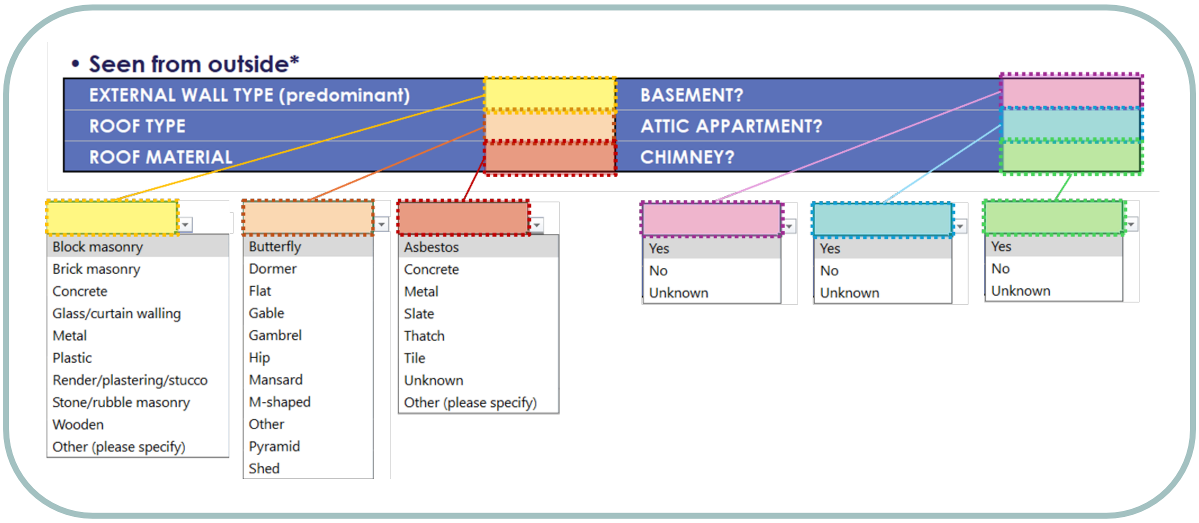

Figure 8 "**Seen from outside**" data fields in BUD-MI.

1.5.1. External wall type (dominant)

Select the type of external wall of the sample building.

In the context of computer vision and seen-from-outside, 'external wall type' refers to the primary material of the external walls visible from the outside. The various external wall types listed in BUD-MI are provided in Table 11, with examples illustrated in Figure 9-Figure 17 below.

Table 11 External wall types used in BUD-MI to describe the material of the external wall as seen from outside.

External wall type (dominant)
Block masonry
Brick masonry
Concrete
Fibercement
Glass/curtain walling
Metal
Plastic
Rendering/plastering/stucco
Stone/rubble masonry
Wooden
Other (please specify)

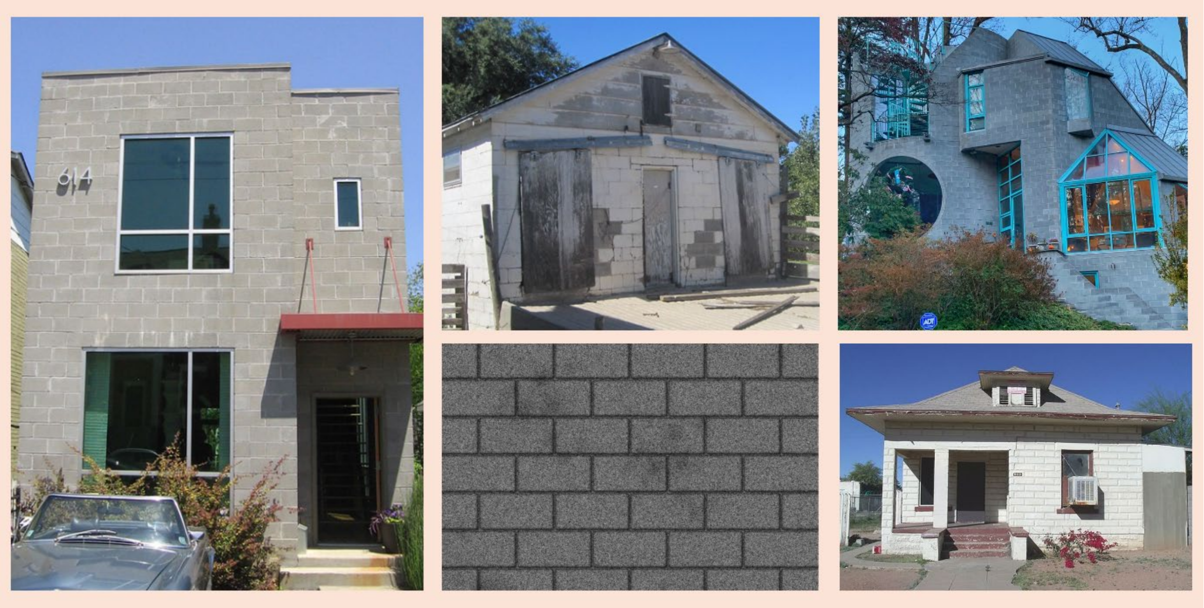

Figure 9 Examples of external walls with **block masonry façade**. All images are public domain.

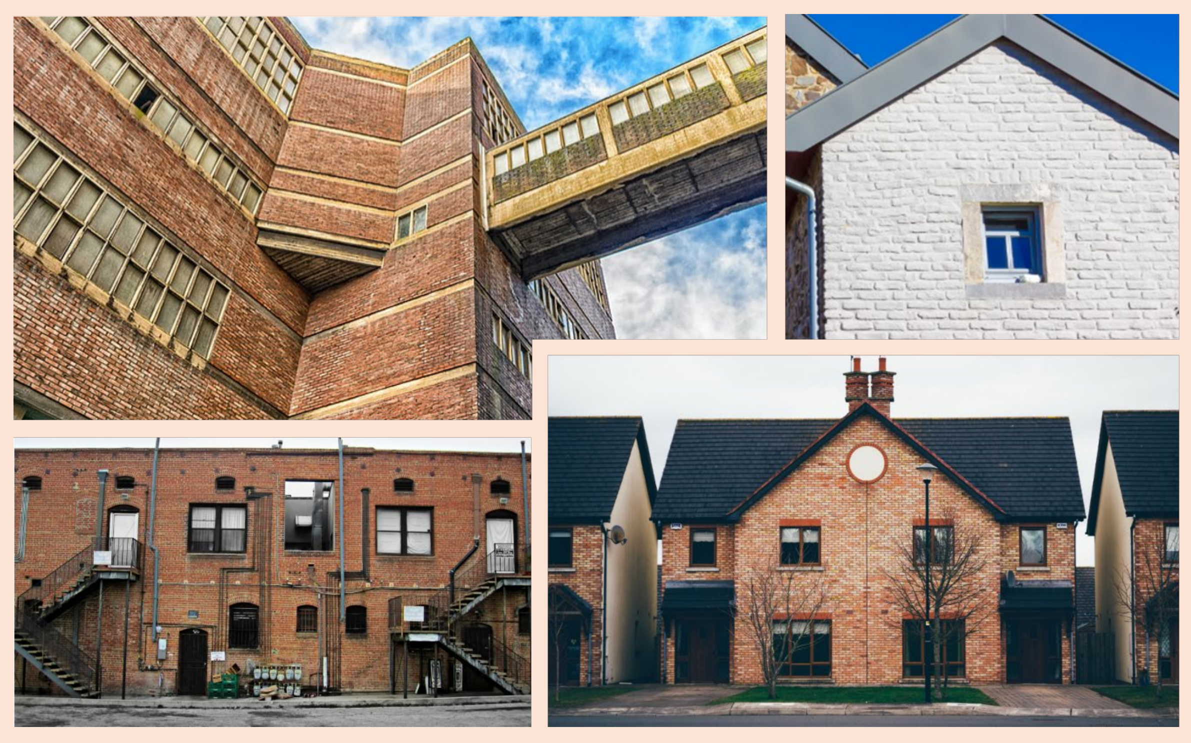

Figure 10 Examples of external walls with **brick masonry** façade. All images are public domain.

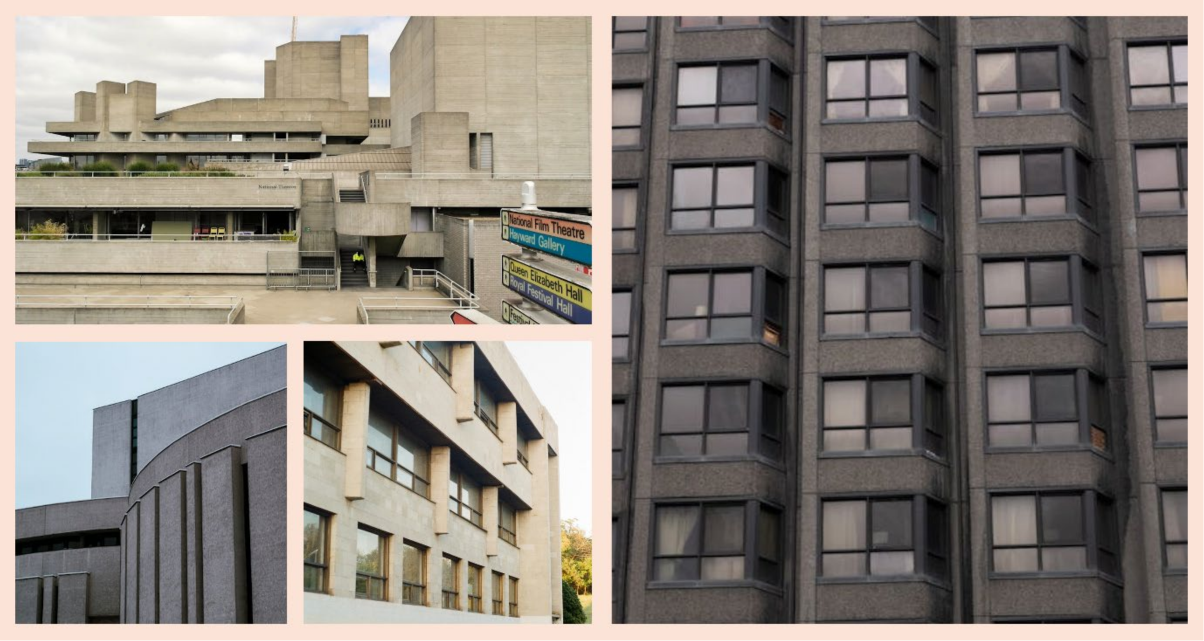

Figure 11 Examples of external walls with **concrete** façade. All images are public domain.

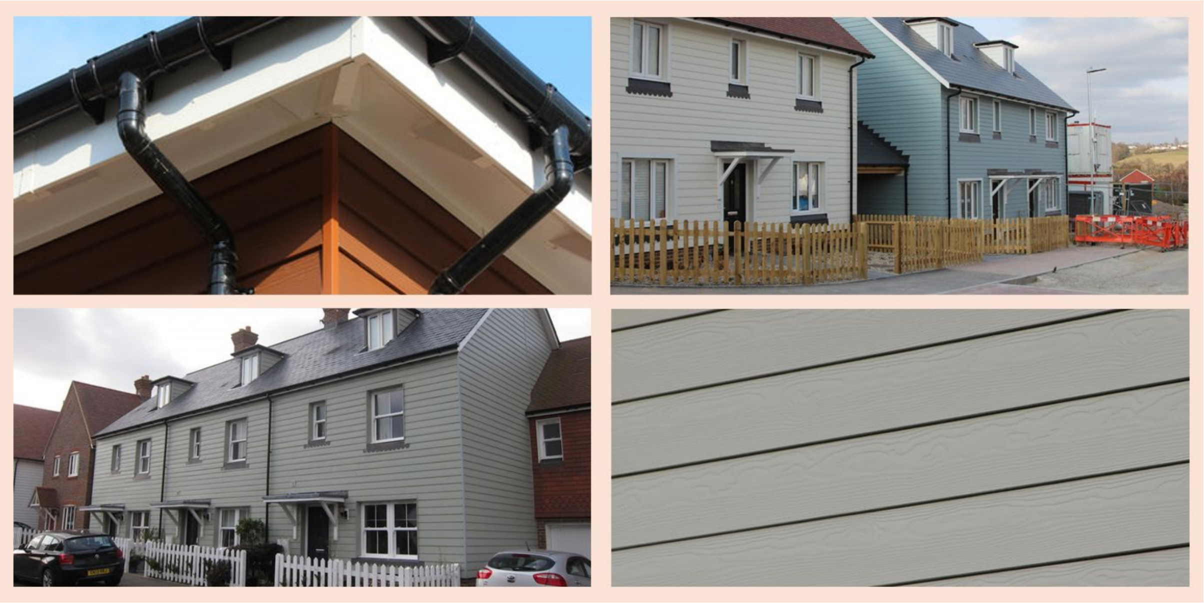

Figure 12 Examples of external walls with **fiber concrete** weatherboarding. All images are public domain.

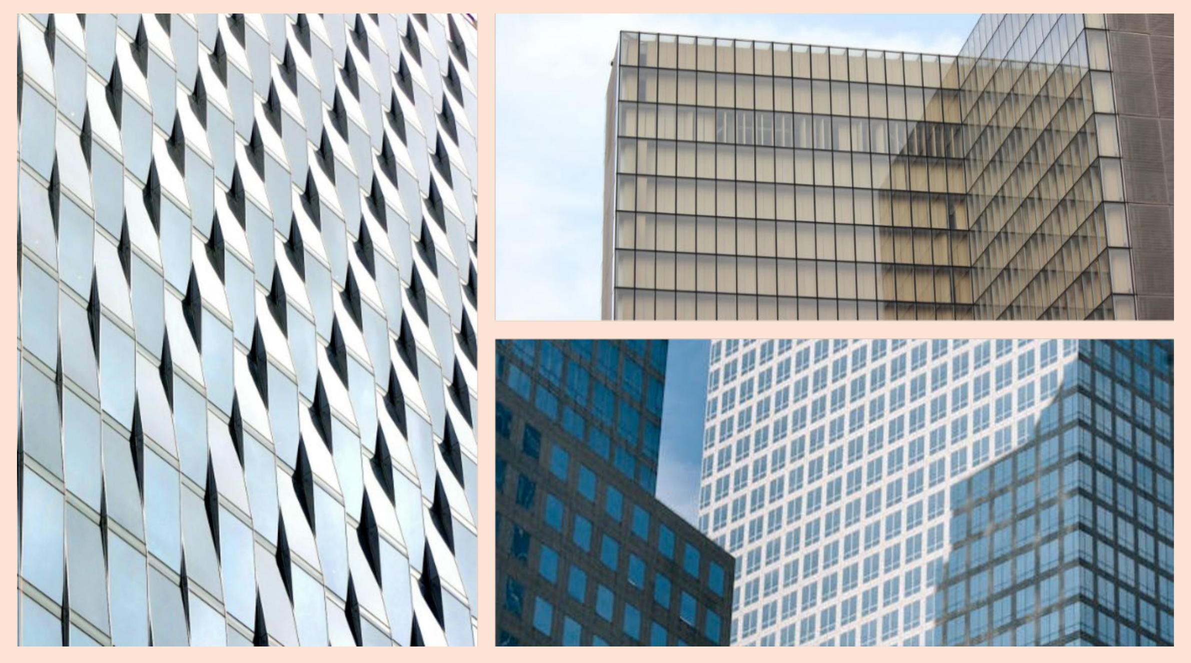

Figure 13 Examples of external walls with **glass/curtain walling** façade.
All images are public domain.

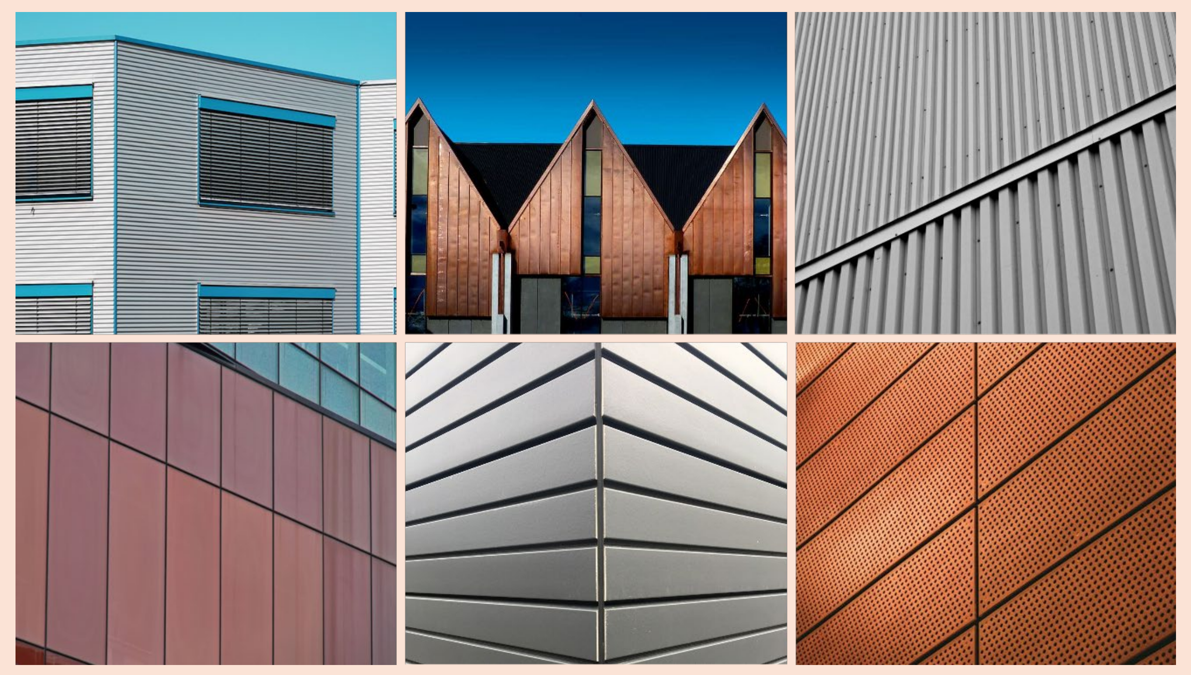

Figure 14 Examples of external walls with **metal** façade. All images are public domain.

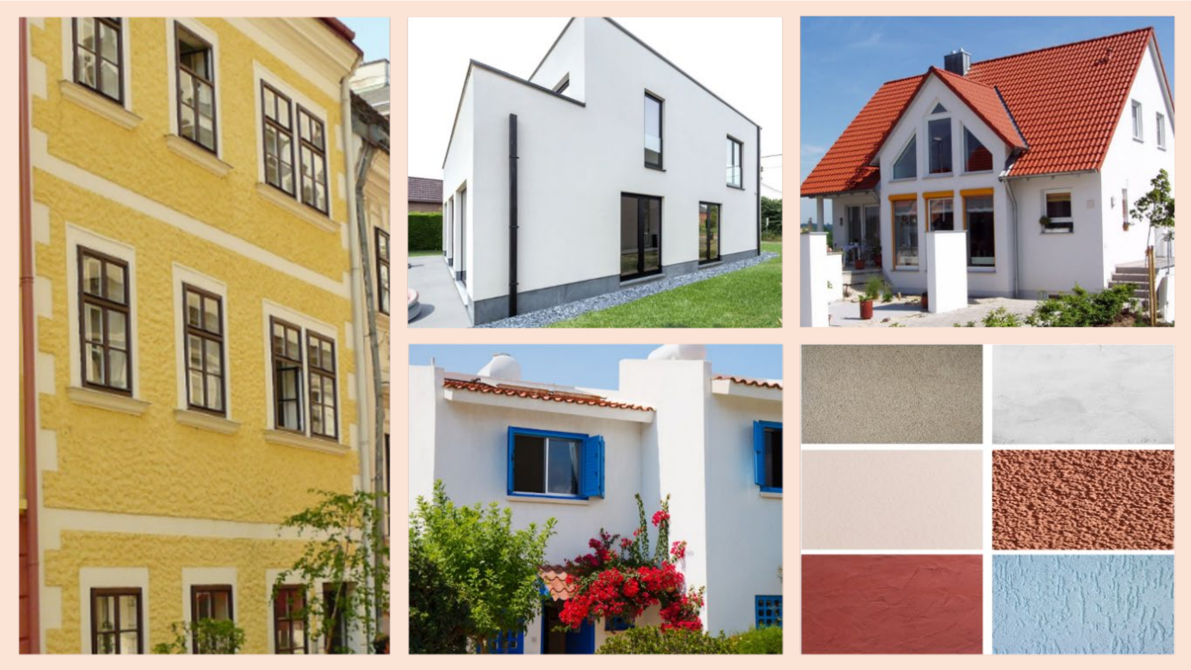

Figure 15 Examples of external walls with **rendering/stucco/plastering** façade.
All images are public domain.

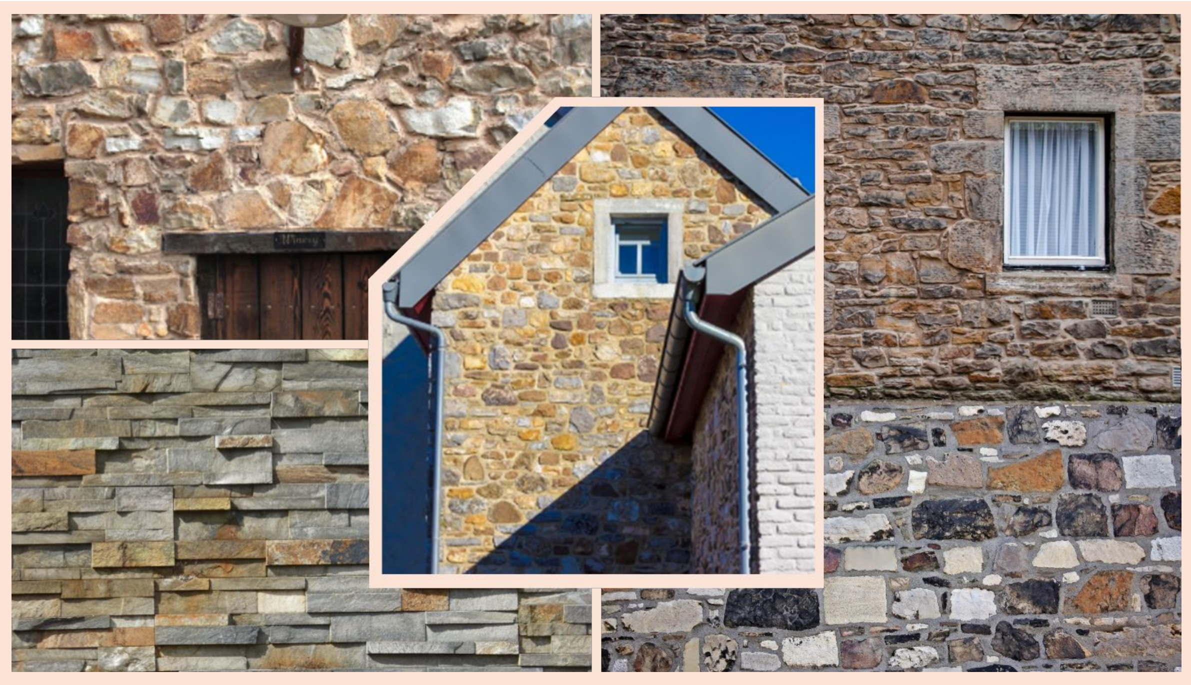

Figure 16 Examples of external walls with **stone/rubble masonry** façade.
All images are public domain.

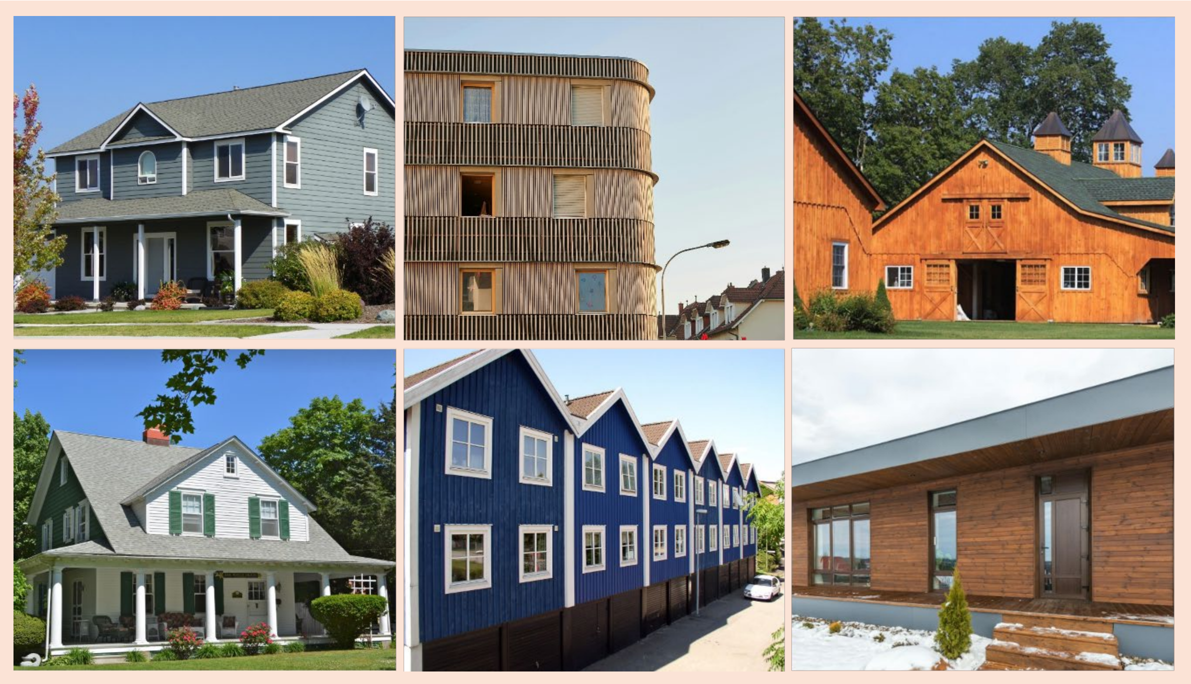

Figure 17 Examples of external walls with **wooden** façade. All images are public domain.

1.5.2. Roof type

Select the roof type of the sample building. The most common roof types are illustrated in Figure 18. Should the roof type of the sample building not fit any of the choices, the user may override the dropdown and enter the roof type manually.

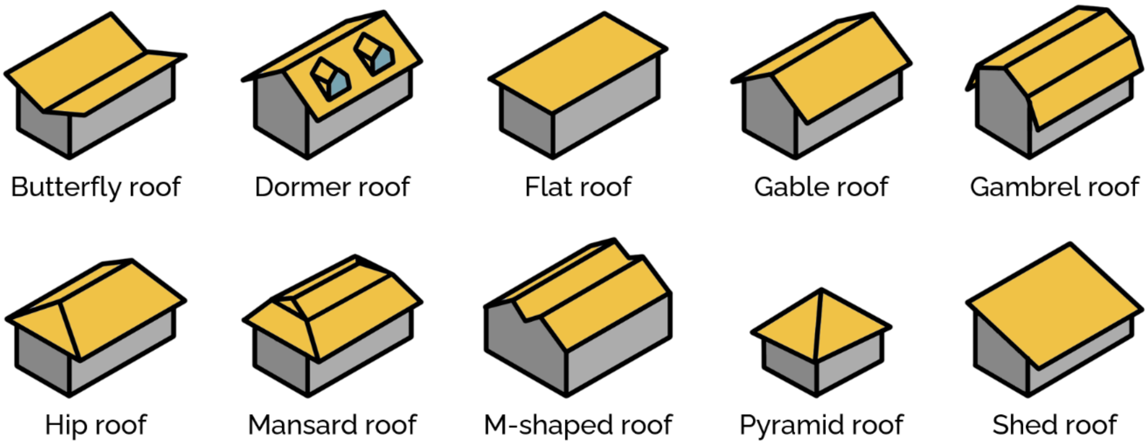

Figure 18 List and depiction of **roof types** used in BUD-MI.

1.5.3. Roof material

Select the roof material.

The various roof materials listed in BUD-MI are provided in Table 12, with examples of each in Figure 19-Figure 28 below.

Table 12 List of roof materials used in BUD-MI.

Roof material
Asbestos cement (aka AC sheet)
Asphalt shingles
Built-up roofing (aka tar-and-gravel)
Clay tiles
Concrete tiles
Fibercement sheets (aka fibro)
Membrane roofing
Metal
Slate
Thatch
Unknown
Other (please specify)

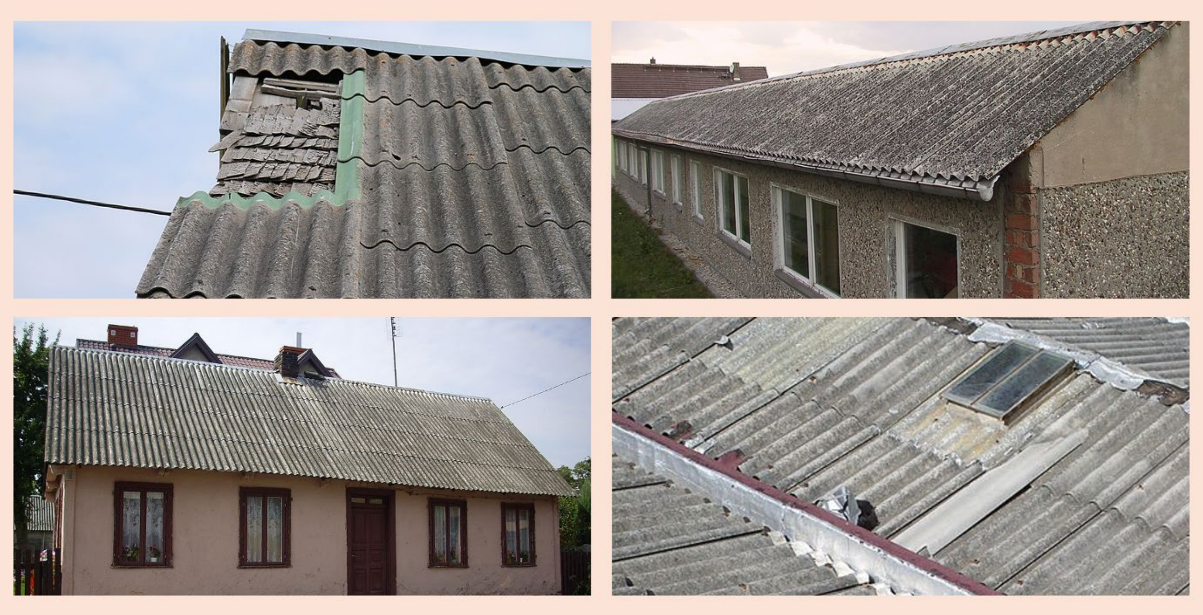

Figure 19 Example of roofs made of **asbestos cement** roofing (corrugated). Images are public domain.

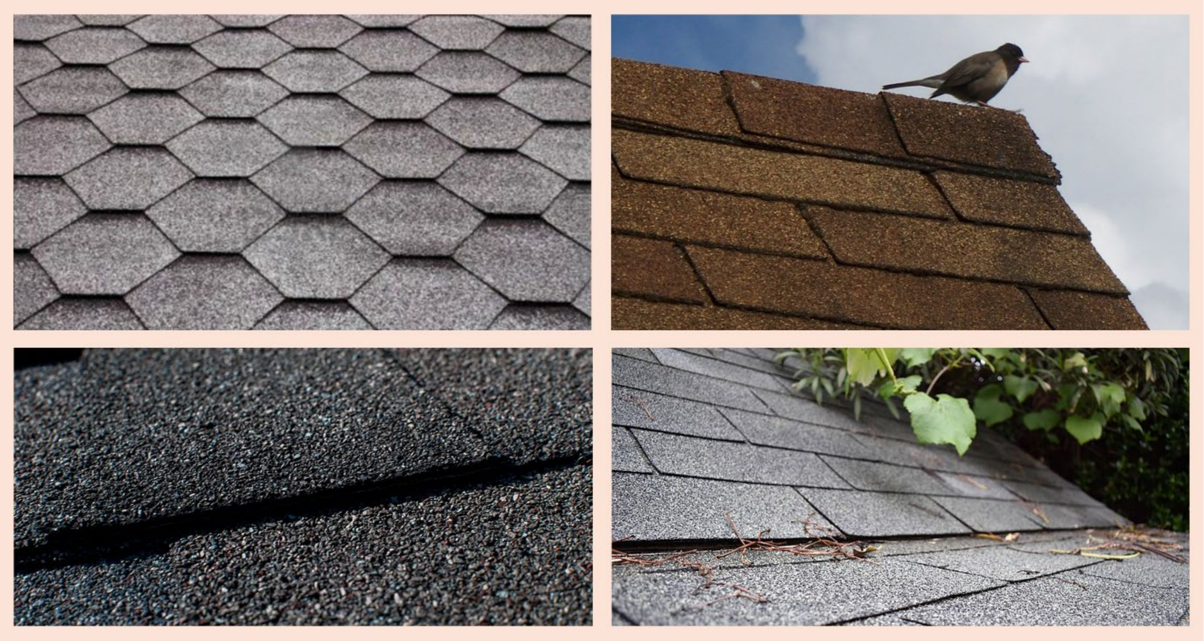

Figure 20 Example of roofs made of **asphalt shingles**..[1]

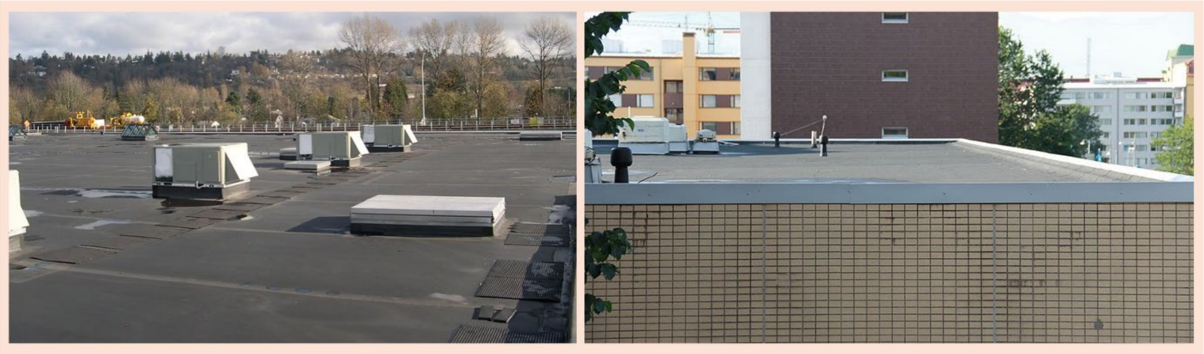

Figure 21 Examples of **built-up roofing**..[2]

[1] Upper left image: "asbestos-roof" by Asbestos Testing on Flickr. Licensed under CC BY-SA 2.0. // Upper right image: Licensed under CC BY-SA 3.0 "Dark-eyed Junco perched on an asphalt shingle roof, Seattle area, USA" by TriviaKing. // Bottom left image: "Close-up view of asphalt shingles on a roof" by Shadowmeld Photography. Licensed under CC BY-SA 4.0. // Bottom right image: "Shingle Roof" by Fastily. Licensed under CC BY-SA 4.0.

[2]Left image: "Rooftop HVAC" by Pacific Northwest National Laboratory is licensed under CC BY-NC-SA 2.0. // Right image: Flat roof, Martinkatu, Turku, Finland by Htm is licensed under CC BY-SA 3.0.

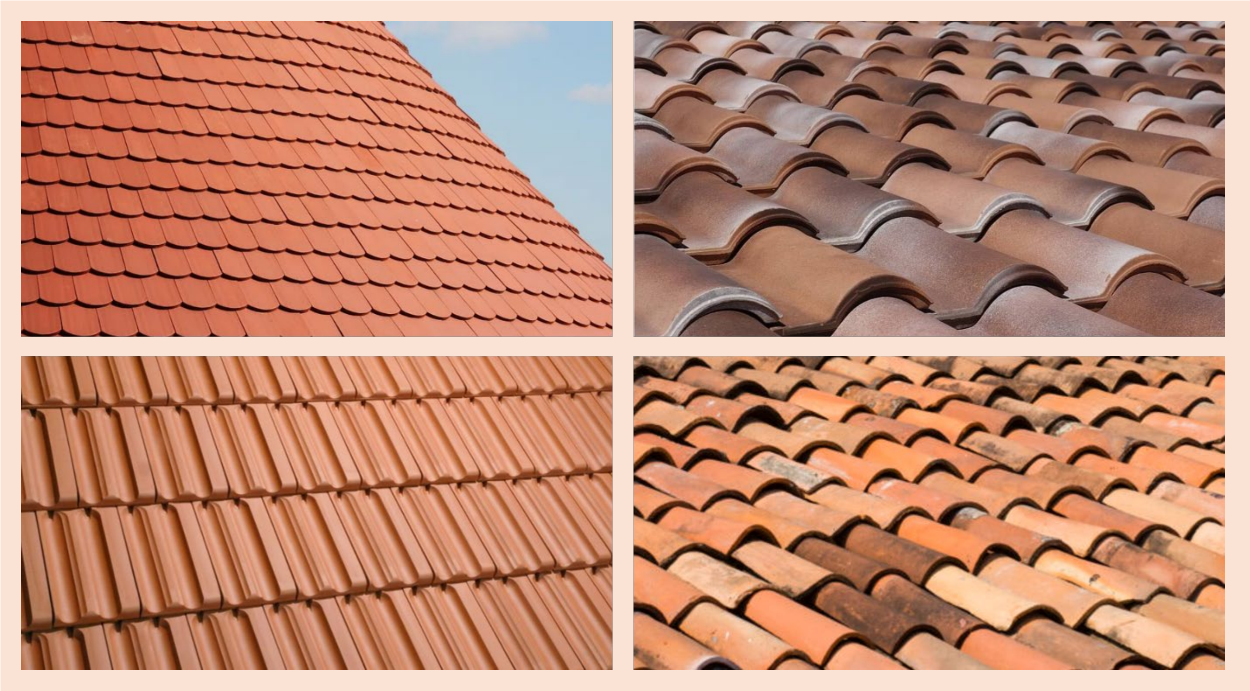

Figure 22 Examples of roof made of **clay tiles**.[3]

[3]Upper left image: "topp kakel house taket vinden" by vargazs. Free to use under Pixabay's content license. The three other images are public domain.

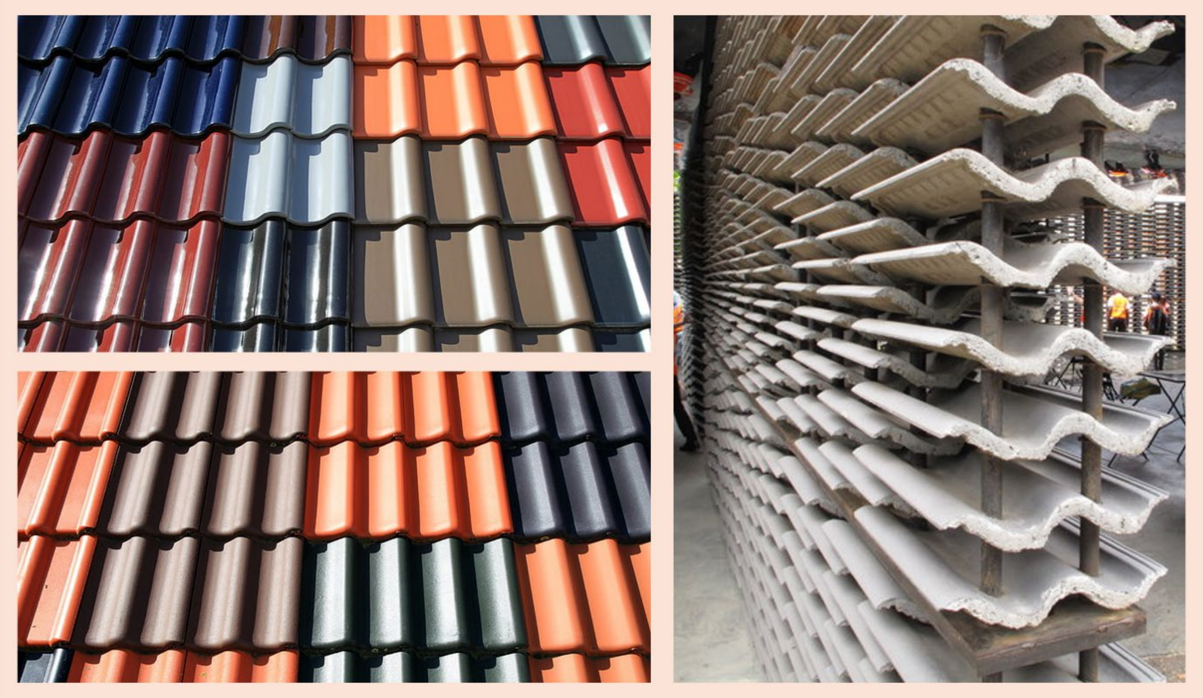

Figure 23 Example of roofs made of **concrete tiles**. Images are public domain.

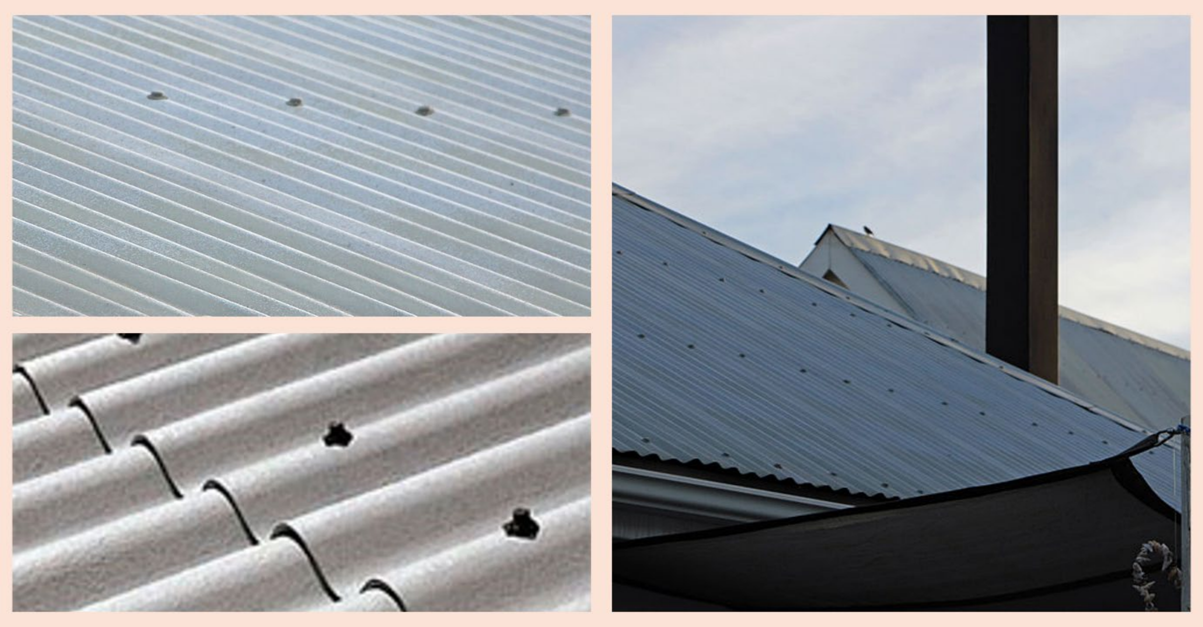

Figure 24 Example of roofs made of **fiber cement sheets (aka fibro)**. Images are public domain.

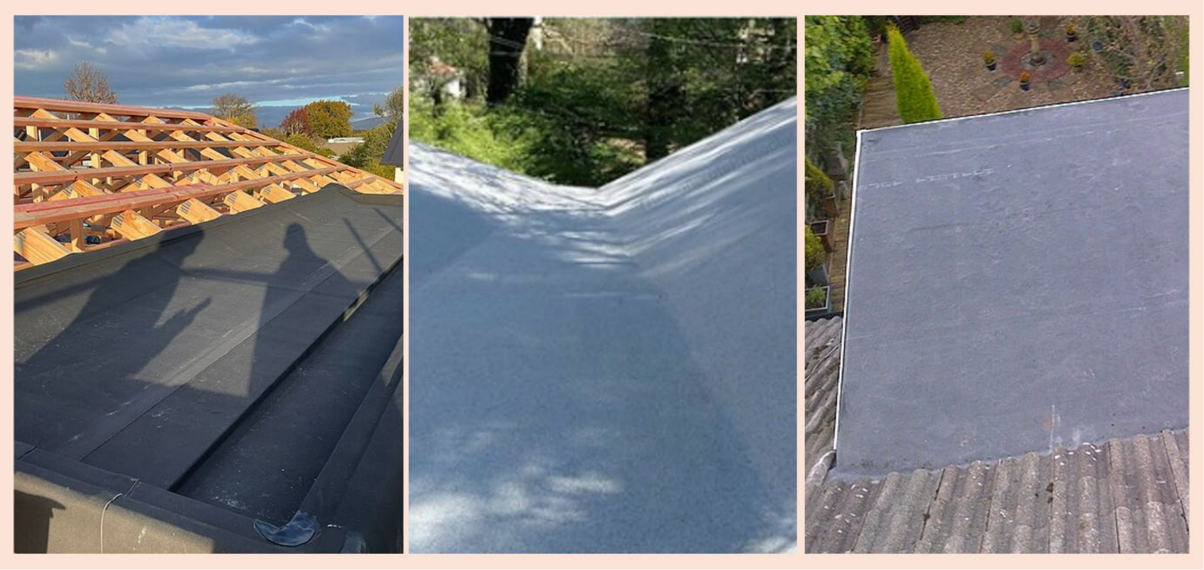

Figure 25 Example of **membrane** roofing. Images are public domain.

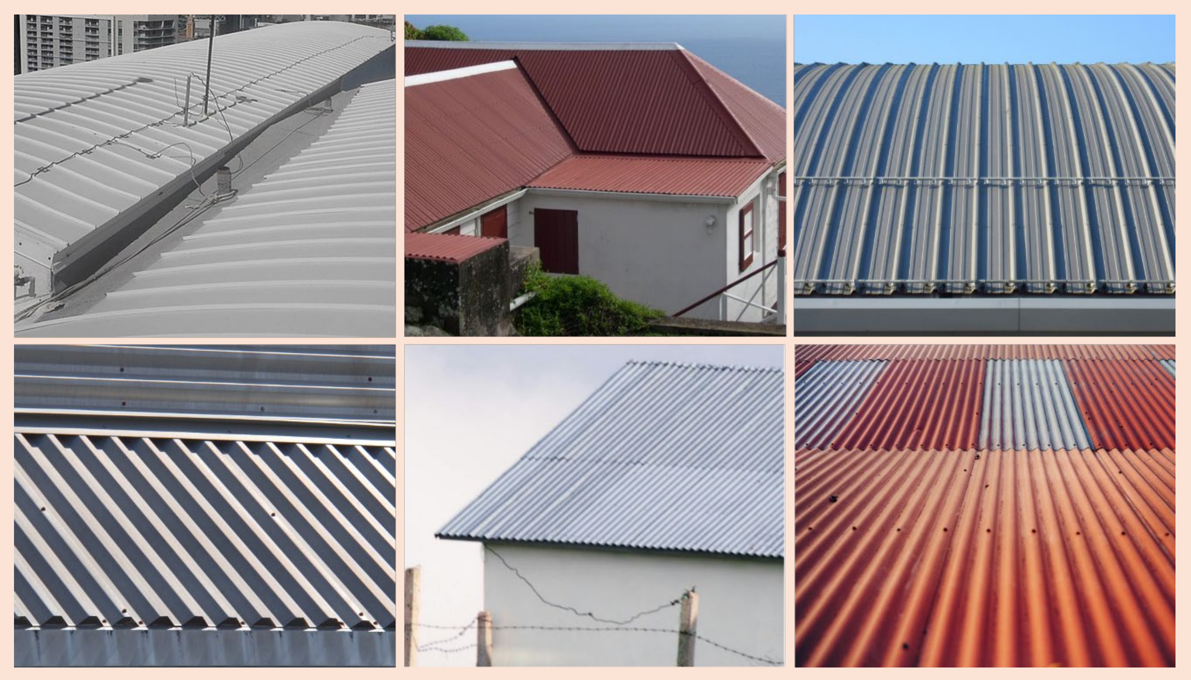

Figure 26 Example of roofs made of **metal** (here, all corrugated). Images are public domain.

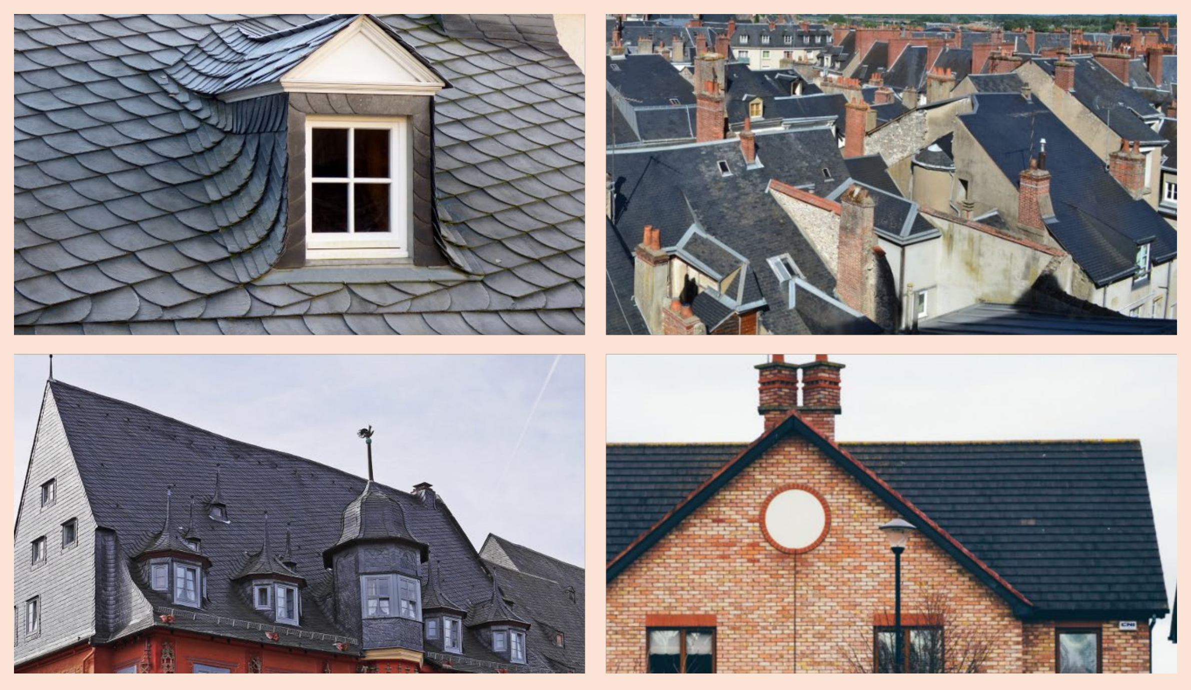

Figure 27 Example of roofs made of **slate**. Images are public domain.

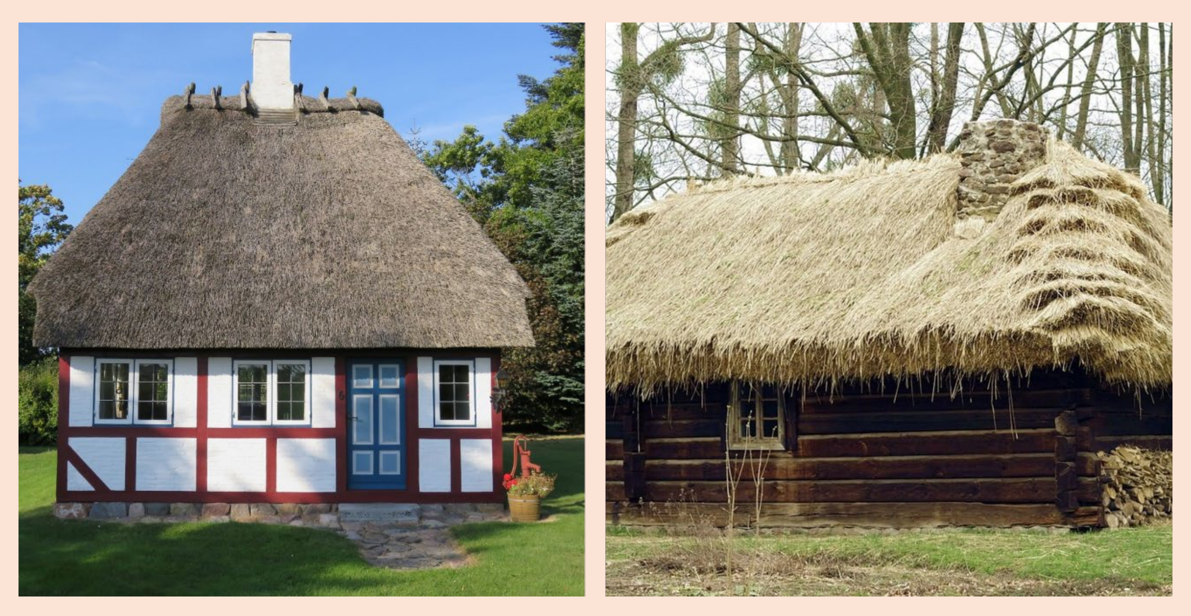

Figure 28 Example of roofs made of **thatch**. Images are public domain.

1.5.4. Basement, attic apartment, and chimney

Select the appropriate option, depending on if the building has a basement, an attic apartment (aka loft apartment), and a chimney. All such information can help with computer vision work.

If the information is lacking, select “unknown”.

1.6. Building foundations

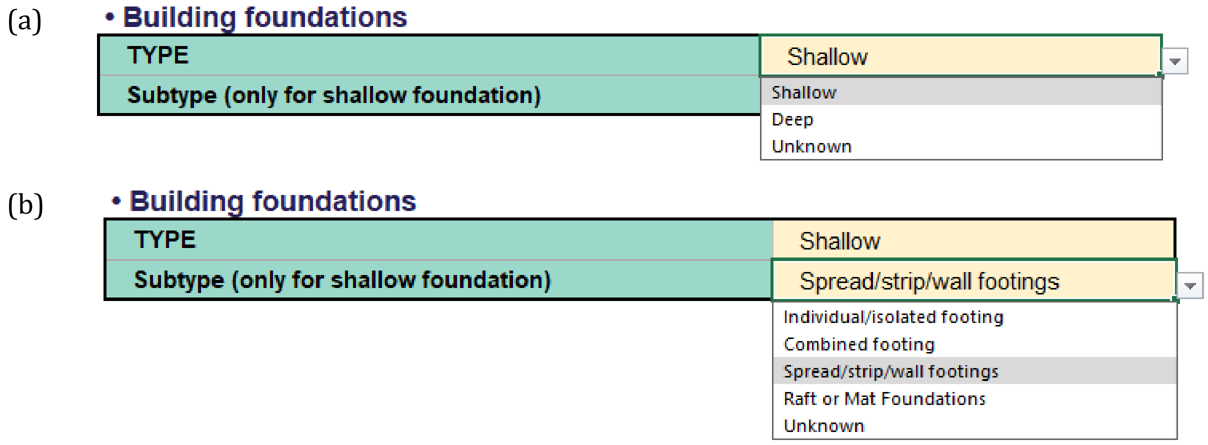

Figure 29 “**Foundations**” data fields in BUD-MI. (a) Select the most relevant **type** of foundations in the dropdown list. (b) If ‘shallow foundation’ is selected, select the most relevant **subtype** in the dropdown list.

1.6.1. Type of foundation

Specify whether the building’s foundations are shallow or deep.

- **Shallow foundations** are constructed close to the ground surface; they are used when the surface soil has the capacity to support the structure. See also Section 1.6.2 below.
- **Deep foundations** extend deep into the ground and are used when the surface soil cannot support the structure, or if the structure requires additional support due to heavy loads or environmental conditions like earthquakes or high winds. For buildings, deep foundations are also called “pile foundations”.

1.6.2. Subtype (only for shallow foundations)

If “shallow foundations” is selected, users should specify further the type of shallow foundations of the building. The types of shallow foundations that can be selected in BUD-MI are depicted below in Figure 30.

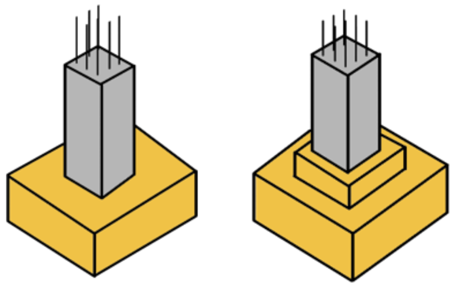

Individual/isolated footing (or pad foundation): Shaped as square or rectangle and constructed for a single column. Used when loads from the building structure is carried by columns.

Combined footing:
Used when two or more columns are close enough, resulting in their isolated footings overlapping each other. Rectangular shape. Used when loads from the building structure is carried by columns.

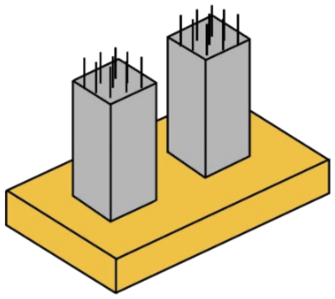

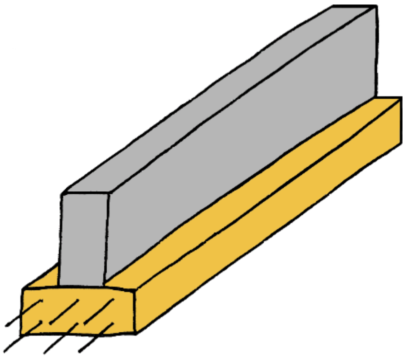

Spread/strip/continuous/wall footings:
The wider base of this footing type spreads the weight from the building structure over more area and provides better stability. used for individual columns, walls, and bridge piers where the bearing soil layer is within 3m (10 feet) of the ground surface. Soil bearing capacity must be sufficient to support the weight of the structure over the base area of the structure. It should not be used on soils where there is any possibility of a ground flow of water above bearing layer of soil; this may result in liquefaction.

Raft/mat foundations:
Continuous slab on soil extending over the footprint of the building. This type of foundation supports the heavy structural loads from walls and closely placed columns. They are suitable for weaker soils whose bearing capacity is not enough for spread and wall footings. They can also be used to avoid the shear failure of the structure due to uneven settlement.

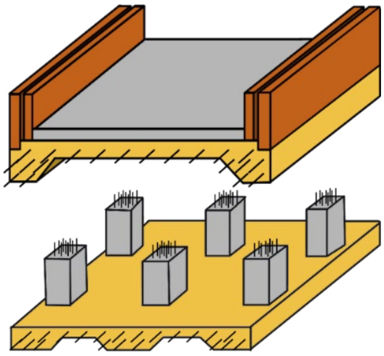

Figure 30 List and depiction of types of shallow foundations used in BUD-MI.

1.7. Database seed information

For data completion in MI database

**Data description** (unit)	**Value**	**Data source** (as proposed in Ref. 71)
**Distance from the equator** (km)		Distance calculator (distance.to)
**Area of land/country** (km2)		Country comparison (CIA.gov)
**Climate classification**		Köppen-Geiger climate classification, updated world map (Peel et al 2007)
**Heating degree days** (Degree Kelvin and day [Kd])		Global degree days database (Kapsarc)
• **Beginning of construction period**		*Use sheet 'T2m.hdd.18C' and divide by four to obtain daily values*
• **End of construction period**		*Use sheet 'T2m.hdd.18C' and divide by four to obtain daily values*
**Cooling degree days** (Degree Kelvin and day [Kd])		Global degree days database (Kapsarc)
• **Beginning of construction period**		*Use sheet 'T2m.cdd.18C' and divide by four to obtain daily values*
• **End of construction period**		*Use sheet 'T2m.cdd.18C' and divide by four to obtain daily values*
**Region's population** (person)		Maddison project database (University of Groningen)
• **Beginning of construction period**		
• **End of construction period**		
**Urbanization rate** of the country (rate)		World Urbanization Prospects 2018 (UN)
• **Beginning of construction period**		
• **End of construction period**		
**Real GDP** of the country (2011 US$)		Maddison project database (University of Groningen)
• **Beginning of construction period**		
• **End of construction period**		
**HDI** of the country (index)		Human Development Insights (UN Development Programme)
• **Beginning of construction period**		
• **End of construction period**		

Figure 31 Data fields for "**Data completion in MI database**" in BUD-MI.

In the MI database started by (Heeren & Fishman, 2019), MI data should ideally be provided together with contextual information on the building. To reach such a level of data completion, fill in all the fields in this section. For convenience, the links proposed (Heeren & Fishman, 2019) were hyperlinked into BUD-MI.

2. SCOPE OF STUDY & DATA DESCRIPTION

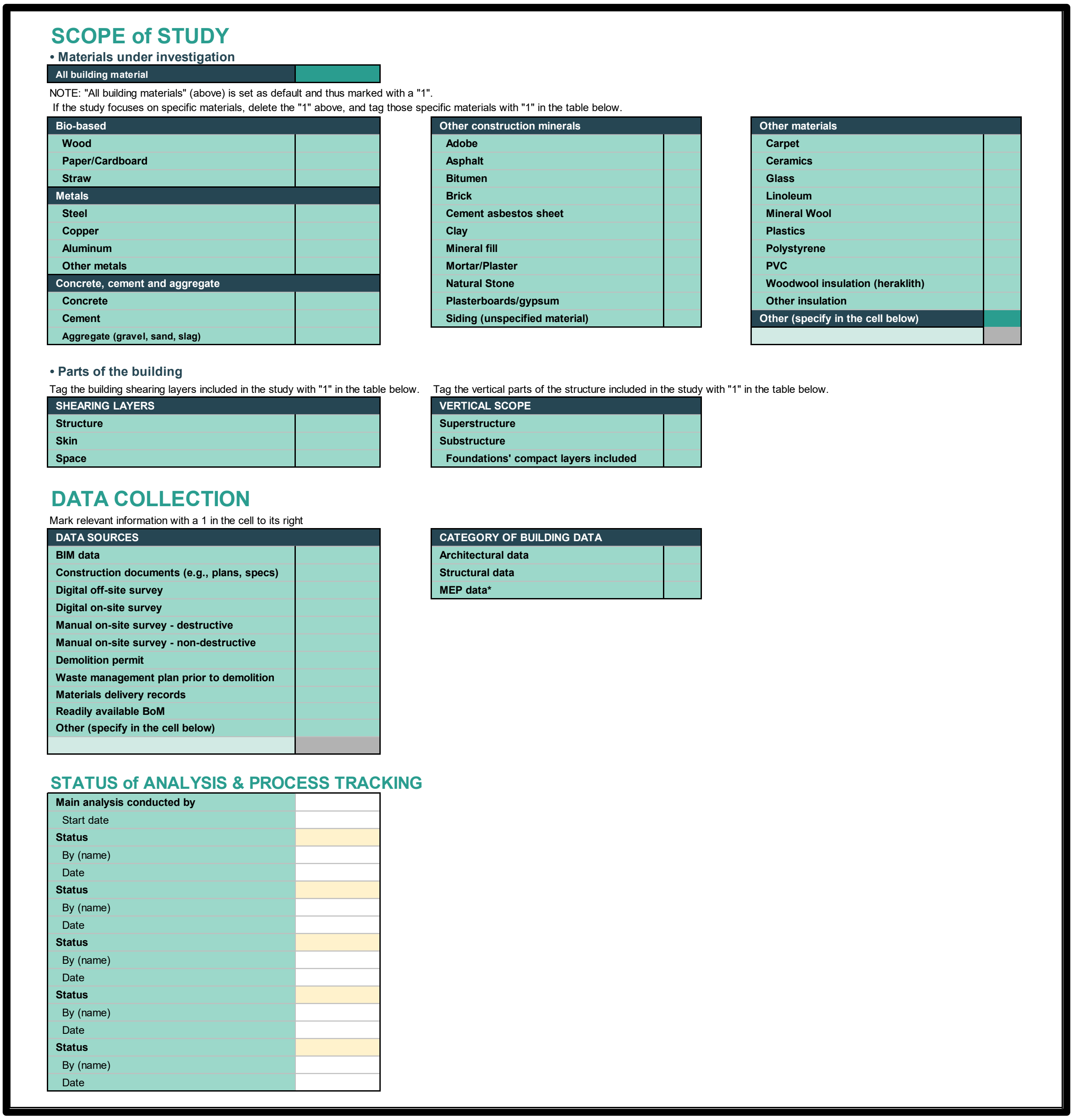

SCOPE of STUDY

• Materials under investigation

All building material	

NOTE: "All building materials" (above) is set as default and thus marked with a "1".
If the study focuses on specific materials, delete the "1" above, and tag those specific materials with "1" in the table below.

Bio-based	
Wood	
Paper/Cardboard	
Straw	
**Metals**	
Steel	
Copper	
Aluminum	
Other metals	
**Concrete, cement and aggregate**	
Concrete	
Cement	
Aggregate (gravel, sand, slag)	

Other construction minerals	
Adobe	
Asphalt	
Bitumen	
Brick	
Cement asbestos sheet	
Clay	
Mineral fill	
Mortar/Plaster	
Natural Stone	
Plasterboards/gypsum	
Siding (unspecified material)	

Other materials	
Carpet	
Ceramics	
Glass	
Linoleum	
Mineral Wool	
Plastics	
Polystyrene	
PVC	
Woodwool insulation (heraklith)	
Other insulation	
**Other (specify in the cell below)**	

• Parts of the building

Tag the building shearing layers included in the study with "1" in the table below.

SHEARING LAYERS	
Structure	
Skin	
Space	

Tag the vertical parts of the structure included in the study with "1" in the table below.

VERTICAL SCOPE	
Superstructure	
Substructure	
Foundations' compact layers included	

DATA COLLECTION

Mark relevant information with a 1 in the cell to its right

DATA SOURCES	
BIM data	
Construction documents (e.g., plans, specs)	
Digital off-site survey	
Digital on-site survey	
Manual on-site survey - destructive	
Manual on-site survey - non-destructive	
Demolition permit	
Waste management plan prior to demolition	
Materials delivery records	
Readily available BoM	
Other (specify in the cell below)	

CATEGORY OF BUILDING DATA	
Architectural data	
Structural data	
MEP data*	

STATUS of ANALYSIS & PROCESS TRACKING

**Main analysis conducted by**	
Start date	
**Status**	
By (name)	
Date	
**Status**	
By (name)	
Date	
**Status**	
By (name)	
Date	
**Status**	
By (name)	
Date	
**Status**	
By (name)	
Date	

Figure 32 Overview of the tab "**Scope and data**" in BUD-MI.

2.1. Scope of study

Defining the scope of the study is crucial. Indeed, a well-defined scope ensures that the study's results are comparable to other similar studies, enhancing the reliability and relevance of the findings. In BUD-MI, the scope is defined according to two key aspects: which materials are under investigation, and which part(s) of the building are included in the analysis.

2.1.1. Materials under investigation

• Materials under investigation

All building material	1

NOTE: "All building materials" (above) is set as default and thus marked with a "1".
If the study focuses on specific materials, delete the "1" above, and tag those specific materials with "1" in the table below.

Bio-based	
Wood	1
Paper/Cardboard	1
Straw	1
**Metals**	
Steel	
Copper	
Aluminum	
Other metals	
**Concrete, cement and aggregate**	
Concrete	
Cement	
Aggregate (gravel, sand, slag)	

Other construction minerals	
Adobe	
Asphalt	
Bitumen	
Brick	
Cement asbestos sheet	
Clay	
Mineral fill	
Mortar/Plaster	
Natural Stone	
Plasterboards/gypsum	
Siding (unspecified material)	

Other materials	
Carpet	
Ceramics	
Glass	
Linoleum	
Mineral Wool	
Plastics	
Polystyrene	
PVC	
Woodwool insulation (heraklith)	
Other insulation	
**Other (specify in the cell below)**	

Figure 33 "**Materials under investigation**" fields in BUD-MI.

The default setting assumes that all materials are under investigation ("All building materials" is tagged with 1 (one)). This doesn't imply that each listed material is present in the building, but rather that any materials found will be included in the analysis.

If not all materials are being investigated, tag "All building materials" with 0 (zero). Then, in the list of materials, tag each investigated material with 1 (one).

2.1.2. Parts of the building

• Parts of the building

Tag the building shearing layers included in the study with "1" in the table below.

SHEARING LAYERS	
Structure	
Skin	1
Space	

Tag the vertical parts of the structure included in the study with "1" in the table below.

VERTICAL SCOPE	
Superstructure	1
Substructure	1
Foundations' compact layers included	1

Figure 34 "**Parts of the building**" fields in BUD-MI.

- *Building shearing layers*

Tag the building shearing layers included in the study with 1 (one). For more information on building shearing layers, see Section 3.1.4 of this chapter.

- *Vertical scope*

Tag the vertical parts of the structure included in the study with 1 (one). For more information on vertical scope, see Section 3.1.2 of this chapter.

2.2. Data collection

Describing data sources ensures transparency, facilitates replication, and clarifies the context and applicability of the analysis. In BUD-MI, the data is described in two respects: its source, and its type.

2.2.1. Data sources

Building data comes in many forms, of which the most common are listed (Figure 35). Tag the source(s) of building data used in the study with 1 (one).

A short description of each type of data source is given in Table 13 below.

Mark relevant information with a 1 in the cell to its right

DATA SOURCES	
BIM data	
Construction documents (e.g., plans, specs)	1
Digital off-site survey	1
Digital on-site survey	
Manual on-site survey - destructive	
Manual on-site survey - non-destructive	
Demolition permit	
Waste management plan prior to demolition	
Materials delivery records	
Readily available BoM	
Other (specify in the cell below)	

Figure 35 “**Data sources**” fields in BUD-MI. Acronyms: BIM Building Information Modeling, BoQ Bill of Quantity, specs specifications.

Table 13 Short description of each type of data source. Acronyms: BIM Building Information Modeling, BoQ Bill of Quantity.

Data source	Short description
BIM data	Information generated and managed in BIM (e.g., Revit).
Construction documents (e.g., plans, specs)	Drawings (e.g., floorplans, sections) and specifications of the building. For older buildings, those are hand drawn and written.
Digital off-site survey	Data collected using digital tools (e.g., drones or satellite imagery) without physical presence.
Digital on-site survey	Data collected at the building location using digital tools (e.g., laser scanners, 3D imaging).
Manual on-site survey – destructive	Data collected through physical inspection involving partial demolition or removal of building materials.
Manual on-site survey – non-destructive	Data collected through inspection without altering or damaging the structure or its materials.

Demolition permit	Official authorization required to legally demolish a building or structure. Some include a list of expected materials.
Waste management plan prior to demolition	A strategy outlining the handling, recycling, and disposal of materials before a demolition project begins.
Materials delivery records	Documentation tracking the receipt and details of construction materials delivered to a project site.
Readily available BoQ	A document listing all materials, products, and labor needed for a construction project.
Other	Specify what other data source is used.

2.2.2. Category of building data

Building data can be categorized into three main categories: architectural data (focused on design and spatial elements), structural data (load bearing and stability components), and MEP data (mechanical, electrical, and plumbing systems). Each category follows its own drawing conventions and terminology and is managed by different types of engineers or specialists.

Clearly specifying which category or categories of building data are used for analysis is essential for ensuring transparency in data collection. For instance, if architectural data is used, structural materials that have limited relevance to architectural design may not be described in detail and therefore require assumptions. A common example is the depth of a foundation, which is rarely detailed in architectural data.

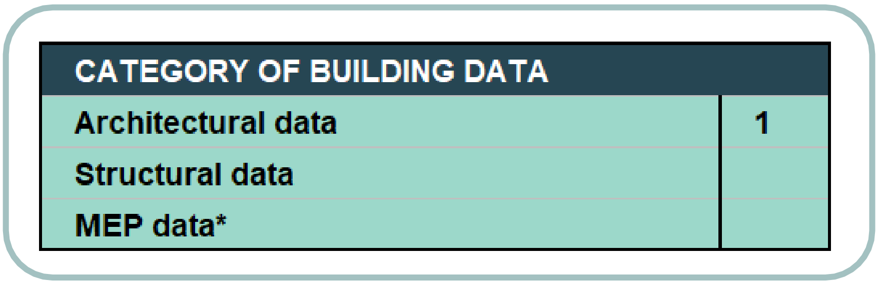

Figure 36 "**Category of building data**" fields in BUD-MI.

- *Architectural data*

Drawings that depict the overall design and layout of the building. They include floor plans, elevations, sections, and details that show the arrangement of spaces, dimensions, materials, finishes, doors, windows, and other architectural elements. They serve as the blueprint for how the building will look and function.

- *Structural data*

Focused on the integrity and safety of the building's framework, they detail how the building will be supported and resist forces such as gravity, wind, and seismic activity. They include information on the building's foundation, beams, columns, slabs, and other structural components. They specify the materials, sizes, and connections of these elements, ensuring the building is stable and secure.

- *MEP data*

Mechanical, Electrical, and Plumbing (MEP) plans cover the essential systems that make the building livable and functional. They ensure that the mechanical, electrical, and plumbing systems are properly designed, coordinated, and integrated within the structure. They include mechanical

plans (incl. HVAC, ductwork, and the like), electrical plans (incl. wiring, lighting, and the like), and plumbing plans (incl. water supply, drainage, and the like).

2.3. Status of analysis

It is beneficial for MI data to be verified by one or more people to ensure that assumptions are reasonable. This review is especially important when data is collected by multiple people, as it is essential that one person ensures consistency across all the collected MI data.

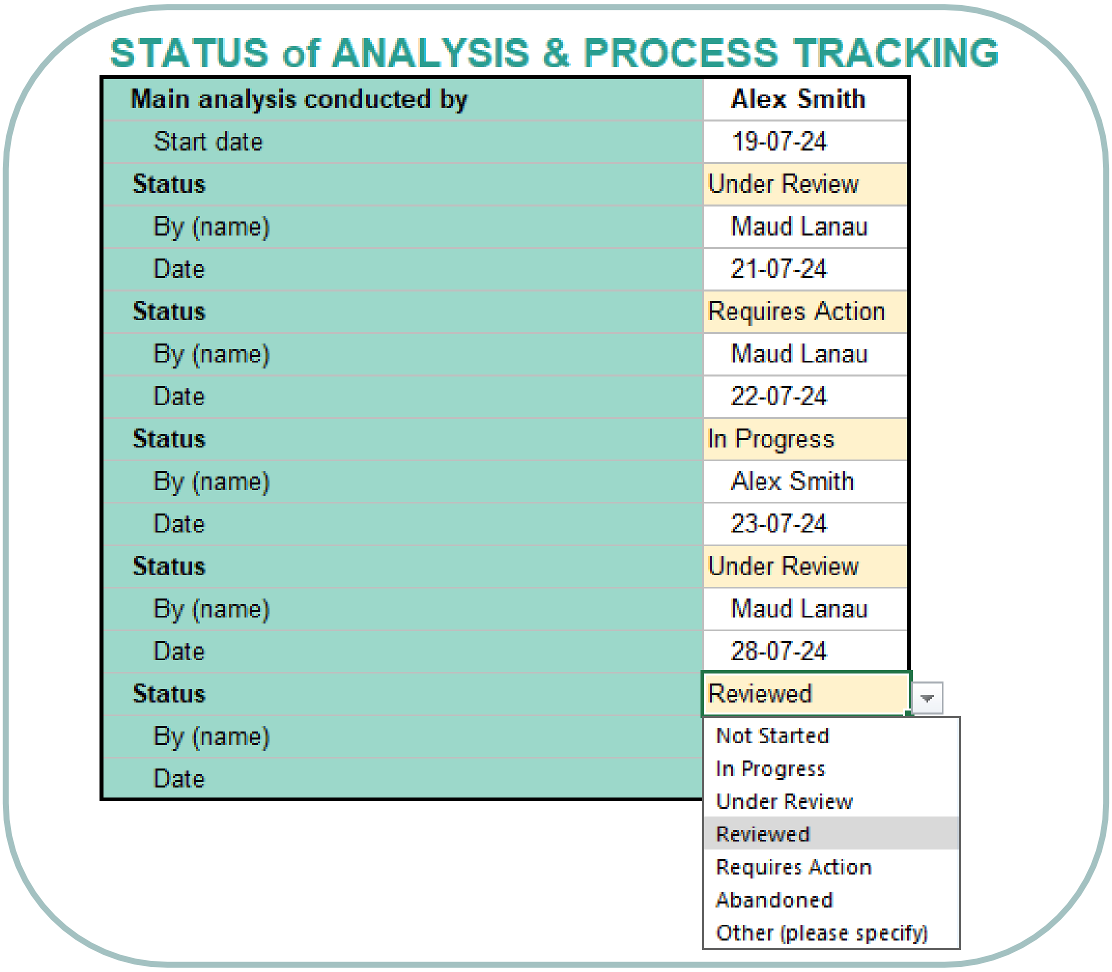

Figure 37 "**Status of analysis & process tracking**" data fields in BUD-MI.

Enter the names of data collectors/reviewers and the corresponding dates. Tracking the data collection progress promotes transparency and accountability and facilitates quality control.

Table 14 Predefined status of analysis in BUD-MI.

Status	Description
**Not Started**	No work has begun.
**In Progress**	Data collection underway.
**Under Review**	Quality checks ongoing.
**Reviewed**	Reviewed and approved.
**Requires Action**	Corrections needed.
**Abandoned**	Work stopped permanently.
**Other (please specify)**	For unique cases not covered by the predefined statuses.

3. Bill of Material

Crux of the data collection, the Bill of Material tab is where each building's item is characterized in terms of dimensions and material, so the item's weight and apparent volume may be automatically calculated. The tab is divided into four main four blocks: Item description, item's dimension, item's material, and sub-result, i.e., item's weight and apparent volume.

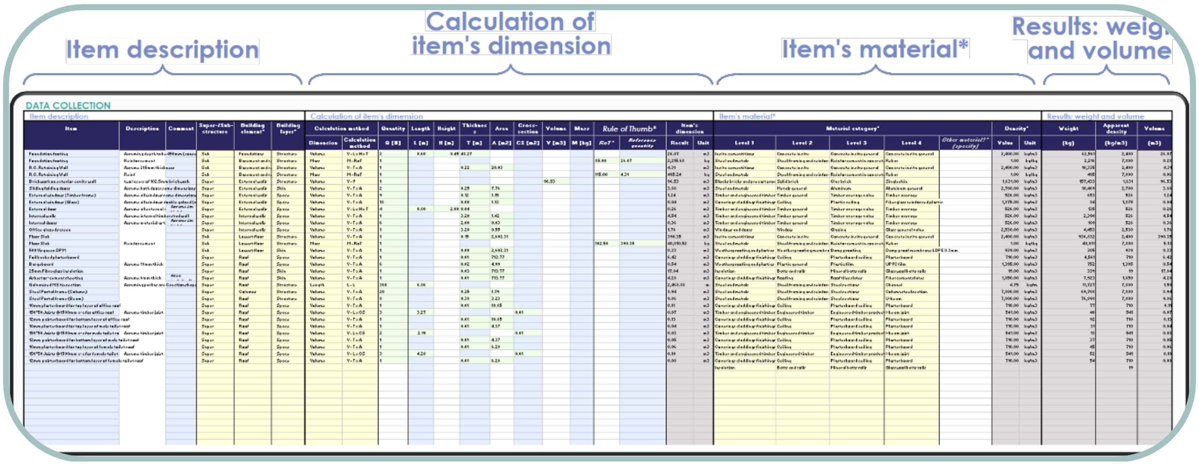

Figure 38 Overview of the tab "**Bill of material**" in BUD-MI.

3.1. Item's description

In item's description, users enter a description of each item in the building. In addition to manually entering information (in the manual entry fields "item", "description", and "comment")

Item description

Item<br>*e.g., "Foundation footing", "External door", "Floor slab", "Roof cover", …*	Description<br>*e.g., "Foundation: concrete", "Foundation: reinforcement", "Window: frame", "Window: glass", …*	Comments/assumptions<br>*e.g., "thickness assumed as 215mm"; "unspecified material: assumed timber"*	Super/Sub	Building element	Shearing layer
Foundation footing	Reinforced concrete	Assumed depth: 450mm	Sub	Foundations	Structure
Retaining wall	Reinforced concrete	Assumed thickness 215mm	Sub	Basement and ret	Structure
Brickwork on exterior cavity wall	Two layers of 102.5mm brickwork		Super	External walls	Structure
Sliding folding doors		Assumed: both doors same dimensions, in metal	Super	External walls	Skin
External door		Assumed 2m high (no drawing on elevation)	Super	External walls	Space

Figure 39 Example of **item descriptions** in BUD-MI. The location of each item in the building is specified in terms of vertical location (super- or sub-), building element, and shearing layer.

3.1.1. Short description of each item

Three text fields are available for users to provide detailed information about each building item.

- In "Item", identify the specific building component or element being described. Includes the name or label of the item, such as "Window", or "Beam".
- In "Description", provide a detailed explanation of the item, such as its technical specifications, its material, and any other relevant details about the item.
- In "Comment", add any relevant additional information. Include also any assumptions, issues, recommendations, or additional context that may be useful for understanding or evaluating the item.

3.1.2. Super/sub

The delineation between sub and super is visualized in Figure 40. The scope of each is described as follows:

- The "sub" part of the building includes all the structural work underground. This includes the lowest floor slabs and its waterproofing and insulation. In the presence of basement, the substructure includes the basement's sides and bottom slab, as well as their waterproofing and insulation.
- The "super" part of the building includes all the rest. In the case of basements, this includes the basement's upper floors.

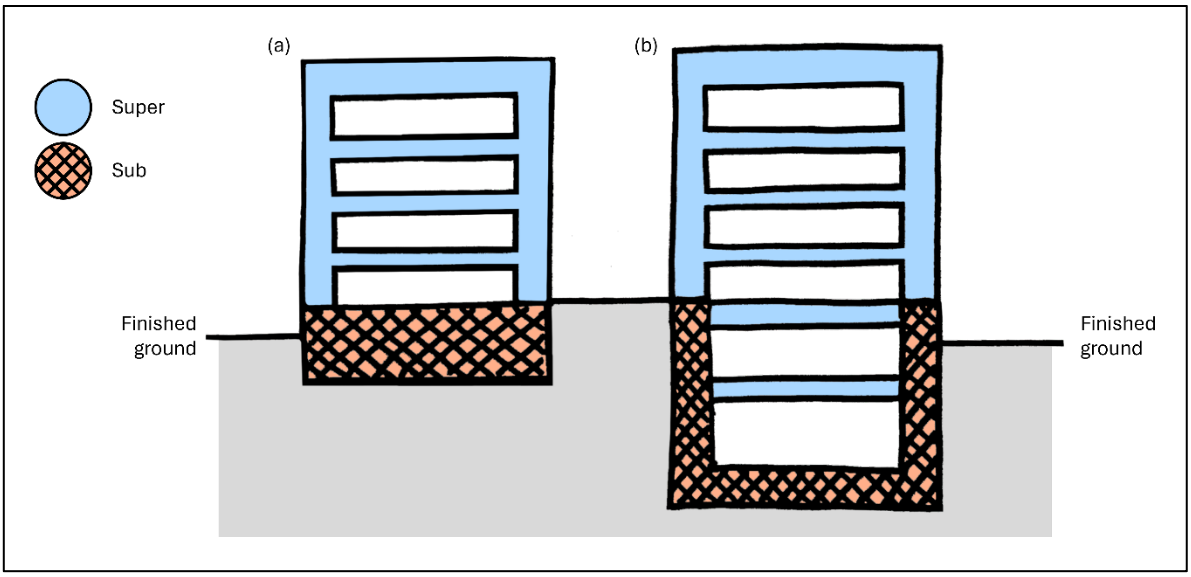

Figure 40 Delineation between sub- and super-structures for a building (a) without and (b) with basement. Note that the level of finished ground corresponds to the lowest level of ground around the building. Adapted from (ICMS 2021)

3.1.3. Elemental breakdown

The building elemental breakdown depicted in Figure 41 shows each building elements used in BUD-MI. It is based on the elemental breakdown used in the New Rules of Measurements 1 (NRM1) by the Royal Institute of Chartered Surveyors (RICS, 2021).

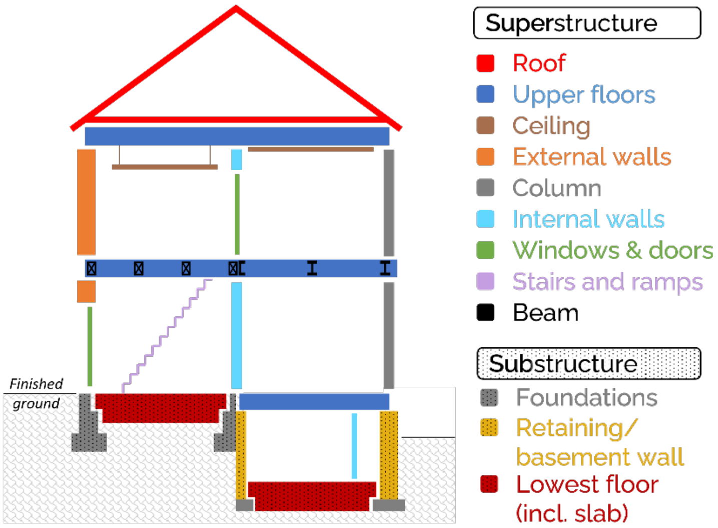

Figure 41 **Elemental breakdown** of a building.

3.1.4. Building shearing layer

Users choose the building shearing layer in which the item is located.

Note that only three out of six building shearing layers are included in BUD-MI, namely the skin, structure, and space of the building. The choice to exclude the layers “service” and “stuff” was based on the prevailing practice in MI data collection. The “service” layer may be added to BUD-MI in the future, however.

- *About building shearing layers*

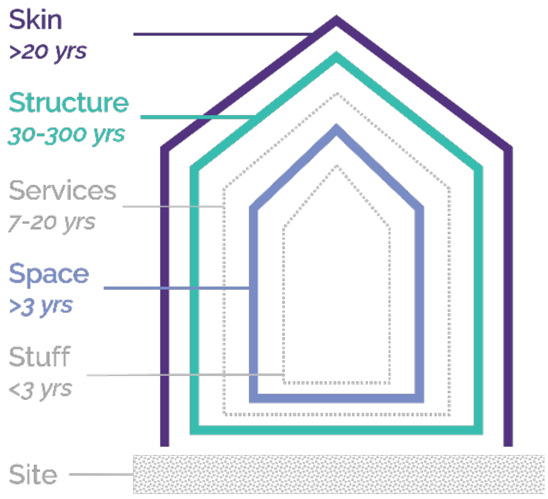

BUILDING SHEARING LAYERS

"Our basic argument is that there isn't any such thing as a building. A building properly conceived is several layers of longevity of built components." (Brand, 1994)

Figure 42 **Building shearing layers**. (Brand, 1994) Note that shearing layers in grey are not included in BUD-MI.

Table 15 Building shearing layers, their definition, and their typical lifetime. (Brand, 1994; ICMS Coalition, 2021; Pushkar, 2015) Note that shearing layers in grey are not included in BUD-MI.

Shearing layer	Definition	Typical lifetime (years)
**Site**	Location and context: Excavation and landfill	Permanent
**Structure**	Bones of the building, including foundations, frame (columns and beam, frame wall), load-bearing elements, structural slabs, **and the waterproofing and insulation integrated within them.**	30-300
**Skin**	Building's envelope, including external walls (non-load bearing), external wall covering, roofing, glazing, and the like.	20-50
**Services**	HVAC, electrical, plumbing, telecommunication fixtures	7-20
**Space Plan**	Interior layout, including partition walls, non-bearing internal walls, internal doors, wall finishes, flooring finishes, ceilings, and the like.	3-10
**Stuff**	Furniture and equipment, e.g., computers, furniture, light bulbs, etc.	0^{+}-3

- *Elemental allocation of ambiguous items*

For some building items, allocation to one layer instead of another can be ambiguous. While an overview of the most common allocation is provided in Figure 43, a few items require careful consideration. Examples of such ambiguous items are provided below, though the list is non-exhaustive. Additional ambiguous examples might be added to this User Guide in the future.

- **Insulation**. If integrated into the building structure, it should be allocated to the *structure layer*. This is because it cannot be retrieved without affecting the building's structure. (ICMS Coalition, 2021) If not integrated into the structure, it should be allocated to the skin layer.

- **Waterproofing** (e.g., membranes). Similarly to insulation, if integrated into the building structure, waterproofing membranes should be allocated to the *structure layer*. This is because it cannot be retrieved without affecting the building's structure. (ICMS Coalition, 2021) If not integrated into the structure, it should be allocated to the skin layer.
- **Retaining wall**. Retaining walls should be allocated to the *structure* of the building.

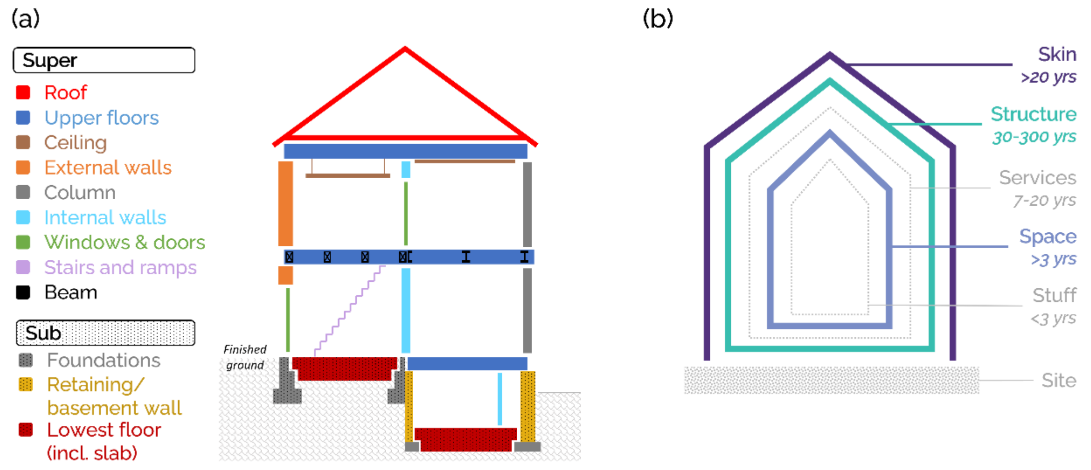

(c)

Building shearing layers

Elemental breakdown	STRUCTURE	SPACE	SKIN
SUPER			
Beams	Frame beams	Non-structural beam (e.g., faux beams)	-
Columns	Frame columns	Non-structural column (e.g., pilaster)	-
Ceiling	-	Ceiling finishes	-
External walls	Frame casing<br>Loadbearing external walls<br>Wall insulation*	Wall finishes (inside)	External enclosing walls<br>Wall veneers/cladding<br>Wall insulation*
Internal walls	Loadbearing internal walls	Partition walls<br>Wall finishes	-
Roof	Roof structure	In lofts: internal and knee walls	Roof coverings<br>Roof insulation
Stairs and ramps	Stairs (structural)	Stairs, ramps, balustrades	-
Upper floors	Slabs	Floor finishes	-
Windows and external doors	-	Internal doors	Windows, external doors
SUB			
Foundations	Strip foundations, piles	-	Foundation veneer
Retaining wall (incl. basement's)	Basement retaining walls		-
Lowest floor	Slab, raft foundations<br>Slab insulation*	Lowest floor finishes	-

**Building components that cannot be retrieved without affecting the building structure (e.g., insulation in a sandwich wall, slab insulation) should be allocated to the structure layer.*

Figure 43 Allocation of components with regards to shearing layers, elemental breakdown, and vertical delineation.

3.2. Calculation of item's dimension

Calculation of item's dimension

Calculation method		Quantity	Length	Height	Thickness	Area	Cross-section	Volume	Mass	*Rule of Thumb**		Item's dimension	
Dimension	Calculation method	Q [#]	L [m]	H [m]	T [m]	A [m2]	CS [m2]	V [m3]	M [kg]	*RoT**	*Reference quantity*	Result	Unit
Volume	V = L x H x T	2	0.60	0.45	48.27							26.07	m3
Mass	M = RoT	1								85.00	26.07	2,215.68	kg
Volume	V = T x A	1			0.22	20.03						4.31	m3
Mass	M = RoT	1								115.00	4.31	495.24	kg
Volume	V = V	1						96.53				96.53	m3

Figure 44 Example of "Calculation of **item dimension**" in BUD-MI.

3.2.1. Target dimension and calculation method

Under "**target dimension**", select the key dimension in which the element will be characterized: volume, area, length, or mass.

Under "**calculation method**", select how to calculate the dimension. This choice should be based on available information on the item and their own judgment.

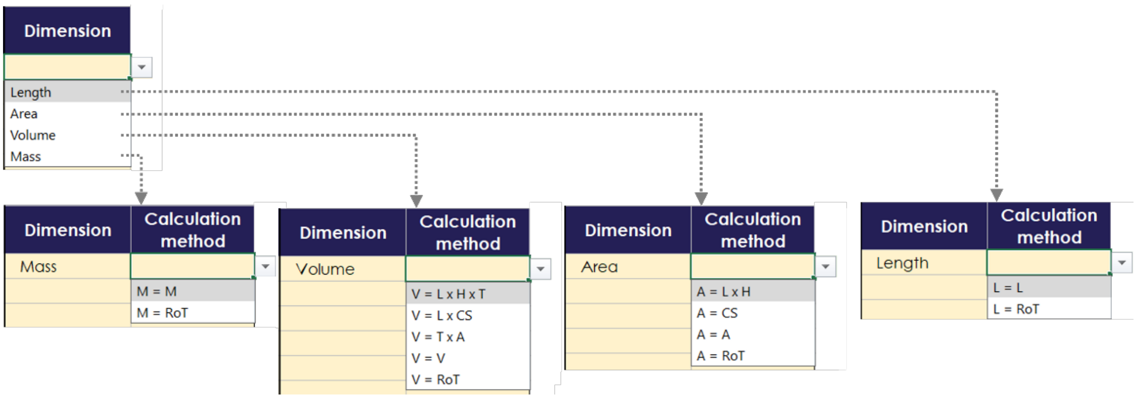

Figure 45 Depending on the target dimension, the calculation method will differ. See Table 16 for acronym definitions.

Table 16 Acronyms used in "calculation method". Acronym: n.a., not applicable.

Acronym	Full form	Unit
**Q**	Quantity	#
**L**	Length	m
**H**	Height	m
**T**	Thickness	m
**A**	Area	m2
**CS**	Cross Section	m2
**V**	Volume	m3
**M**	Mass	Kg
**RoT**	Rule of Thumb	n.a.

3.2.2. Examples

In the examples below, a differentiation is made between items with **high aspect ratio** and those with **low aspect ratio**.

High aspect ratio refers to items whose length is much greater than the cross-sectional dimensions, such as steel members, columns, piles, and more. Low aspect ratio refers to item whose width and thickness are more comparable, such as slabs, windows, walls, and more.

- ***Length** as target dimension*

Length (L) can be used as a target dimension for all items for which a linear density (in kg/m) is available in BUD-MI or easily retrievable online. This is often the case for items with high aspect ratio, such as steel sections and timber members.

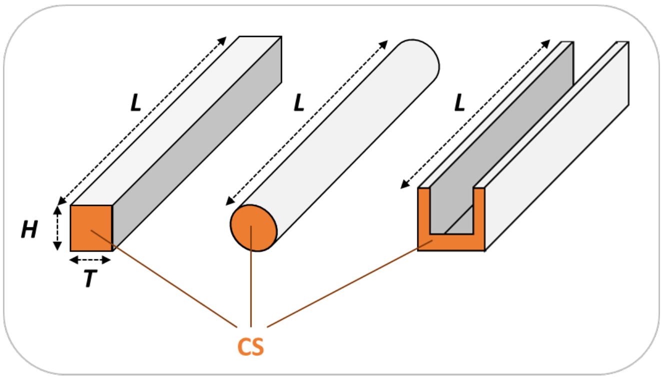

Known parameters	Calculation method
L	L = L
None, but depending on other known parameters	L = RoT (See 3.2.4 in this chapter)

Figure 46 Parameters that may be used to calculate the length of items with high aspect ratio.

- ***Area** as target dimension*

Area (A) can be used as a target dimension for all items for which an areal density (in kg/m2) is available in BUD-MI. However, if the thickness of the item is known, volume (V) can also be used as target dimension. This choice should be made according to available information and to the item in question.

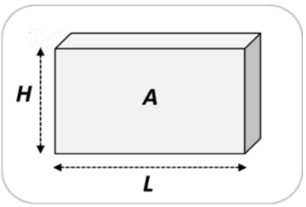

Known parameters	Calculation method
H, L	A = L x H
A	A = A
None, but depending on other known parameters	A = RoT (See 3.2.4 in this chapter)

Figure 47 Parameters that may be used to calculate the area of items with low aspect ratio.

An example of item for which areal density exists is that of plastic membrane.

- ***Volume** as target dimension*
    - Calculating the **volume** of a wall, window, door, slab, panel, and the like.

Known parameters	Calculation method
T, H, L	V = H x L x T
T, A	V = A x T

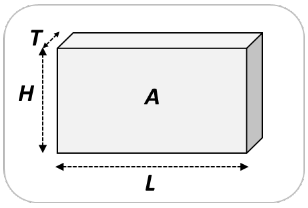

None, but depending on other known parameters	V = RoT (See 3.2.4)

Figure 48 Parameters that may be used to calculate the volume of items with low aspect ratio.

- Calculating the **volume** of a pile, column, steel member, and the like.

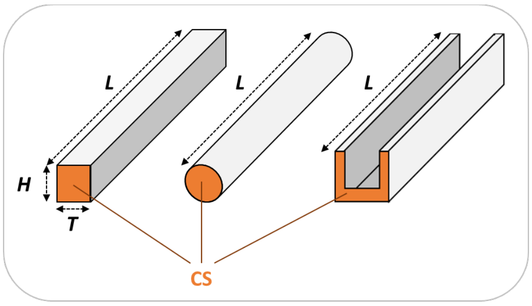

Known parameters	Calculation method
CS, L	V = CS x L
None, but depending on other known parameters	V = RoT (See 3.2.4)

Figure 49 Parameters that may be used to calculate the volume of items with high aspect ratio.

⚠ For steel and timber members, the preferred target dimension should be length (L), as linear mass data (i.e., in kg/m) is available in BUD-MI, or easily retrievable online.

- ***Mass** as target dimension*

In some instances, the mass of the item is known. If the mass is known because it is stated in building data, users can simply select "M=M" and enter the mass of the item is the relevant column.

However, if the mass is to be calculated from a Rule of Thumb (e.g., mass of steel in a specific volume of reinforced concrete), then the user must select "M = RoT". An example of the corresponding calculation is given in Section 3.2.4 of this chapter.

Known parameters	Calculation method
M	M = M
None, but depending on other known parameters	M = RoT (See 3.2.4 in this chapter)

3.2.3. Enter dimensions of the item

After choosing the calculation method, the corresponding cells to be filled will be highlighted in green (see Figure 50 below). Fill those with the relevant information.

⚠ Always enter the quantity of items (Q).

⚠ For Rules of thumb, see subsequent section and Chapter II – Section 1.

Calculation of item's dimension

Calculation method		Quantity	Length	Height	Thickness	Area	Cross-section	Volume	Mass	*Rule of Thumb**	
Dimension	Calculation method	Q [#]	L [m]	H [m]	T [m]	A [m2]	CS [m2]	V [m3]	M [kg]	*RoT**	*Reference quantity*
Length	L = L										
Length	L = RoT										
Area	A = L x H										
Area	A = CS										
Area	A = A										
Area	A = RoT										
Volume	V = L x H x T										
Volume	V = L x CS										
Volume	V = T x A										
Volume	V = V										
Volume	V = RoT										
Mass	M = M										
Mass	M = RoT										

Figure 50 Screenshot showing the highlighting of cells to fill in depending on the chosen method of dimension calculation.

3.2.4. Rules of thumb

When information on a building item is insufficient, rules of thumb (RoT) can be used. The "Rule of Thumb" columns in the BoM tab includes two pieces of information (RoT value and reference quantity). Always make sure to fill in both information – BUD-MI multiplies these two to calculate the dimension of the item.

- The RoT value can be retrieved from the relevant section in the "Rule of Thumb" tab (see Chapter II – Section 1). Each RoT is expressed per unit of reference quantity.
- The reference quantity pertains to the quantity of building items being assessed.
- Again, also make sure to state Q.

The example below illustrates how to use rules of thumb in BUD-MI.

EXAMPLE: STEEL IN REINFORCED CONCRETE

Case: a slab is made of reinforced concrete (RC). The building plan provides its dimension (5m x 10m x 0.2m), but there is no information on the quantity of steel. A rule of thumb must be used.

Step 0 – Calculating the volume of RC (if not already done)

In BoM, make sure to have calculated the volume of the slab.

- Volume V = H x L x T
- Q = 1

➔ Result: 10 m3 of reinforced concrete)

Step 1 – Retrieving the rule of thumb

In the tab "Rules of Thumb", check the available rules of thumb – luckily, there is one for steel reinforcement in RC! Indeed, quantities of steel reinforcement can be approximated based on the volume of RC and its use (e.g., column, slab, beam, pile).

Enter the required information:

- Type of concrete element > slab
- Specify further > general slab

➔ Returned value: 103 kg.steel/m3.RC

This means that the mass of steel can be calculated based on the volume of the slab.

Step 2: Using the rule of thumb

In BoM, set up your calculation.

- calculation method
  - target dimension > Mass
  - calculation method > M = ROT
- Q = 1
- Rule of thumb
  - RoT: 103
  - Reference quantity: 10 (i.e., 10 m3, the volume of the slab previously calculated in Step 0).

➔ Returned value: 1030 kg.steel

Tipbox 1 Example of using a rule of thumb in BUD-MI.

3.2.5. Dimension result

The last column of the "Calculation of item dimension" information block is the dimension of the item. This dimension must be aligned with the *target dimension* selected in the eponymous column (see 3.2.1 in this chapter), and to the measurements entered.

The dimension result returned here is later multiplied by the density of the item's material (See 3.3.2 of this chapter) to calculate the item's weight and volume.

3.3. Item's material

3.3.1. Material selection

The selection of material allows (1) to retrieve the density of the material, and (2) to automatically assign the material to its material category, which is then used to generate MI results.

Nevertheless, new materials and their densities **can be inputted manually**. Descriptions on how to do so can be found in Chapter V.

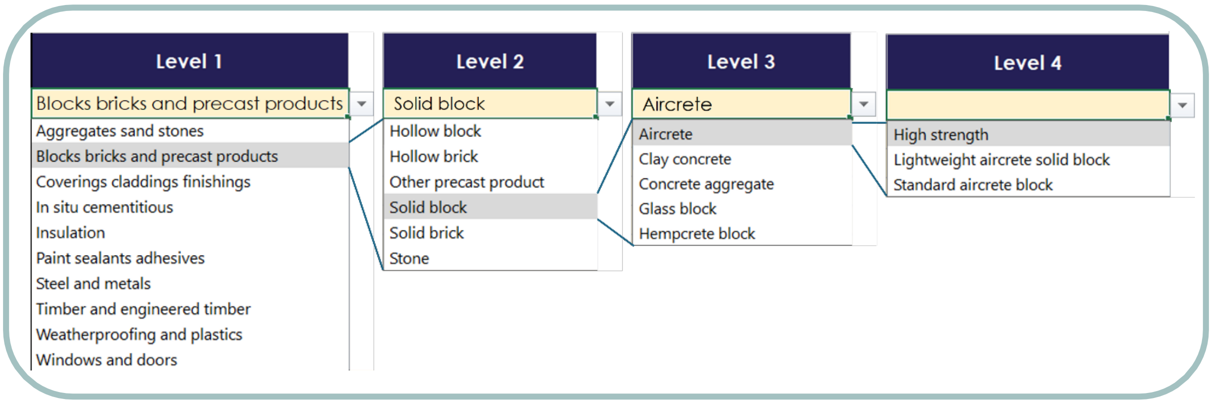

Figure 51 Selection of high-strength aircrete solid block using BUD-MI's nested dropdowns lists.

Construction materials used in BUD-MI are sorted into a tailored classification developed with the expressed goals on facilitating material finding selection for several user profiles. The classification can be found in tabs "Search materials", "Crossmatch material classific.", and in Annex 1.

To further facilitate an efficient selection of material type, materials can be found in several places across the classification. The user may then rely on their background to locate the material in the nested dropdowns. For example, an architect may tend towards the function of an item (e.g., coverings) to find a material (e.g., Coverings > … > Timber sheet > Timber average), while an industrial ecologist or MFA practitioner may use a material-based logic (e.g., Timber and timber products > … > Timber average).

In any case, and again with the aim to facilitate material location and selection, the tab "Search material" can be used to find a material within the classification. More information can be found in Chapter II – Section 2.

3.3.2. Density

The selection of the material returns its density. The target dimension chosen to describe the item (see 3.2.1) sets the unit in which the material density is retrieved. Depending on the target dimension (m3, m2, m, or kg), BUD-MI retrieves the material density (volumic density, areal density, linear density, or 1, respectively). In cases where the density is not available in the unit stated as a target dimension (e.g., kg/m2), an error appears (e.g., "please specify dimension in

m3”). In such cases, users can manually input the density if they have it available; otherwise, an alternative target needs to be selected.

3.4. Item’s weight and volume

Results on the item’s weight and volume are shown as a mid-result, helping the user understand the scale of the material dimension and mass, but also helping spot any aberrant data input.

3.4.1. Weight, volumic density, and volume

After having filled the calculation method (section 3.2.1 of this chapter3.2), the item’s dimension (section 3.2.3 of this chapter), and the item’s material (section 3.3 of this chapter), BUD-MI returns the resulting weight and volume of the item.

These results, in combination with the building’s information (section 1.4 of this chapter) are used to generate MIs in various units and formats (see Chapter III. Understanding & Generating Results).

3.4.2. Material type

In column [AF], each item is automatically assigned a material type used to display a summary of result in the tab “Results – summary”. This material classification, based on Heeren and Fishman MI database (Heeren & Fishman, 2019), can be found in Annex 1.2.

Chapter II.
MINI TOOLS

Several mini tools are included in BUD-MI to help the user. Those are described succinctly in the next pages.

1. RULES OF THUMB

Rules of thumb

Rules of thumb are used when information is missing in building plans and are the base of the mini-tools below.
The purpose of these mini-tools is to help the user filling in the Sheet "Bill of Material".
Close attention should be paid to the unit in which results are expressed in each mini-tools, so that the relevant calculation method is selected in the "Bill of Material".

Mass of steel reinforcement per cubic meter of reinforced concrete

Select the type of concrete element to which the reinforcement belongs.

Concrete element:	
Specify further:	
Value (kg.steel/m3.RC)	

Linear density of steel sections

The linear density of typical steel sections can be found in the link below. (Last accessed: 20th Sept 2023)

Interactive 'Blue Book'

Triple-S Steel - Metals reference guide

Mass of materials per square meter of window

Use the relevant calculator below, depending on the window frame under study

Aluminum frame

Glazing configuration	
aluminum (kg/m2.window)	
glass (kg/m2.window)	

PVC frame

Glazing configuration	
PVC (kg/m2.window)	
steel (kg/m2.window)	
glass (kg/m2.window)	

Timber frame

Glazing configuration	
Timber (kg/m2.window)	
glass (kg/m2.window)	

See also Parkglass' glass weight calculator here

Volume distribution in masonry

Specify the type of masonry (block or brick) below, and enter the total volume of masonry.

Type	
Volume of masonry (m3)	
Volume Mortar (m3)	
Volume (m3)	

Studs in walls and ceilings

Total length of studs in plaster walls

Enter length and height of plaster wall below.
Note: c-c is the distance between studs center-to-center, typically around 0.4m.

Wall length (m)	
Wall height (m)	
c-c (m)	
Linear meters of studs (m)	

Studs in ceiling, external, and light walls (kg/m2)

Material	
System	
kg/m2.system	

Calculation of roof area

Areas of hip, gable, and shed roofs can be calculated using the link below (last accessed: 20th Sept 2023)

Roof area calculator

Engineered wood products - self weight

Linear density of common engineered wood products can be found in the link below (last accessed: 01 March 2024).
Please note the units are in pounds per feet, and need to be converted to the International System of Units.

Weyerhaeuser engineered lumber

Figure 52 Overview of the tab "**Rules of Thumb**" in BUD-MI

Rules of thumb (RoT) can be defined as practical procedures, approaches, or principles to measuring, calculating, or doing something approximately, based on experience or practice.

In BUD-MI, ROTs help estimate information otherwise missing in building plans. All RoTs are gathered in the tab “rules of thumb”.

When using RoTs in the BoM, two pieces of information are needed: RoT value and the reference quantity. Always make sure to fill in both information – BUD-MI multiplies these two to calculate the dimension of the item.

- The RoT value can be retrieved from the relevant section in the “Rule of Thumb” tab. Each RoT is expressed per unit of reference quantity.
- The reference quantity pertains to the quantity of building item being assessed.
- Again, also make sure to state Q.

Attention should be paid to the unit in which results are expressed in each mini tool, so that the relevant calculation method is selected in the "Bill of Material".

An example of how to use a RoT is provided in Chapter I – Section 3.2.4.

2. Search for Materials

The material categorization used in BUD-MI can be consulted below.

Use the filter function or [CTRL+F] to find a specific material and see to which category it belongs.

Level 1	Level 2	Level 3	Level 4	Description and synonyms
Aggregates sand stones	Aggregates	Aggregates general	Aggregates and sand general	Aggregates, sand
Aggregates sand stones	Aggregates	Aggregates general	Coarse aggregate general	Aggregates, coarse
Aggregates sand stones	Aggregates	Crushed aggregate	Crushed asphalt compact	Asphalt, crushed, compact
Aggregates sand stones	Aggregates	Crushed aggregate	Crushed asphalt loose	Asphalt, crushed, loose
Aggregates sand stones	Aggregates	Crushed aggregate	Crushed clay brick coarse	Clay, brick, crushed, coarse
Aggregates sand stones	Aggregates	Crushed aggregate	Crushed clay brick fine	Clay, Brick, Fine, Crushed
Aggregates sand stones	Aggregates	Crushed aggregate	Crushed concrete	Concrete, crushed
Aggregates sand stones	Aggregates	Crushed aggregate	Crushed dolomite	Dolomite, crushed
Aggregates sand stones	Aggregates	Crushed aggregate	Crushed mixed base compact	Crushed, mixed base, compact
Aggregates sand stones	Aggregates	Crushed aggregate	Crushed mixed base loose	Crushed, mixed base, loose
Aggregates sand stones	Aggregates	Crushed aggregate	Gypsum crushed	Gypsum, crushed
Aggregates sand stones	Aggregates	Crushed aggregate	Pumice aggregates	Pumice, aggregates
Aggregates sand stones	Aggregates	Crushed aggregate	Stonechips	Stonechips
Aggregates sand stones	Aggregates	Expanded materials	Expanded clay	Clay, expanded
Aggregates sand stones	Aggregates	Expanded materials	Expanded clay clinker	Clay, expanded, clinker
Aggregates sand stones	Aggregates	Expanded materials	Expanded glass	Aggregates, recycled, glass
Aggregates sand stones	Aggregates	Expanded materials	Expanded perlite	Perlite, expanded
Aggregates sand stones	Aggregates	Expanded materials	Expanded shale aggregates	Shale, expanded, aggregates
Aggregates sand stones	Aggregates	Expanded materials	Expanded vermiculite	Vermiculite, expanded
Aggregates sand stones	Aggregates	Plastic aggregate	Average plastics beads	Plastic, beads
Aggregates sand stones	Aggregates	Plastic aggregate	EPS beads aggregates	EPS, plastic, beads, aggregates
Aggregates sand stones	Aggregates	Plastic aggregate	PE beads	PE, plastic, beads, aggregates
Aggregates sand stones	Aggregates	Plastic aggregate	PP beads	PP, plastic, beads, aggregates
Aggregates sand stones	Aggregates	Plastic aggregate	PS beads	PS, plastic, beads, aggregates
Aggregates sand stones	Aggregates	Plastic aggregate	PS-PVC beads	PS-PVC, plastic, beads, aggregates
Aggregates sand stones	Aggregates	Plastic aggregate	PVC beads	PVC, plastic, beads, aggregates
Aggregates sand stones	Aggregates	Plastic aggregate	Recycled plastic aggregates	Plastic, recycled, aggregates
Aggregates sand stones	Aggregates	Recycled aggregate	Fly ash stabilised	Soil, stabilized, fly ash
Aggregates sand stones	Aggregates	Recycled aggregate	Slag GGBS	Slag, GGBS, Ground Granulated Blast Furnace
Aggregates sand stones	Gravel sand soil	General values	Aggregates and sand general	Aggregates and sand general
Aggregates sand stones	Gravel sand soil	General values	Rammed soil general	Soil general
Aggregates sand stones	Gravel sand soil	General values	Sand general	Sand general
Aggregates sand stones	Gravel sand soil	Gravel	Gravel dry 1.3 to 5.1cm	Gravel dry 1.3 to 5.1cm
Aggregates sand stones	Gravel sand soil	Gravel	Gravel dry loose	Gravel dry loose
Aggregates sand stones	Gravel sand soil	Gravel	Gravel soil	Gravel soil
Aggregates sand stones	Gravel sand soil	Gravel	Gravel with sand natural	Gravel with sand natural
Aggregates sand stones	Gravel sand soil	Gravel sand soil genera	Sand and gravel general	Sand and gravel general
Aggregates sand stones	Gravel sand soil	Sand	Sand rammed	Sand rammed
Aggregates sand stones	Gravel sand soil	Sand	Sand with gravel	Sand with gravel
Aggregates sand stones	Gravel sand soil	Soil	Cement stabilised	Cement stabilised
Aggregates sand stones	Gravel sand soil	Soil	Earth dry	Earth dry
Aggregates sand stones	Gravel sand soil	Soil	GGBS stabilised	GGBS stabilised
Aggregates sand stones	Gravel sand soil	Soil	Silt	Silt
Aggregates sand stones	Stones	Stone hard	Basalt	Stone basalt
Aggregates sand stones	Stones	Stone hard	Gneiss	Stone Gneiss
Aggregates sand stones	Stones	Stone hard	Granite	Stone Granite
Aggregates sand stones	Stones	Stone hard	Stone hard unspecified	Stone hard general
Aggregates sand stones	Stones	Stone softer	Dolomite	Dolomite
Aggregates sand stones	Stones	Stone softer	Gypsum solid	Gypsum solid
Aggregates sand stones	Stones	Stone softer	Limestone solid	Stone solid
Aggregates sand stones	Stones	Stone softer	Marble	Stone Marble
Aggregates sand stones	Stones	Stone softer	Sandstone	Stone sandstone
Aggregates sand stones	Stones	Stone softer	Tufa	Stone tufa
Aggregates sand stones	Stones	Stones general	Rubble stone	Rubble stone
Aggregates sand stones	Stones	Stones general	Stone general	Stone general
Blocks bricks and precast products	Hollow block	Concrete	Lightweight concrete hollowblock	Hollowblock lightweight concrete
Blocks bricks and precast products	Hollow block	Concrete	Medium weight	Hollowblock medium weight concrete
Blocks bricks and precast products	Hollow block	Concrete	Normal weight	Hollowblock normal weight concrete
Blocks bricks and precast products	Hollow block	Clay concrete LECA ho	LECA hollow block general	
Blocks bricks and precast products	Hollow block	Clay concrete LECA ho	LECA hollow block fine concrete	
Blocks bricks and precast products	Hollow brick	Clay	Engineering brick	Engineering brick
Blocks bricks and precast products	Hollow brick	Clay	Hollow clay brick	Hollow clay brick
Blocks bricks and precast products	Other precast product	Beams columns	Beams columns concrete	Precast concrete beams and columns
Blocks bricks and precast products	Other precast product	Hollowcore floor slab	Hollowcore floor slab 150mm	Hollowcore floor slab 150mm
Blocks bricks and precast products	Other precast product	Hollowcore floor slab	Hollowcore floor slab 200mm	Hollowcore floor slab 200mm
Blocks bricks and precast products	Other precast product	Hollowcore floor slab	Hollowcore floor slab 250mm	Hollowcore floor slab 250mm
Blocks bricks and precast products	Other precast product	Hollowcore floor slab	Hollowcore floor slab 300mm	Hollowcore floor slab 300mm
Blocks bricks and precast products	Other precast product	Hollowcore floor slab	Hollowcore floor slab 400mm	Hollowcore floor slab 400mm
Blocks bricks and precast products	Other precast product	Hollowcore floor slab	Hollowcore floor slab average	Hollowcore floor slab average

Figure 53 Overview of the tab "**Search Material**" in BUD-MI

The "Search Material" tab is designed to help users efficiently locate specific materials within the BUD-MI material classification system. This classification forms the foundation of the BoM tab, where materials must be labeled with the correct terminology. The "Search Material" tab becomes especially useful in ensuring accurate material identification. The BUD-MI material classification system (L1-L4) is the backbone of the BoM tab. As such, getting the right terminology is key, which is what the "Search Material" tab can help with. The tab helps users quickly locate specific materials within the BUD-MI material classification system.

The tab is organized into five columns: the first four correspond to BUD-MI's L1-L4 categories, while the fifth provides a description of the L4 material and lists any synonyms. This feature helps

users navigate terminology differences across languages, fields, and industries. Ultimately, this tab ensures quick access to the needed materials, streamlining the data retrieval process.

It is organized in five columns. The first four correspond to the BUD-MI material classification system (L1-L4). The fifth column provides a description and synonyms of each material, which alleviates terminology differences across languages, fields, or industries.

To find a material, users can use three ways:

- the “find” Excel function (shortcut [Ctrl+ F] or [Cmd + F])
- the filter function at the top of each column
- the filter function at the top of the keyword column, in which synonyms and related materials are listed to help easily find the relevant material in the list.

An example can be seen in Figure 54 below.

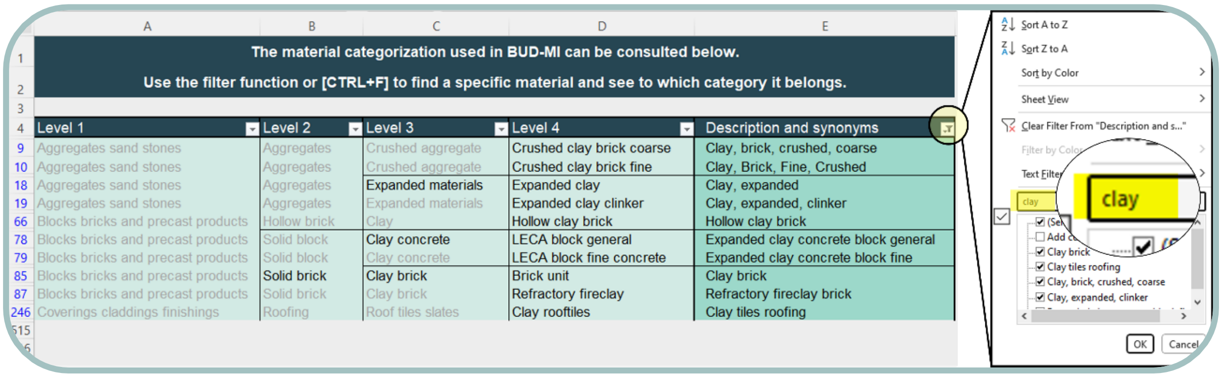

Figure 54 Searching for materials containing “clay” using the Filter function in the last column (Description and Synonyms), in the tab “Search material” of BUD-MI.

Chapter III.

Understanding & Generating Results

After all data has been inputted into BUD-MI, results are automatically generated in various formats, some of which can be tailored by the user. These result formats are described below.

1. Results - Summary

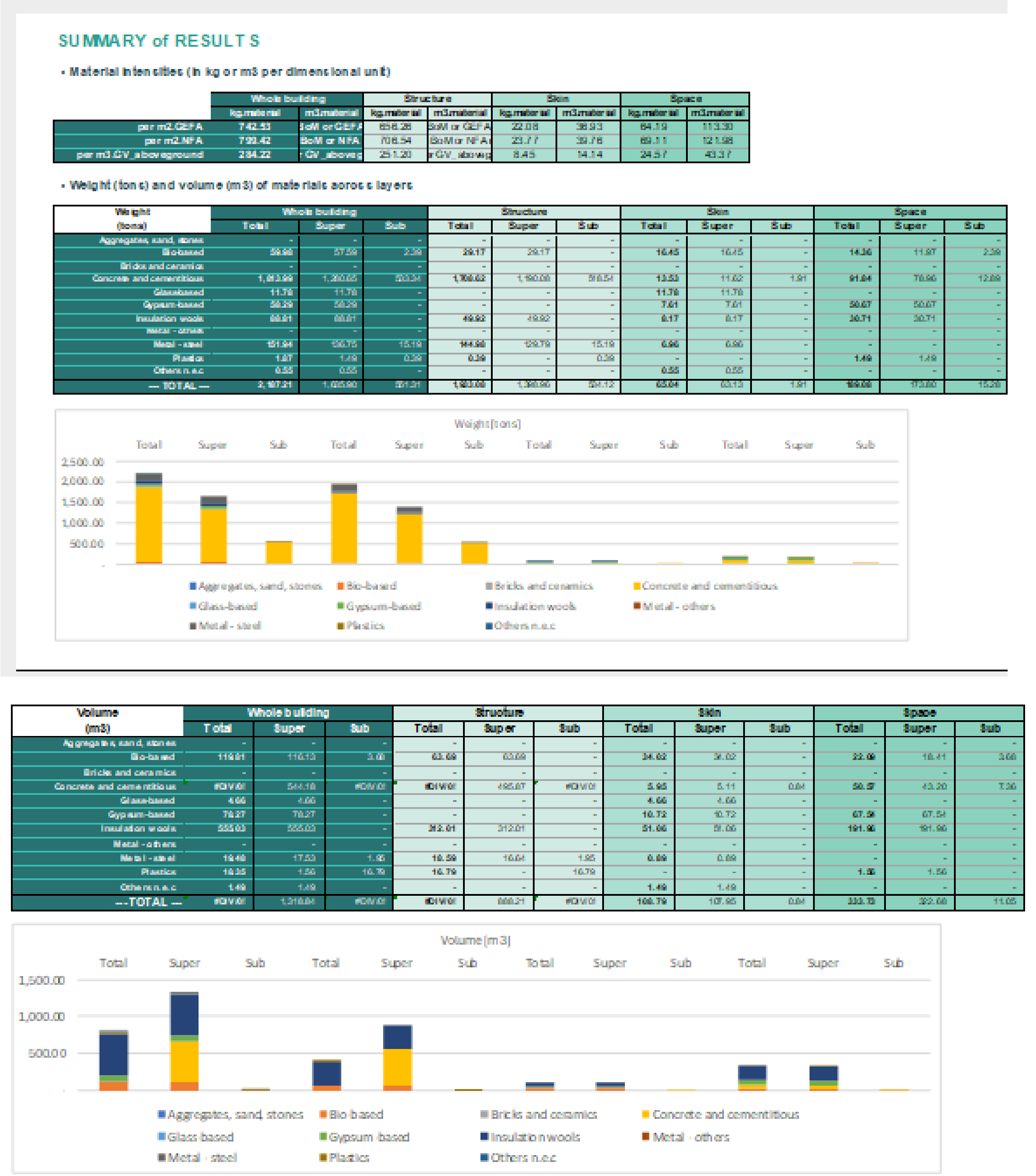

Figure 55 Overview of the tab "**Result – summary**” in BUD-MI.

In the tab “Results – summary”, users can get an overview of results. The core purpose of this tab is to give the possibility to users to sense check their results and spot potential mistakes made during data collection. The tab is organized in two sections.

- In “Material intensities”, users see the aggregated material intensity of the building and its layers, in various units.

	Whole building		Structure		Skin		Space	
	kg	m3	kg	m3	kg	m3	kg	m3
per m2.GEFA	1335	735	821	417	425	262	89	56
per m2.NFA	1444	795	888	451	460	283	97	61
per m3.GV_aboveground	307	169	189	96	98	60	21	13

Figure 56 Summary of result in BUD-MI: total material intensity in various measurement and reference units for the whole building and across building shearing layers.

- In “Weight and volume of materials across layers”, results are disaggregated across general material categories, building shearing layers, and super/sub. Results are displayed as tables and graphs, both in weight and volume.

• Weight (tons) and volume (m3) of materials across layers

Weight (tons)	Whole building			Structure			Skin			Space		
	Total	Super	Sub	Total	Super	Sub	Total	Super	Sub	Total	Super	Sub
Aggregates, sand, stones	-	-	-	-	-	-	-	-	-	-	-	-
Bio-based	3.48	3.48	-	-	-	-	-	-	-	3.48	3.48	-
Bricks and ceramics	157.43	157.43	-	157.43	157.43	-	-	-	-	-	-	-
Concrete and cementitious	#VALUE!	7.92	1,009.73	1,009.73	-	1,009.73	7.92	7.92	-	-	-	-
Glass-based	4.54	4.54	-	-	-	-	-	-	-	4.54	4.54	-
Gypsum-based	4.90	4.90	-	-	-	-	-	-	-	4.90	4.90	-
Insulation wools	0.34	0.34	-	-	-	-	0.34	0.34	-	-	-	-
Metal - others	10.51	10.51	-	-	-	-	10.51	10.51	-	-	-	-
Metal - steel	189.33	146.61	42.72	189.33	146.61	42.72	-	-	-	-	-	-
Plastics	1.05	0.74	0.31	-	-	-	0.31	-	0.31	0.74	0.74	-
Others n.e.c	-	-	-	-	-	-	-	-	-	-	-	-
--- TOTAL ---	#VALUE!	336.46	1,052.75	1,356.49	304.04	1,052.45	19.08	18.77	0.31	13.65	13.65	-

Figure 57 Table summarizing results in BUD-MI: total weight of construction materials in the whole building, across building shearing layers, and super/sub.

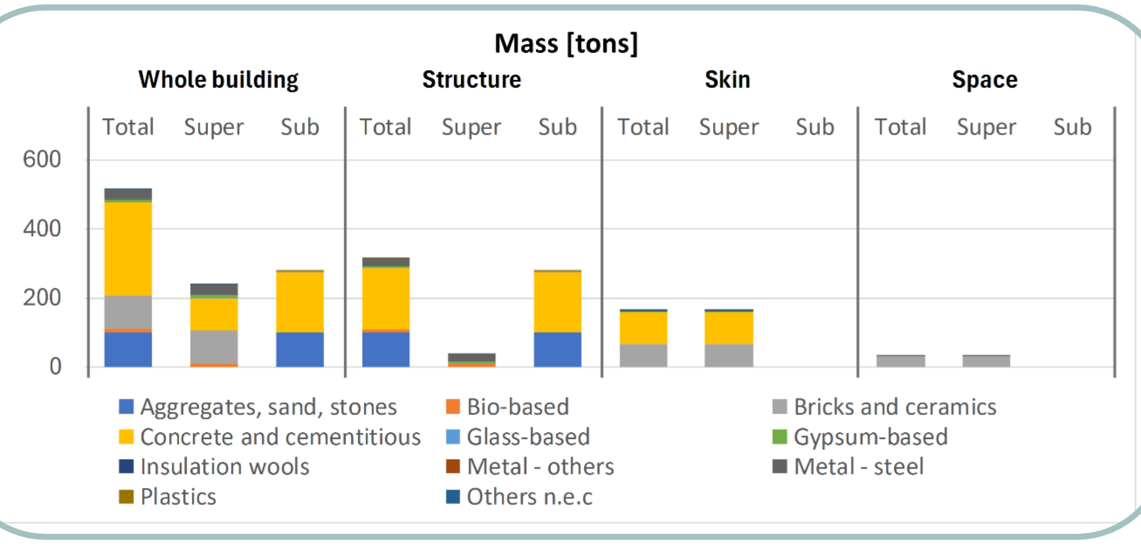

Figure 58 Bar chart summarizing result in BUD-MI: total weight of construction materials in the whole building, across building shearing layers, and super/sub.

2. Bespoke MI Format - Settings & Results

The tabs *"Bespoke MI Format - Settings"* and *"Bespoke MI Format - Results"* work together. In *Settings*, users tailor the MI format, which is then displayed accordingly in the *Results* tab.

2.1. Bespoke MI format - settings

BESPOKE FORMATTING OF RESULTS

In the table below, select the various formatting options for MI result.

Click here to see the results according to your selected options (tab "Results_bespoke").

**Select material categorisation**	
**Select unit of measurement**	**m2.GFA**
**Select quantification unit**	**kg**
**Select shearing layers**	*Enter 1 next to the layer(s) to include in the results*
**Structure**	1
**Space**	1
**Skin**	1
**Select building elements**	*Enter 1 next to the element(s) to include in the results*
**Super**	
**Beams**	1
**Columns**	1
**Ceiling**	1
**External walls**	1
**Internal walls**	1
**Roof**	1
**Stairs and ramps**	1
**Upper floors**	1
**Windows and external doors**	1
**Sub**	
**Lowest floor**	1
**Basement and retaining walls**	1
**Foundations**	1

Figure 59 Overview of the tab "**Bespoke MI format - settings**" in BUD-MI.

In this tab, select the various options relevant to the formatting required for MI results. Five key formatting choices must be made: material classification, building size unit, quantification unit, as well as which shearing layers to include, and which building elements.

2.1.1. Material classification

Select the material classification best fitting your purpose. The material classifications readily available in BUD-MI are presented in Table 17 below, together with their number of tiers, purpose, and geographical relevance. Detailed classifications can be found in Annex.

Additional classifications can be added, following the explanations in Chapter V – Section 2.

Table 17 Material classifications compiled from existing research and grey literature to answer requirements of "project-bespoke MI," "industry-relevant results," and "cumulative research." Each classification serves a specific purpose.

Material classifications	Tiers	Purpose	Geographic relevance	Source
BUD-MI (input)	3	Facilitate material selection during data collection.	Global	*This article*
BUD-MI (result summary)	1	Summarize results for quick assessment.	Global	*This article*
Open MI database	4	Share and compare MI data.	Global	(Heeren and Fishman 2019),
Economy-wide MFA	3	Enable the use of MIs with existing MFA frameworks.	Global	(Eurostat 2013)
Global MFA	2		Global	(International Resource Panel, 2024)
Industry-oriented	3	Facilitate sharing of results with construction actors.	Sweden/Europe	(BK04 Vilma, 2023)
European List of Waste (European Waste Codes)	2	Support waste management considerations.	Europe	(Eurostat 2010)

2.1.2. Building size unit

The building size unit should be chosen in accordance with the building inventory with which the MI is to be used.

Currently, BUD-MI proposes three building size units – but more can easily be added. The three units are:

- **m2.GEFA.** "Gross external floor area" (GEFA) is also called Total Floor Area. It is the floor space covered and enclosed to full height for all levels – including attics, basements, etc. Any area not fully enclosed (e.g., balcony, terrasse) is excluded.
- **m2.NFA.** "Net Floor Area" (NFA) is the area between enclosing elements. It is calculated by subtracting the construction area (taken up by walls, columns, partitions) from the GEFA. (ISO 9836:2017)
- **m3.GV_aboveground**. Gross Volume of building aboveground, including roof volume. Building parts underground – such as basements, foundations, layers of hardcore, and the like – are not included. (Adapted from ISO 9836:2017)

2.1.3. Material quantification unit

The material quantification unit should be chosen in accordance with the aim of the study. Currently, BUD-MI proposes two quantification units: kilograms (kg) for mass of materials, and

cubic meters (m^3) for volume of materials. Volume refers to bulk volume (also called apparent volume).

2.1.4. Shearing layers

Select which shearing layer to include. Definition of each shearing layer used in BUD-MI (Structure, skin, space) can be found in Section 3.1.4 of this Chapter.

2.1.5. Building elements:

Select which building element to include. In the tab, building elements are organized according to whether they are in the "Super" of "Sub" part of the building. (See Figure 59) Definition of each "Super" and "Sub" can be found in Section 3.1.2. of this Chapter. Definitions of each building element can be found in Section 3.1.3 of this Chapter.

2.2. Bespoke MI format – results

Building_layer	Element	Vertical_scope	Near_component	Unit	#REF!
Structure	Beams	Super	Structure_Beams_Super	kg/m2.GFA	#REF!
Structure	Columns	Super	Structure_Columns_Super	kg/m2.GFA	
Structure	Ceiling	Super	Structure_Ceiling_Super	kg/m2.GFA	
Structure	External walls	Super	Structure_External walls_Super	kg/m2.GFA	
Structure	Internal walls	Super	Structure_Internal walls_Super	kg/m2.GFA	
Structure	Roof	Super	Structure_Roof_Super	kg/m2.GFA	
Structure	Stairs and ramps	Super	Structure_Stairs and ramps_Super	kg/m2.GFA	
Structure	Upper floors	Super	Structure_Upper floors_Super	kg/m2.GFA	
Structure	Windows and external doors	Super	Structure_Windows and external doors_Super	kg/m2.GFA	
Structure	Lowest floor	Sub	Structure_Lowest floor_Sub	kg/m2.GFA	
Structure	Basement and retaining wal	Sub	Structure_Basement and retaining walls_Sub	kg/m2.GFA	
Structure	Foundations	Sub	Structure_Foundations_Sub	kg/m2.GFA	
Space	Beams	Super	Space_Beams_Super	kg/m2.GFA	
Space	Columns	Super	Space_Columns_Super	kg/m2.GFA	
Space	Ceiling	Super	Space_Ceiling_Super	kg/m2.GFA	
Space	External walls	Super	Space_External walls_Super	kg/m2.GFA	
Space	Internal walls	Super	Space_Internal walls_Super	kg/m2.GFA	
Space	Roof	Super	Space_Roof_Super	kg/m2.GFA	
Space	Stairs and ramps	Super	Space_Stairs and ramps_Super	kg/m2.GFA	
Space	Upper floors	Super	Space_Upper floors_Super	kg/m2.GFA	
Space	Windows and external doors	Super	Space_Windows and external doors_Super	kg/m2.GFA	
Space	Lowest floor	Sub	Space_Lowest floor_Sub	kg/m2.GFA	
Space	Basement and retaining wal	Sub	Space_Basement and retaining walls_Sub	kg/m2.GFA	
Space	Foundations	Sub	Space_Foundations_Sub	kg/m2.GFA	
Skin	Beams	Super	Skin_Beams_Super	kg/m2.GFA	
Skin	Columns	Super	Skin_Columns_Super	kg/m2.GFA	
Skin	Ceiling	Super	Skin_Ceiling_Super	kg/m2.GFA	
Skin	External walls	Super	Skin_External walls_Super	kg/m2.GFA	
Skin	Internal walls	Super	Skin_Internal walls_Super	kg/m2.GFA	
Skin	Roof	Super	Skin_Roof_Super	kg/m2.GFA	
Skin	Stairs and ramps	Super	Skin_Stairs and ramps_Super	kg/m2.GFA	
Skin	Upper floors	Super	Skin_Upper floors_Super	kg/m2.GFA	
Skin	Windows and external doors	Super	Skin_Windows and external doors_Super	kg/m2.GFA	
Skin	Lowest floor	Sub	Skin_Lowest floor_Sub	kg/m2.GFA	
Skin	Basement and retaining wal	Sub	Skin_Basement and retaining walls_Sub	kg/m2.GFA	
Skin	Foundations	Sub	Skin_Foundations_Sub	kg/m2.GFA	

Drag formula
Drag formula across the table

Figure 60 Overview of the tab "**Bespoke MI format - results**" in BUD-MI. Note that the formula in F2 must be dragged across the table.

3. Results - Open MI DB

sid	
Country	Sweden
Region	NA
construction_period_start	NA
construction_period_end	1990
steel	12,8
copper	0
aluminum	0
unspecified_metal	0
wood	51,11
paper_cardboard	1,23
straw	0
concrete	360,92
cement	0
aggregates	0
brick	0
mortar_plaster	0
mineral_fill	0
plaster_board_gypsum	35,04
Adobe	0
Asphalt	0
Bitumen	0
natural_stone	0
cement_asbestos	0

Figure 61 Excerpt of results in the tab "**Results – Open MI DB**" in BUD-MI.

MI results can be generated in the format used in the open MI database developed by (Heeren & Fishman, 2019), as shown in Figure 61. These results can be exported in CSV and imported into the database. We refer the user to the scientific article of (Heeren & Fishman, 2019) and associated material (including GitHub) for more information.

Chapter IV.

SUPPORTING DATA

Both quantitative and qualitative data support the functioning of BUD-MI. Quantitative data includes construction material density. Different sets of qualitative data are used to support main functions such as dropdown lists and predefined sets of options.

1. QUANTITATIVE DATA

1.1. Densities

The density dataset includes the density of 404 construction materials (from 267 references), including linear and areal densities where relevant and available. A three-tier material categorization to help users find materials quickly. The categorization is a hybrid of function-based (used by architects) and material-based (used by industrial ecologists) classifications. For example, "timber boards" are found in both "timber product" and "coverings" categories.

2. QUALITATIVE DATA

Qualitative data in BUD-MI supports functionalities such as lookups, filtering, and dropdowns. Most of this data is static (it does not change frequently) and essential for BUD-MI to function correctly. Still, all reference data can be updated or changed manually (see Chapter V).

2.1. BUD-MI core material classification

BUD-MI core material classification can be found in Annex 1.1 of this document. It can also be found in BUD-MI's tab "Search materials" (tab 7) and "BUD-MI material classification" (tab 13).

2.2. Crossmatch material classifications

Several material classifications are part of BUD-MI, crossmatched with BUD-MI's core material classification. These material classifications are displayed in Annex (1.2 – 1-7).

Within BUD-MI, the crossmatch material classification can be found in Tab 15.

2.3. Dropdowns

All dropdowns used in BUD-MI can be found in tab 12. The core ones are as follows:

- Status: In progress, Finished, Double-checked
- Data collection method: BIM data, Digital off-site survey, Digital on-site survey, Drawings (e.g. plans, cross-sections), Manual on-site survey - destructive, Manual on-site survey - non-destructive
- Context: Urban, Suburban, Rural, Unknown
- Foundation type: Shallow, Deep, Unknown
- Shallow: Individual/isolated footing, Combined footing, Spread/strip/wall footings, Raft or Mat Foundations
- Deep: Pile foundations, Drilled Shafts or Caisson Foundation, Unknown
- World region: Africa, Northern; Africa, Southern; America, North; America, South; America, Central; America, Caribbean; Asia-Pacific: Central & South Asia; Asia-Pacific: Northeastern Asia; Asia-Pacific: Southeastern Asia; Asia-Pacific: Australia and Oceania; Europe, Northern; Europe, Southern; Europe, Eastern; Europe, Western.
- Shape (complexity): Circular/elliptical/similar, Square/rectangular/similar, Complex
- Design: Simple, Bespoke, Complex
- Number of stories: 0–3, 4–7, 8–20, 21–30, 31–50, Over 50

- Structural (predominant): Timber, Concrete (cast in-situ), Concrete (prefab), Steel frame (pre-cast floor), Steel frame (in-situ floor), Load-bearing masonry
- Roof type: Butterfly, Dormer, Flat, Gable, Gambrel, Hip, Mansard, M-shaped, Pyramid, Shed, Other (please specify)
- Roof material: Asbestos cement (AC sheet), Built-up roofing (tar-and-gravel), Clay tiles, Concrete tiles, Fibercement, Fibercement sheets (fibro), Membrane roofing, Metal, Plastic, Slate, Thatch, Unknown, Other (please specify)
- External wall (predominant): Block masonry, Brick masonry, Concrete, Fibercement, Glass/curtain walling, Metal, Plastic, Render/plastering/stucco, Stone/rubble masonry, Wooden, Other (please specify)
- Nature of work: New build, Renovation, Extension, Major adaptation, Demolition
- Building use: Residential - Detached house, Residential - Semi-detached house, Residential - Linked house, Residential - Apartment building, Office, Commercial, Shopping centre, Industrial, Hotel, Car park, Warehouse, Educational, Hospital, Airport terminal, Railway station, Ferry terminal, Plant facility, Other
- Building shearing layer: Structure, Skin, Space
- Super: Beams, Columns, Ceiling, External walls, Roof
- Sub: Foundations, Basement and retaining walls, Lowest floor, Internal walls.

Chapter V.

Modifying & Complementing BUD-MI

1. Adding Materials to BUD-MI

It is likely that uncommon construction materials not listed in BUD-MI will be encountered during data collection. This section describes how to add these construction materials and their specifications (density and material category) to BUD-MI. The example of "coke ash" (previously used in Sweden for insulation purposes) is used to illustrate the procedures described.

There are two ways to add a material and its specifications to BUD-MI, depending on how often the material may be encountered during data collection:

- If the material is expected to be encountered multiple times during data collection, adding its information to the "background data" is beneficial (section 1.1 of this chapter).
- If the material is a sporadic occurrence, users can add data "on-the-fly" (section 1.2 of this chapter).

1.1. Adding materials to background data

A large part of BUD-MI dropdown functions through the Name Manager (see Tipbox 2 below).

Name Manager

In Excel, the Name Manager is essential for managing complex workbooks, by giving names to cells, ranges, formulas, or constants.

The Name Manager also ensures nested dropdowns are correctly set up, dynamically managed, and easy to maintain.

To access the Name Manager: and create, edit, delete, and view all the named ranges in a workbook:

- Keyboard shortcut: CTRL+F3 (if your keyboard has an 'function lock key', aka Fn key: CTRL+F3+Fn)
- Manually: Go to the Formulas tab in the Excel ribbon > Click on Name Manager in the Defined Names group.

Tipbox 2 Name manager in Excel

In the following subsections, "coke ash" is used as an example material to add to BUD-MI.

1.1.1. Tab "Crossmatch material classification"

Effect of this step: the material is accounted for when generating results.

1/ Insert a new row (where specified by the template). This ensures the data will be included in the name ranges.

2/ Write the name of the material in the column 'BUD MI L4'.

	BUD MI L1	BUD MI L2	BUD MI L3	BUD MI L4
483	Windows and doors	Door external	No glazing	Stainless steel ext door no glazing
484	Windows and doors	Window	Timber frame	Timber frame
485	Windows and doors	Window	Glazing	Triple glazing
486	Windows and doors	Door external	Glazing door	Triple glazing
487	Windows and doors	Window	Aluminum frame	Window aluminum frame
488	Windows and doors	Door external	No glazing	Wood door ext
489	Windows and doors	Door internal	Wood door	Wood door int
490				Coke ash
491				
492	TO ADD A MATERIAL, INSERT A ROW ABOVE THIS ONE AND FILL ALL RELEVANT INFORMATION			

Figure 62 Add “coke ash” to the material list, under BUD MI L4.

3/ Assign it to its relevant parent categories (BUD MI L1 – BUD MI L3). To do this, consult the material classification used in BUD-MI in **Annex 1.1** of this user guide (or in the tab “Search materials” in BUD-MI).

	BUD MI L1	BUD MI L2	BUD MI L3	BUD MI L4
483	Windows and doors	Door external	No glazing	Stainless steel ext door no glazing
484	Windows and doors	Window	Timber frame	Timber frame
485	Windows and doors	Window	Glazing	Triple glazing
486	Windows and doors	Door external	Glazing door	Triple glazing
487	Windows and doors	Window	Aluminum frame	Window aluminum frame
488	Windows and doors	Door external	No glazing	Wood door ext
489	Windows and doors	Door internal	Wood door	Wood door int
490	Insulation	Loose fill granulates	Mineral loose granulates	Coke ash
491				
492	TO ADD A MATERIAL, INSERT A ROW ABOVE THIS ONE AND FILL ALL RELEVANT INFORMATION			

Figure 63 Coke ash is used for “insulation” (BUD MI L1). It is “Loose fill granulates” (BUD MI L2), more precisely to “Mineral loose fill granulates” (BUD MI L3).

4/ Make sure it will be accounted in your summary results by matching it to the relevant material in the column “Result_summary_matclassif”. To do this, consult the material classification used for results summary in **Annex 1.2**.

	BUD MI L1	BUD MI L2	BUD MI L3	BUD MI L4	Result_summary_matclassif
483	Windows and doors	Door external	No glazing	Stainless steel ext door no glazing	Metal - steel
484	Windows and doors	Window	Timber frame	Timber frame	Bio-based
485	Windows and doors	Window	Glazing	Triple glazing	Glass-based
486	Windows and doors	Door external	Glazing door	Triple glazing	Glass-based
487	Windows and doors	Window	Aluminum frame	Window aluminum frame	Metal - others
488	Windows and doors	Door external	No glazing	Wood door ext	Bio-based
489	Windows and doors	Door internal	Wood door	Wood door int	Bio-based
490	Insulation	Loose fill granulates	Mineral loose granulates	Coke ash	Others n.e.c
491					
492	TO ADD A MATERIAL, INSERT A ROW ABOVE THIS ONE AND FILL ALL RELEVANT INFORMATION				

Figure 64 “Coke ash” belongs to the category “Others n.e.c.” in the material classification used to summarize results in BUD-MI (Result_summary_matclassif).

5/ Optional: You may also match it across all material classifications that are of interest to your work, using the material classifications supplied in Annex 1. See also: embed your own material classification in BUD-MI (Chapter V, Section 2).

	BUD MI L1	BUD MI L2	BUD MI L3	BUD MI L4	ewMFA_code level_2-3
482	Windows and doors	Door external	Glazing door	Single glazing	MF.3.compound
483	Windows and doors	Door external	No glazing	Stainless steel ext door no glazing	MF.2.Fe
484	Windows and doors	Window	Timber frame	Timber frame	MF.1.3.1
485	Windows and doors	Window	Glazing	Triple glazing	MF.3.compound
486	Windows and doors	Door external	Glazing door	Triple glazing	MF.3.compound
487	Windows and doors	Window	Aluminum frame	Window aluminum frame	MF.2.Al
488	Windows and doors	Door external	No glazing	Wood door ext	MF.1.3.1
489	Windows and doors	Door internal	Wood door	Wood door int	MF.1.3.1
490	Insulation	Loose fill granulates	Mineral loose granulates	Coke ash	MF.4.compound
491					
492	TO ADD A MATERIAL, INSERT A ROW ABOVE THIS ONE AND FILL ALL RELEVANT INFORMATION				

Figure 65 "Coke ash" would be classified as "MF.4.compound" in economy-wide MFA.

1.1.2. Tab “**Densities**”

Effect of this step: the density of the material density is automatically returned when you select it in the BoM.

1/ Insert a new row (where specified by the template) and enter the name of the material. Make sure you use the same material name as in the previous step (1.1.1). You can also give it a brief description.

	A	B
1		
2		
3	**Material (BUD MI L4)**	**Material_description**
403	Wool	Carpet wool
404	Wool felt	Felt underlay wool
405	XPS board	XPS board
406	Zinc general	Zinc general
407	Coke ash	Coke ash (insulation material used in older Swedish buildings)
408		
409		
410		
411	*TO ADD A MATERIAL AND ITS DENSITY INFORMATION, INSERT A ROW ABOVE THIS ONE*	

Figure 66 Add the material in the first column, using the same name as in the tab “Crossmatch material classification”.

2/ Enter the density of the material in the relevant column(s) (i.e., linear density and/or areal density and/or volumic density). The reference and any comment can also be entered.

	A	C	D	E	F	G
1		**Apparent densities**				
2						
3	**Material (BUD MI L4)**	**Linear (kg/m)**	**Areal (kg/m2)**	**Volumic (kg/m3)**	**References**	**Comments**
403	Wool	Express dimension in m3	Express dimension in m3	318	Ref. 35	
404	Wool felt	Express dimension in m3	Express dimension in m3	160	Ref. 67	
405	XPS board	Express dimension in m3	Express dimension in m3	34	Refs. 86; 87; 88	
406	Zinc general	Express dimension in m3	Express dimension in m3	7100	Ref. 275	
407	Coke ash	Express dimension in m3	Express dimension in m3	700	Svensk Bygg Norm (1967)	
408						
409						
410						
411	*TO ADD A MATERIAL AE AND FILL IT WITH THE RELEVANT INFORMATION*					

Figure 67 The volumic density of coke ash is 700 kg/m3, as stated in the “Svensk Bygg Norm” (Swedish Building Regulation) from 1967.

3/ Optional: enter the uncertainty (standard deviation, STDEV) associated with the density. The coefficient of variation (CV) is calculated as $CV = STDEV \ / \ MEAN$, where the mean is the density entered in step 2/. If the density is unknown, the default CV (=cell P2) can be used.

	A	N	O	P	Q	R	S	
1		*an be changed in cells I2, L2, and P2 (orange boxes).*						
2		**Volumic densities uncertainties**		20%				
3	**Material (BUD MI L4)**	**MIN (kg/m3)**	**MAX (kg/m3)**	**STDEV (kg/m3)**	**CV (%)**	**Number of data points**	**References**	**Comments on STDEV**
403	Wool			78	25%	9	Ref. 35	
404	Wool felt			*32*	*20%*			CV ASSUMED - SEE CELL P2
405	XPS board			5.93	18%	10	Refs. 86; 87; 88	
406	Zinc general			0	0%		Ref. 275	Density of metallic elements is a
407	Coke ash			*140*	*20%*			CV ASSUMED - SEE CELL P2
408								
409								
410								
411	*TO ADD A MATERIAL A*							

Figure 68 Enter the uncertainty and coefficient of variation of the density.

1.1.3. Tab “Material_classif_dropdowns”

Effect of this step: the material appears in the dropdowns for material selection in the BoM.

1/ Add the material at the end of the list in the relevant Level_3 category (which you tracked in Step 1.1.1.)

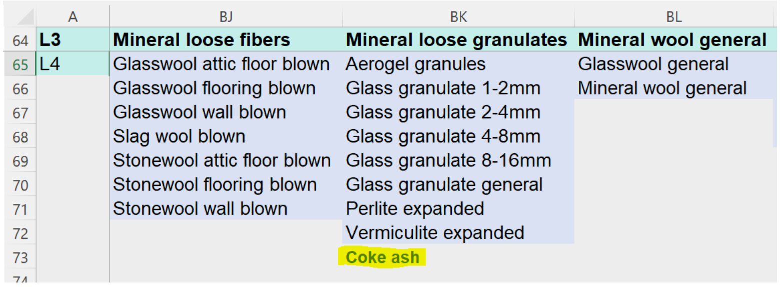

Figure 69 “Coke ash” is written at the end of the list of materials in the “Mineral loose granulates” (BUD MI L3)

2/ Include the material within the cell range so it is included in the name manager (See Tipbox 2). This can be done in two ways:

“Inserting into the named range”

- Insert it into the list by holding the SHIFT key.
- 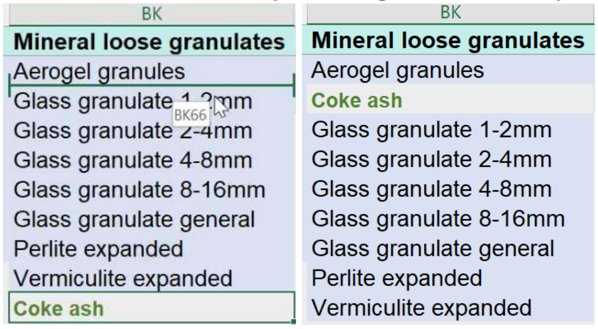

“Extending the named range”

- Open the name manager and find the range’s name (same name as the material category).
- Edit the range to include the additional material.

3/ At this stage, make sure the modification of the named range worked. To do this, simply select your range, and verify that the right name appears in the Name Box.

1.2. On-the-fly

Most “on-the-fly” data additions are performed within the tab “Bill of Material”. However, for the material to be accounted for when generating results, it must be matched to its relevant material categories in tab “Crossmatch material classification".

1.2.1. Tab “Bill of materials”

1/ In **level 4 of material categories**, enter the name of the material (override the cell). Level 1-3 categories can remain empty.

2/ Enter the density (override cell in relevant column).

1.2.2. Tab "Crossmatch material classification"

Follow the steps described in Section 1.1.1 of this chapter.

2. ADDING A MATERIAL CLASSIFICATION

BUD-MI includes several material classifications, each with a different purpose. If needed, the user can add a material classification by following the steps outlined below.

- In Tab "Crossmatch material classific.", add a new column, and name the material classification (e.g., *newclassification2025*)
- Cross-match each material of the new classification to "BUD-MI level 4"
- In the tab "Dropdowns", locate the list of material categories used in the template (cells E51:E66).
- Add the name of the new classification to the list. It should be the exact same name as the one given in the first step, i.e., *newclassification2025*.
- In the Name Manager, make sure that "*newclassification2025*" is part of "Mat_cats". For more information on Excel's Name Manager, consult Tipbox 2 in Section 1.1 of this chapter.

REFERENCES

Brand, S. (1994). *How Buildings Learn: What Happens After They're Built*. Viking Press.

BREEAM. (2016). *How does BREEAM define total useful floor area*. https://kb.breeam.com/knowledgebase/nc14-ene-02-how-does-breeam-define-total-useful-floor-area/

Cunningham, T. (2015). *Measuring Building Perimeters and Centrelines - Worked Examples.* https://doi.org/10.21427/b3mf-pw56

Eurostat. (2010). *Guidance on classification of waste according to EWC-Stat categories. Supplement to the Manual for the Implementation of the Regulation (EC) No 2150/20023 on Waste Statistics.*

Eurostat. (2018). Economy-wide material flow accounts - Handbook. In *Manuals and guidelines*. https://doi.org/10.2785/158567

Heeren, N., & Fishman, T. (2019). A database seed for a community-driven material intensity research platform. *Scientific Data*, *6*(1), 23. https://doi.org/10.1038/s41597-019-0021-x

Ibrahim, M. R., Haworth, J., & Cheng, T. (2020). Understanding cities with machine eyes: A review of deep computer vision in urban analytics. *Cities*, *96*(August 2019), 102481. https://doi.org/10.1016/j.cities.2019.102481

ICMS Coalition. (2021). *ICMS: Global Consistency in Presenting Construction Life Cycle Costs and Carbon Emissions*. *November*.

International Resource Panel. (2024). *Technical annex for Global Material Flows Database - 2024 edition*. https://resourcepanel.org/sites/default/files/irp_technical_annex_global_material_flows_database.pdf

IPMSC. (2023). *International Property Measurement Standards: All Buildings*.

ISO 9836:2017. (2017). *Performance standards in building — Definition and calculation of area and space indicators. (ISO Standard No. 9836:2017(E)). International Organization for Standardization.*

Lanau, M., Rosado, L., Tingley, D. D., & Wallbaum, H. (2024). Buildings as material mines. In *Circular Economy for the Built Environment* (pp. 46–68). Routledge. https://doi.org/10.1201/9781003450023-5

Pushkar, S. (2015). Application of life cycle assessment to various building lifetime shearing layers: site, structure, skin, services, space, and stuff. *Journal of Green Building*, *10*(2), 198–214. https://doi.org/10.3992/jgb.10.2.198

RICS. (2021). *New Rules of Measurement 1: Order of cost estimating and cost planning for capital building works* (3rd edition, Issue October). Royal Institution of Chartered Surveyors (RICS). www.rics.org

Schiller, G., Miatto, A., Gruhler, K., Ortlepp, R., Deilmann, C., & Tanikawa, H. (2019). Transferability of Material Composition Indicators for Residential Buildings: A Conceptual Approach Based on a German-Japanese Comparison. *Journal of Industrial Ecology*, *23*(4), 796–807. https://doi.org/10.1111/jiec.12817

Shahi, S., Esfahani, M. E., Bachmann, C., & Haas, C. (2020). A definition framework for building adaptation projects. *Sustainable Cities and Society*, *63*(January). https://doi.org/10.1016/j.scs.2020.102345

UK Government. (2021). *The Building Regulations 2010. Approved document O: Overheating.*

United Nations Statistics Division. (1999). *Standard Country or Area Codes for Statistical Use (M49)*.

Annex

1. BUD-MI MATERIAL CLASSIFICATION

Table A1 Material classification used as the base of BUD-MI, especially in the tab "Bill of Materials".

Level 1	Level 2	Level 3	Level 4
Aggregates sand stones	Aggregates	Aggregates general	Aggregates and sand general
			Coarse aggregate general
		Crushed aggregate	Crushed asphalt compact
			Crushed asphalt loose
			Crushed clay brick coarse
			Crushed clay brick fine
			Crushed concrete
			Crushed dolomite
			Crushed mixed base compact
			Crushed mixed base loose
			Gypsum crushed
			Pumice aggregates
			Stonechips
		Expanded materials	Expanded clay
			Expanded clay clinker
			Expanded glass
			Expanded perlite
			Expanded shale aggregates
			Expanded vermiculite
		Plastic aggregate	Average plastics beads
			EPS beads aggregates
			PE beads
			PP beads
			PS beads
			PS-PVC beads
			PVC beads
			Recycled plastic aggregates
		Recycled aggregate	Fly ash stabilised
			Slag GGBS
	Gravel sand soil	General values	Aggregates and sand general
			Rammed soil general

Level 1	Level 2	Level 3	Level 4
			Sand general
		Gravel	Gravel dry 1.3 to 5.1cm
			Gravel dry loose
			Gravel soil
			Gravel with sand natural
		Gravel sand soil general	Sand and gravel general
		Sand	Sand rammed
			Sand with gravel
		Soil	Cement stabilised
			Earth dry
			GGBS stabilised
			Silt
	Stones	Stone hard	Basalt
			Gneiss
			Granite
			Stone hard unspecified
		Stone softer	Dolomite
			Gypsum solid
			Limestone solid
			Marble
			Sandstone
			Tufa
		Stones general	Rubble stone
			Stone general
Blocks bricks and precast products	Hollow block	Concrete	Lightweight concrete hollowblock
			Medium weight
			Normal weight
		Clay concrete LECA hollow	LECA hollow block general
			LECA hollow block fine concrete
	Hollow brick	Clay	Engineering brick
			Hollow clay brick
	Other precast product	Beams columns	Beams columns concrete
		Hollowcore floor slab	Hollowcore floor slab 150mm
			Hollowcore floor slab 200mm
			Hollowcore floor slab 250mm

Level 1	Level 2	Level 3	Level 4
			Hollowcore floor slab 300mm
			Hollowcore floor slab 400mm
			Hollowcore floor slab average
		Paving	Paving concrete
	Solid block	Aircrete	High strength
			Lightweight aircrete solid block
			Standard aircrete block
		Clay concrete	LECA block general
			LECA block fine concrete
		Concrete aggregate	Medium lightweight dense
			standard dense
			ultra lightweight
		Glass block	Glass block
		Hempcrete block	Hempcrete
	Solid brick	Clay brick	Brick unit
			Double skin
			Refractory fireclay
			Single skin
		Concrete brick	Dense
			Lightweight concrete brick
		Other material	Compressed earth
			Fly ash
			Mud heavy
			Mud low density
			Sand-Lime
	Stone	Rubble	Rubble masonry
Coverings claddings finishings	Ceiling	Plasterboard ceiling	Gypsum fiberboard
			Gypsum hardboard
			Plasterboard
			Plasterboard sheathing
		Plastic ceiling	Fiberglass reinforced plastic
			PVC ceiling board
		Tiles ceiling	Mineral fiber tile ceiling
		Timber board ceiling	Chipboard

Level 1	Level 2	Level 3	Level 4
			CLT
			Fiberboard
			Hardboard HDF
			MDF
			Particle board
			Plywood
			Wood plastic composite board
	External wall finishing	Metal cladding	Aluminum general
			Aluminum sheet profiled 0.7mm
			Aluminum sheet profiled 1mm
			Aluminum sheet profiled general
			Copper general
			Steel sheet profiled 0.5mm
			Steel sheet profiled 0.7mm
			Zinc general
		Mineral cladding	Fibercement board facade cladding
			Fiberglass reinforced plastic
			Stone composite facade board
			Terrazzo tiles
			Thin brick veneer
		Stucco render	Cement lime mortar
			Cement mortar
			Ribbed_steel_lath_plastering
			Stucco 13mm
			Stucco 19mm
			Stucco 25mm
			Stucco general
		Synthetic cladding	GRP sheet profiled
			High-pressure laminate veneer
			Polycarbonate sheet profiled 1mm
			Polycarbonate sheet profiled 2mm
			Polycarbonate sheet profiled general

**Level 1**	**Level 2**	**Level 3**	**Level 4**
			Profiled PVC sheet
		Wall membranes	Breather filter fleece PP 1mm
			Breather HDPE HDPP 0.45mm
			Damp proof course PE 0.46mm
			Vapour control layer PE 0.125mm
			Wall synthetic underlay
		Wooden cladding	CLT
			Hardwood lining
			MDF
			Softwood lining
	Flooring	Flooring carpet	Carpet general
			Carpet PE
			Carpet PET
			Carpet PP
			Nylon 6
			Nylon 6.6
			Simulated wool
			Wool
		Flooring resin	Acrylic
			Epoxy resin
			Liquid vinyl
			Polyurethane
		Flooring synthetic	Linoleum
			PE tiles
			PP tiles
			PVC tiles
			Rubber flooring
			Vinyl flooring
		Flooring tiles ceramics	Ceramics tiles
			Terrazzo tiles
		Flooring wood	CLT
			Laminate
			Parquet
		Subfloor	Cement backer boards
			Chipboard
			Concrete
			Fibergypsum
			Fiberboard
			Gypcrete

Level 1	Level 2	Level 3	Level 4
			Hardboard HDF
			Damp proof membrane LDPE 0.3mm
			MDF
			OSB
			Particle board
			Plywood
		Underlay	Cork board
			Gypsum floorboard
			Jute felt
			Mass-loaded vinyl
			Rubber crumb flooring
			Rubber PUR flooring
			Rubber sponge flooring
			Rubber underlay general
			Wool felt
	Internal walls	Calcium Silicate Sheets	Calcium silicate sheet
		Ceramics tiles	Ceramics tiles
			Ceramics veneer
		Fibercement board	Fibercement board
			Fibercement board indoor use
		Paper based	Paperboard
			Wallpaper
		Plasterboard	Gypsum fiberboard
			Gypsum hardboard
			Plasterboard
			Plasterboard sheathing
			Wet room plasterboard
		Plastic	Fiberglass reinforced plastic
			Vinyl wall covering
		Timber	Chipboard
			CLT
			Fiberboard
			Hardboard HDF
			Hardwood lining
			Laminate
			MDF

Level 1	Level 2	Level 3	Level 4
			OSB
			Particle board
			Plywood
			Softwood lining
			Wood plastic composite board
	Roofing	Roof decking timber boards	CLT
			Fiberboard
			OSB
			Particle board
			Plywood
		Roof membrane	Bituminous roofing membrane
			EPDM rubber roofing membrane
			PVC roofing membrane
			TPO roofing membrane
		Roof sheet flat	Asbestos sheet flat
			Fibercement board
			Fiberglass reinforced plastic
			Polymer modified bitumen sheet flat
			Steel sheet flat 0.5mm
			Steel sheet flat 0.8mm
		Roof sheet profiled	Aluminum sheet profiled 0.7mm
			Aluminum sheet profiled 1mm
			Aluminum sheet profiled general
			Bitumen sheet profiled
			Fibercement sheet profiled
			GRP sheet profiled
			Polycarbonate sheet profiled 1mm
			Polycarbonate sheet profiled 2mm
			Polycarbonate sheet profiled general
			Profiled PVC sheet
			Steel sheet profiled 0.5mm

Level 1	Level 2	Level 3	Level 4
			Steel sheet profiled 0.7mm
		Roof tiles slates	Bituminous shingle
			Ceramics rooftiles
			Clay rooftiles
			Concrete rooftiles
			Fibercement slates
			Slate rooftiles
			Terra-cota tiles
			Terrazzo tiles
		Roof underlay	Synthetic roof underlay
			Tar saturated felt underlay no15
			Tar saturated felt underlay no30
		Straw roof	Straw thatch
		Weatherproofing membrane roofing	Breather filter fleece PP 1mm
			Breather HDPE HDPP 0.45mm
			Vapour control layer PE 0.125mm
In situ cementitious	Cementitious mixtures	Grout	Cement grout
		Mortar	Cement lime mortar
			Cement mortar
			Lime mortar
			Plastering mortar
		Screed	Cement screed
			Reinforced floor screed
		Slurry	Cement slurry
	Concrete in situ	Concrete in situ general	Concrete in situ general
		Ligthweight concrete	Ligthweight concrete
Insulation	Batts and rolls	Aerogel batts rolls	Aerogel batts rolls
		Biomass batts rolls	Bats cellulose fiber
			Cork batt
			Denim wool batt
			Flax linseed batt
			Hemp wool batt
			Sheep wool batt
			Woodfiber bats
		Mineral batts rolls	Glasswool batts rolls
			Glasswool sound batt

Level 1	Level 2	Level 3	Level 4
			Slag wool batts rolls
			Stonewool batts rolls
		Plastic batts rolls	PE batts rolls
	Board	Biomass board	Cork board
			Woodfiber board
		Mineral board insulation	Cellular glass board
			Glasswool facade board
			Stonewool facade board
			Stonewool ground board
			Stonewool on plasterboard
			Stonewool roof board
		Plastic boards	EPS board
			MEPS board
			Phenolic foam 120
			Phenolic foam 160
			Phenolic foam 60
			Phenolic foam 80
			Phenolic foam general
			Phenolic insulation board
			PIR PUR board
			Rigid foam PE board
			XPS board
	General values insulation	Mineral wool general	Glasswool general
			Mineral wool general
		Polystyrene general	Polystyrene general
	Loose fill fibers	Biomass	Cellulose fiber loose
			Cotton fabric
			Cotton padding
			Hemp loose
			Sawdust
			Sheep wool loose
		Mineral loose fibers	Glasswool attic floor blown
			Glasswool flooring blown
			Glasswool wall blown
			Slag wool blown
			Stonewool attic floor blown

Level 1	Level 2	Level 3	Level 4
			Stonewool flooring blown
			Stonewool wall blown
	Loose fill granulates	Mineral loose granulates	Aerogel granules
			Glass granulate 1-2mm
			Glass granulate 2-4mm
			Glass granulate 4-8mm
			Glass granulate 8-16mm
			Glass granulate general
			Perlite expanded
			Vermiculite expanded
		Plastic beads	EPS beads
			Recycled plastic beads
	Spray foam	Cementitious foam	Cementitious foam
		Mandolite foam	Mandolite
		Polyurethane foam	Closed cell two pound
			Open cell helf pound
Paint sealants adhesives	Paint varnish	Paint	Paint general
			Solventborne
			Waterborne
		Varnish	Wood Varnish
	Sealant and adhesives	Asphalt bitumen	Asphalt Bitumen
			Road tar
			Straight run
		Resin	Epoxy resin
			Melamine Resin
			Phelonic Resin
			Silicone resin
			Urea formaldehyde resin
Steel and metals	Metal products	Metal doors windows curtain	Curtain wall mullions RoT
			Carbon steel ext door no glazing
			Stainless steel ext door no glazing
			Carbon steel int door no glazing

Level 1	Level 2	Level 3	Level 4
			Window aluminum frame
			PVC window frame steel part
		Metal sheets	Aluminum sheet profiled 0.7mm
			Aluminum sheet profiled 1mm
			Aluminum sheet profiled general
			Steel sheet flat 0.5mm
			Steel sheet flat 0.8mm
			Steel sheet profiled 0.5mm
			Steel sheet profiled 0.7mm
	Metals general	Aluminum	Aluminum general
		Copper	Copper general
		Iron	Iron general
		Lead	Lead general
		Nickel	Nickel general
		Steel	Steel general
		Tin	Tin general
		Zinc	Zinc general
	Steel framing and reinforcement	Reinforcement in concrete	Mesh
			Rebar
		Steel sections	Angle
			Bar
			Channel
			Column steel section
			Hollow section
			Rail
			Rectangle
			Round
			Section
			Square
			U-beam
		Steel studs RoT	Ceiling steel stud
			External wall steel stud
			Lightwall 100mm steel stud
			Lightwall 80mm steel stud
		Steel truss RoT	Roof steel truss

**Level 1**	**Level 2**	**Level 3**	**Level 4**
Timber and engineered timber	Engineered timber	Engineered timber products	Glulam
			I-beam joist
			Laminated strand lumber
			Laminated veneer lumber
			Wood plastic composite
	Sawnwood species	Hardwood	Ash
			Balsa
			Bamboo
			Birch
			Cherry
			Hardwood average
			Hickory
			Mahogany
			Maple
			Oak
			Rosewood
			Walnut
		Softwood	Aspen
			Cedar
			Fir
			Hemlock
			Larch
			Pine
			Redwood
			Softwood average
			Spruce
	Timber general	Timber average value	Timber average
	Timber strength classes	C class	C14
			C16
			C18
			C20
			C22
			C24
			C27
			C30
			C35
			C40
			C45
			C50
		D class	D18

Level 1	Level 2	Level 3	Level 4
			D24
			D27
			D30
			D35
			D40
			D45
			D50
			D55
			D60
			D65
			D70
			D75
			D80
		T class	T14.5
			T10
			T11
			T12
			T13
			T14
			T15
			T16
			T18
			T21
			T22
			T24
			T26
			T27
			T28
			T30
			T8
			T9
		TR	TR26
	Wood product	Insulations	Sawdust
			Wool
			Jute felt
			Woodfiber bats
			Woodfiber board
			Cork batt
		Boards	Chipboard
			Cork board
			Fiberboard
			Hardboard HDF
			Hardwood lining

**Level 1**	**Level 2**	**Level 3**	**Level 4**
			Laminate
			MDF
			OSB
			Paperboard
			Particle board
			Plywood
			Wood plastic composite board
			Parquet
		Doors	Wood door ext
			Wood door int
			Softwood lining
Weatherproofing and plastics	Flat roof membranes	Bituminous roof membrane	Bottom layer
			Single layer
			Top layer
		Synthetic roof membrane	EPDM rubber roofing membrane
			PVC roofing membrane
			TPO roofing membrane
	Plastic general	Per plastic type	ABS
			General PE
			Plastic general
			PVC
		Plastic film	Film HDPE
			Film LDPE
			Orientated film PP
			UPVC film
	Roof underlays	Synthetic underlay	Synthetic roof underlay
		Tar saturated felt	Tar saturated felt underlay no15
			Tar saturated felt underlay no30
	Weatherproofing membranes	Breather membrane	Breather filter fleece PP 1mm
			Breather HDPE HDPP 0.45mm
		Damp proofing	Damp proof course PE 0.46mm
			Damp proof membrane LDPE 0.3mm
		Vapour control layer	Vapour control layer PE 0.125mm

**Level 1**	**Level 2**	**Level 3**	**Level 4**
Windows and doors	Door external	Glazing door	Double glazing
			Glass general value
			Single glazing
			Triple glazing
		No glazing	Carbon steel ext door no glazing
			Stainless steel ext door no glazing
			Wood door ext
	Door internal	Carbon steel door	Carbon steel int door no glazing
		Wood door	Wood door int
	Window	Aluminum frame	Window aluminum frame
		Glazing	Double glazing
			Glass general value
			Single glazing
			Triple glazing
		PVC frame	PVC window frame PVC part
			PVC window frame steel part
		Timber frame	Timber frame

2. RESULT SUMMARY

Table A2 Material classification used for the tab “Result summary” in BUD-MI. n.e.c. Not elsewhere classifiable.

Materials
Aggregates, sand, stones
Bio-based
Bricks and ceramics
Concrete and cementitious
Glass-based
Gypsum-based
Insulation wools
Metal - others
Metal - steel
Plastics
Others n.e.c

3. Economy-wide Material Flow Analysis

Table A3 Material classification in economy-wide material flow analysis (Eurostat, 2018).

Level 1	Level 2	Level 3	Level 4
MF.1 Biomass	MF.1.1 Crops, raw and processed	MF.1.1.1 Cereals	MF.1.1.1.1 Rice
			MF.1.1.1.2 Wheat
			MF.1.1.1.3 Maize
			MF.1.1.1.4 Cereals n.e.c.
		MF.1.1.2 Roots, tubers	
		MF.1.1.3 Sugar crops	
		MF.1.1.4 Pulses	
		MF.1.1.5 Nuts	
		MF.1.1.6 Oil bearing crops	
		MF.1.1.7 Vegetables	
		MF.1.1.8 Fruits	
		MF.1.1.9 Fibers	
		MF.1.1.10 Spice, beverage, pharmaceutical crops	
		MF.1.1.11 Tobacco	
		MF.1.1.12 Other crops n.e.c.	
	MF.1.2 Crop residues (used) and fodder crops	MF.1.2.1 Straw	
		MF.1.2.2 Other crop residues (sugar and fodder beet leaves, other)	
		MF.1.2.3 Fodder crops (including biomass harvest from grassland)	
	MF.1.3 Wood and wood products	MF.1.3.1 Timber (Industrial roundwood)	
		MF.1.3.2 Wood fuel and other extraction	
	MF.1.4 Wild fish, aquatic animals and plants	MF.1.4.1 Wild fish catch	
		MF.1.4.2 All other wild aquatic animals	
		MF.1.4.3 Aquatic plants	
	MF.1.5 Live animals and products (excl. wild fish, aquatic animals and plants)	MF.1.5.1 Live animals (excl. wild fish and animals)	
		MF.1.5.2 Meat and meat preparations	
		MF.1.5.3 Dairy products, bird eggs, and honey	
		MF.1.5.4 Other products from animals	
	MF.1.c Mixed / compounded products mainly from biomass		
MF.2 Metal ores	MF.2.Fe Iron ores and concentrates, iron and steel, products dominated by iron content		
	MF.2.Al Aluminum ores and concentrates, aluminum metal, products dominated by aluminum content		
	MF.2.x X ores and concentrates,	MF.2.Cu Copper ores metal content	
		MF.2.Ni Nickel ores metal content	

			X metal, products dominated by X	MF.2.Sn	Tin ores metal content		
				MF.2.Zn	Zinc ores metal content		
				MF.2.x	X ores metal content, where X is a specific metallic element other than iron or aluminum (memo item)		
		MF.2.c	Mixed / compounded products mainly from metal				
MF.3	Non-metallic minerals	MF.3.2	Carbonate minerals important in cement	MF.3.2.1	Chalk		
				MF.3.2.2	Dolomite		
				MF.3.2.3	Limestone		
				MF.3.2.4	Cement and its products		
		MF.3.7	Clays	MF.3.7.1	Structural clays and their products		
				MF.3.7.2	Specialty clays		
		MF.3.8	Sand and gravel	MF.3.8.1	Industrial sand and gravel		
				MF.3.8.2	Sand and gravel for construction		
		MF.3.c	Mixed / compounded products mainly from non-metallic mineral				
MF.4	Fossil fuels	MF.4.1	Coal and peat	MF.4.1.1	Brown coal	MF.4.1.1.1	Lignite (brown coal)
						MF.4.1.1.2	Other sub-bituminous coal
				MF.4.1.2	Hard coal	MF.4.1.2.1	Anthracite
						MF.4.1.2.2	Coking coal
						MF.4.1.2.3	Other bituminous coal
				MF.4.1.3	Peat		
				MF.4.1.4	Coal derived products n.e.c.		
		MF.4.2	Conventional petroleum and gas	MF.4.2.1	Crude oil and liquid petroleum products		
				MF.4.2.2	Natural gas and gaseous petroleum products		
		MF.4.c	Mixed / compounded products mainly from fossil fuels				
MF.5	Mixed / complex products n.e.c.						

4. Global Material Flow Analysis

Table A4 Material classification in global material flow analysis (International Resource Panel, 2024). Greyed out categories are not currently used in BUD-MI because they are not relevant to construction material.

MFA_4	MFA_13
Biomass	• Crop Residues<br>• Crops<br>• Grazed biomass and fodder crops<br>• Wild catch and harvest<br>• Wood
Products from biomass	• Non-wild animal products<br>• Products mainly from biomass nec.
Excavated earthen materials (including soil) nec	• Excavated earthen materials (including soil) nec
Fossil fuels	• Coal<br>• Natural Gas<br>• Oil shale and tar sands<br>• Petroleum
Products from fossil fuels	• Other products mainly from fossil fuels e.g. plastics<br>• Refined fossil fuels mainly for fuel e.g. LPG gasoline diesel
Metal ores	• Ferrous ores<br>• Non-ferrous ores
Products from metals	• Products mainly from metals nec.
Mixed and complex products nec.	• Mixed / complex products nec.
Non-metallic minerals	• Non-metallic minerals - construction dominant<br>• Non-metallic minerals - industrial or agricultural dominant
Products from non-metallic minerals	• Products mainly from non-metallic minerals
Waste for final treatment and disposal	• Waste for final treatment and disposal

5. Open Material Intensity Database Seed

Table A5 Classification of materials in the open MI database seed, as stated by (Heeren & Fishman, 2019). n.e.c. Not elsewhere classifiable.

**Level 1**	**Level 2**	**Level 3**	**Level 4**
Total without other materials	Bio-based	Bio-based	Paper cardboard<br>Straw<br>Wood
	Construction mineral	Concrete, cement & aggregate	Aggregates<br>Cement<br>Concrete
		Other construction material	Adobe<br>Bitumen<br>Brick<br>Cement asbestos<br>Clay<br>Mortar plaster<br>Natural stone<br>Plaster board gypsum<br>Siding unspecified
	Metals	Metals	Aluminum<br>Copper<br>Steel<br>Unspecified metal
Other materials	Other materials	Other materials	Carpet<br>Ceramics<br>Glass<br>Heraklith<br>Insulation unspecified<br>Lineoleum<br>Mineral wool<br>Other unspecified material<br>Plastics<br>Polystyrene<br>PVC

6. EUROPEAN UNION LIST OF WASTE

Table A6 List of construction demolition and waste materials, as stated in European Waste Codes, chapter 17 (Eurostat, 2010). H Hazardous waste, NH Non-hazardous waste.

Sub-chapter		Entry		H (1) NH (0)
**Code**	**Description**	**Code**	**Description**	
17 01	Concrete, bricks, tiles and ceramics	17 01 01	concrete	0
		17 01 02	bricks	0
		17 01 03	tiles and ceramics	0
		17 01 06	mixtures of, or separate fractions of concrete, bricks, tiles and ceramics containing dangerous substances	1
		17 01 07	mixtures of concrete, bricks, tiles and ceramics other than those mentioned in 17 01 06	0
17 02	Wood, glass and plastic	17 02 01	wood	0
		17 02 02	glass	0
		17 02 03	plastic	0
		17 02 04	glass, plastic and wood containing or contaminated with dangerous substances	1
17 03	Bituminous mixtures, coal tar and tarred products	17 03 01	bituminous mixtures containing coal tar	1
		17 03 02	bituminous mixtures other than those mentioned in 17 03 01	0
		17 03 03	coal tar and tarred products	1
17 04	Metals (including their alloys)	17 04 01	copper, bronze, brass	0
		17 04 02	aluminum	0
		17 04 03	lead	0
		17 04 04	zinc	0
		17 04 05	iron and steel	0
		17 04 06	tin	0
		17 04 07	mixed metals	0
		17 04 09	metal waste contaminated with dangerous substances	1
		17 04 10	cables containing oil, coal tar and other dangerous substances	1
		17 04 11	cables other than those mentioned in 17 04 10	0
17 05	Soil (incl. excavated soil from contaminated sites), stones and dredging spoil	17 05 03	soil and stones containing dangerous substances	1
		17 05 04	soil and stones other than those mentioned in 17 05 03	0
		17 05 05	dredging spoil containing dangerous substances	1
		17 05 06	dredging spoil other than those mentioned in 17 05 05	0
		17 05 07	track ballast containing dangerous substances	1
		17 05 08	track ballast other than those mentioned in 17 05 07	0

17 06	Insulation materials and asbestos-containing construction materials	17 06 01	insulation materials containing asbestos	1
		17 06 03	other insulation materials consisting of or containing dangerous substances	1
		17 06 04	insulation materials other than those mentioned in 17 06 01 and 17 06 03	0
		17 06 05	construction materials containing asbestos	1
17 08	Gypsum-based construction material	17 08 01	gypsum-based construction materials contaminated with dangerous substances	1
		17 08 02	gypsum-based construction materials other than those mentioned in 17 08 01	0
17 09	Other construction and demolition wastes	17 09 01	construction and demolition wastes containing mercury	1
		17 09 02	construction and demolition wastes containing PCB (for example PCB-containing sealants, PCB-containing resin-based floorings, PCB-containing sealed glazing units, PCB-containing capacitors)	1
		17 09 03	other construction and demolition wastes (including mixed wastes) containing dangerous substances	1
		17 09 04	mixed construction and demolition wastes other than those mentioned in 17 09 01, 17 09 02 and 17 09 03	0

7. Industry-oriented Material Classification

Table A7 Industry-oriented classification of materials used in BUD-MI. MDF Medium density fiber, OSB Oriented strand board, PVC Polyvinyl Chloride.

Main category	Subcategory	Component name
Building materials	Binding agents and mortars	Binders and mortars in general
		Floor screed
		Mortar / Plaster - Dry mix
		Wet mix
	Building blocks and aggregates	Bricks/tiles
		Building blocks and aggregate in general
		Concrete blocks
		Crushed rock material
		Glass brick
		Gravel material
		Infill soil
		Lightweight aggregate block
		Lightweight aggregate bulk
		Lightweight concrete
		Natural stone
		Sand
	Chemico-technical goods	Asphalt and sealants
		Chemico-technical goods in general
	Insulating materials	Cellulose insulation
		Expanded foamed plastic
		Expanded foamed plastic, extruded
		Foam plastic
		Insulation materials in general
		Mineral (rock) wool
		Wood wool
	Reinforcement, steel and metal goods	Bar steel
		Metals
		Reinforcing steel
		Sheet metal
		Structural hollow sections and industrial piping
		Welded mesh reinforcement
	Roof and wall cladding	Asphalt roofing shingles
		Clay roof tiles
		Roof and wall cladding in general
		Roofing sheet

Main category	Subcategory	Component name
		Roofing tiles, concrete
Building materials (cont.)	Sheet materials	Board<br>Cement-based boards<br>Chipboard
	Sheet materials (cont.)	Gypsum wall boards<br>Laminated plastic sheet<br>MDF<br>OSB (smartply)<br>Panelling and lining boards<br>Plasterboards, wetroom<br>Plywood<br>Sheet materials in general<br>Veneer
	Weatherproofing systems, tape and sealing strip	Plastic film<br>Rubber sheeting<br>Underlay felt<br>Weatherproofing systems<br>Weatherproofing systems, tape and sealing strip in general
Fit-out materials and paints	Ceiling and wall systems	Ceiling and wall systems in general
	Ceramic goods	Ceramic floor tiles
	Flooring articles	Flooring materials in general<br>Laminate flooring<br>Linoleum flooring<br>Parquet flooring<br>Plastic flooring<br>Rubber flooring<br>Textile flooring
	Paint goods	Other paint
	Wallpapers	Wallpapers<br>Wallpapers in general
Interior decor and joinery articles	Doors	Doors in general
	Windows and glass goods	Glass goods<br>Plastic windows (PVC)<br>Window and glass goods in general
Timber products	Exterior cladding timber	Exterior cladding timber in general
	Stress-graded timber	Quality class C14<br>Quality class C35<br>Scaffolding timber

Main category	Subcategory	Component name
		Stress-graded timber in general
	Timber goods	Building timber in general
	Wood components	Glulam columns<br>Veneered wood<br>Wood components in general
n.e.c.	n.e.c.	n.e.c.
